# Enantioselective amination of 4-alkylisoquinoline-1,3(2*H*,4*H*)-dione derivatives†

**DOI:** 10.1039/d0ra07806a

**Published:** 2020-11-25

**Authors:** Cheng Cheng, Ying-Xian Li, Xue-Min Jia, Ji-Quan Zhang, Yong-Long Zhao, Wei Feng, Lei Tang, Yuan-Yong Yang

**Affiliations:** State Key Laboratory of Functions and Applications of Medicinal Plants, Guizhou Provincial Engineering Technology Research Center for Chemical Drug R&D, School of Pharmacy, Guizhou Medical University Guiyang 550004 China yangyuanyong@gmc.edu.cn tlei1974@hotmail.com; BGI-Shenzhen Building 11, Beishan Industrial Zone, Yantian Shenzhen 518083 China; Guizhou Provincial Key Laboratory of Pathogenesis and Drug Research on Common Chronic Diseases, Guizhou Medical University Guiyang 550004 China

## Abstract

A mild and efficient enantioselective amination of 4-alkylisoquinoline-1,3(2*H*,4*H*)-dione derivatives was established, which is compatible with a broad range of substrates and delivers the final products in excellent yields (up to 99%) and ee values (up to 99%) with low catalyst loading (down to 1 mol%). The synthetic potential of this methodology was also demonstrated in the gram scale level.

## Abstract 2

A mild and efficient enantioselective amination of 4-alkylisoquinoline-1,3(2*H*,4*H*)-dione derivatives was established and a broad range of amination products were prepared in excellent yields and ee values with low catalyst loading.

## Graphical abstract

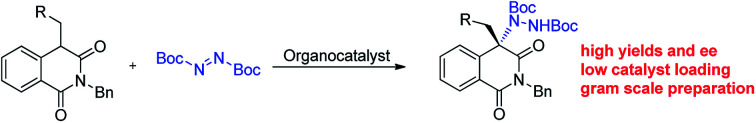

Isoquinolinedione, bearing the carbon skeleton of tetrahydroisoquinoline (THIQ), is an important structural motif present in bioactive compounds and natural products with a broad array of biological properties.^1^ However, the construction of isoquinolinedione, particularly the chiral version, is currently underdeveloped,^2^ and the reported methods heavily rely on the radical-initiated addition–cyclization of activated alkenes to prepare this structural motif that hard to be further diversified.^3^ From a pharmaceutical point of view, the presence of heteroatoms (such as nitrogen) is essential for their biological activity (Fig. 1).^4^ Therefore, the introduction of other functional groups or heteroatoms into this framework is a pressing issue to be addressed.

**Fig. 1:**
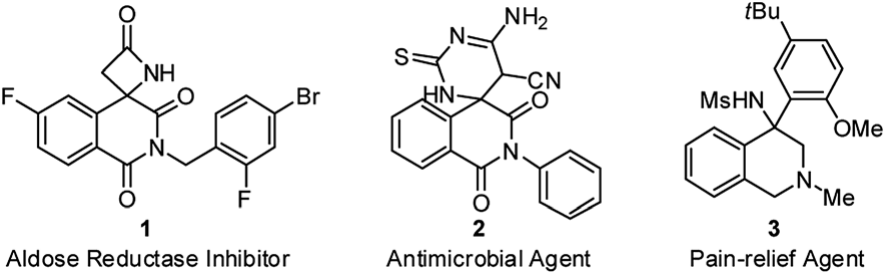
Bioactive compounds bearing isoquinolinedione or tetrahydroisoquinoline core structure.

On a different note, amine attached to a stereogenic center is a ubiquitous structure in natural products and bioactive compounds and becomes impetus for continuous exploration.^5^ Using azodicarboxylates or nitrosoarenes as electrophilic amine sources,^6^ activated substrates such as 1,3-dicarbonyl compounds and pyrazolones could be readily transformed into the corresponding amination products in high ee and yields.^7^ With the pioneering work of List and Jørgensen, the α-amination of aldehydes could be realized through enamine activation.^8^ The α-amination of less activated substrates such as nitroisoxazole derivatives could be realized *via* phase-transfer catalysis.^9^ Recently, cyclic ketones or vinyl ketones were transformed into the corresponding amination products *via* organo- or metal catalysis.^10^ Surprisingly, reports on the amination of heterocyclic compounds are very limited, and they majorly focus on the oxindole scaffold.^11^ Therefore, the construction of other pharmaceutical relevant α-amination heterocyclic compounds would be a meaningful work.^12^ In addition, the organo-catalyzed asymmetric amination reactions generally require relatively high catalyst loading to achieve the optimal yields and enantioselectivities; for this reason, the development of an efficient amination protocol would still be highly desirable.

Recently, our group reported the amination of 4-arylisoquinolinedione *via* organo-catalysis.^13^ However, due to the attenuated reactivity at low temperatures, high catalyst loading is required for satisfactory yields and enantioselectivities, and the substrate scope is limited to 4-aryl substituents. To further expand the scope of this reaction, we tried to extend this amination methodology to 4-alkylisoquinolinedione derivatives.

Our study commenced with 2-benzyl-4-butylisoquinoline-1,3(2*H*,4*H*)-dione 4a and di-*tert*-butyl azodicarboxylate 5 as model substrates for condition optimization (Table 1). With previously optimized bifunctional catalyst 7, the reaction proceeds smoothly and delivers the amination product in moderate yields and excellent enantioselectivity after 24 h (Table 1, entry 1). Gratifyingly, the chemical yield could be increased by raising the temperature and maintaining the ee value (Table 1, entry 2). Further solvent optimization reveals that the polarity of solvents poses a positive effect on the chemical yield but negative effect on the ee value (Table 1, entries 3–5), indicating that the polar solvent may contribute to the stabilization of the enolate intermediate *via* dipole–dipole interactions but also interrupting the efficient interaction of the substrate with the catalyst.

**Table 1:** Condition optimization for the amination reaction

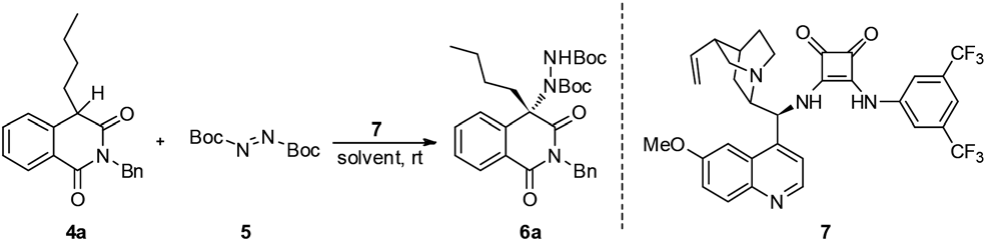				
Entry^a^	7 (mol%)	Solvent	Yield 6a^b^ (%)	ee^c^ (%)
1^d^	10	CHCl_3_	50	98
2	10	CHCl_3_	71	97
3	10	Toluene	82	74
4	10	Ether	95	80
5	10	THF	99	21
6	10	DCM	99	90
7	10	Chlorobenzene	85	94
8	10	CH_2_ClCH_2_Cl	99	98
9	5	CH_2_ClCH_2_Cl	97	97
10^e^	2	CH_2_ClCH_2_Cl	99	97
11^f^^,^^g^	1	CH_2_ClCH_2_Cl	83	95
12^f^^,^^g^	1	CH_2_ClCH_2_Cl	88	93

a All reaction was conducted with 0.2 mmol compound 4a, 0.44 mmol compound 5, in 0.5 mL solvent and reacted at 25 °C for 24 h.

b Isolated yield.

c The ee was determined by HPLC analysis.

d Reaction was conducted at 5 °C and reacted for 24 h.

e Reaction was run for 35 h.

f Reaction was reacted for 72 h.

g Reaction was conducted at 40 °C.

Further solvent optimization reveals that DCM gives the best yield along with very good ee (Table 1, entry 6). Then, another chlorinated solvent was tested and found that 1,2-dichloroethane gives the best results both in ee and yield (Table 1, entry 7 and 8). At this point, we try to study the catalyst loading effect on this amination reaction. At lower catalyst loadings, the ee dropped marginally and also the chemical yield, while the decrease in the chemical yield could be compensated by longer reaction time (Table 1, entries 9 and 10). We also tried to further decrease the catalyst loading to 1 mol%, but a much longer reaction time was required to get a satisfactory yield (Table 1, entry 11). The chemical yield could be increased slightly when the reaction was conducted at 40 °C; however, at the expense of ee (Table 1, entry 12). Therefore, taking account of the yield and ee of the final product, the 2 mol% catalyst at room temperature in 1,2-dichloroethane was established as under optimal reaction conditions for further exploration.

With a set of optimal reaction conditions in hand, the substrate scope for this amination reaction was explored. By changing the linear *n*-butyl to branched or substituted alkyl groups, the final products were obtained in very good yields and ee values (Table 2, entries 1–4). Except the meta-substituted benzyl groups, other benzyl groups generally give excellent yields and ee values regardless of the electronic or steric properties of the aromatic rings (Table 2, entries 5–16). Moreover, this methodology is also compatible with other steric or heteroaromatic substrates and excellent results were obtained (Table 2, entries 17–21). The absolute configuration of 6i determined *via* single crystal X-ray diffraction was *S*, and the absolute configurations of other products 6 were assigned by analogy.^14^

**Table 2:** Substrate scope for the amination reaction

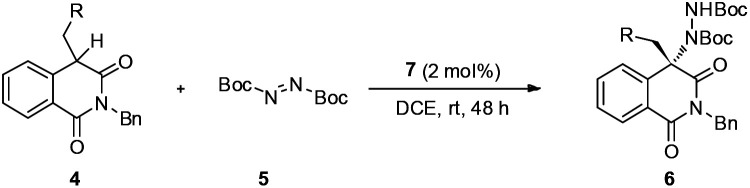				
Entry^a^	R	Product	Yield^b^ (%)	ee^c^ (%)
1	*n*-Propyl	6a	99	97
2	*i*-Butyl	6b	96	94
3	*i*-Propyl	6c	99	81
4	PhCH_2_CH_2_	6d	94	93
5	Ph	6e	99	97
6	4-MeC_6_H_4_	6f	99	92
7	4-OMeC_6_H_4_	6g	90	97
8	4-FC_6_H_4_	6h	96	96
9	4-ClC_6_H_4_	6i	99	99
10	4-BrC_6_H_4_	6j	99	99
11	3-MeC_6_H_4_	6k	99	76
12	3-BrC_6_H_4_	6l	99	89
13	2-OmeC_6_H_4_	6m	99	93
14	2-MeC_6_H_4_	6n	92	93
15	2-ClC_6_H_4_	6o	95	98
16	3,4,5-OmeC_6_H_2_	6p	99	87
17	2-Naphthyl	6q	99	99
18	2-Indolyl	6r	90	82
19	3-Indolyl	6s	99	99
20	2-Me-3-indolyl	6t	99	96
21	2-Fural	6u	99	97

a Reactions were run on a 0.03 mmol 1 and 0.036 mmol 2 with the 2 mol% catalyst in 500 μL solvent at 25 °C for 48 h.

b Yield was based on the isolated product of 3.

c The ee was determined *via* HPLC analysis.

To demonstrate the practical synthetic application of current protocol, the gram scale synthesis of chiral 6i has been demonstrated (Scheme 1). The product was produced in excellent yield and ee value at the 2 mmol scale. Moreover, a synthetically desirable amino product could be obtained from the cleavage of the N–N bond and deprotection of the Boc group in two steps with very good yield and ee value (Scheme 2).^15^

**Scheme 1:**
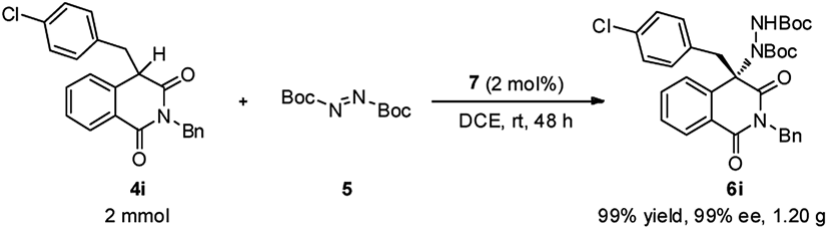
Gram scale preparation of 6i.

**Scheme 2:**
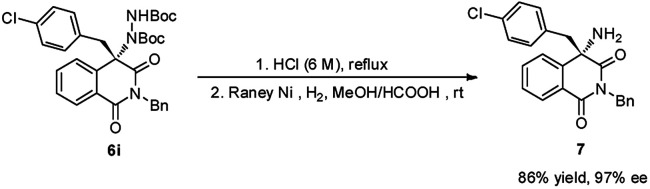
Transform the product into amino product.

In an effort to account for the observed stereocontrol of the reaction, a plausible reaction mechanism is proposed in Scheme 3. With the previously established bifunctional catalyst by Rawal *et al.*,^16^ the isoquinolinedione was activated by the alkyl amine moiety to attack the azodicarboxylate that was activated by the squaramide moiety *via* hydrogen bonding interactions in a well-defined manner to deliver the final product in *S* configuration.^17^ The outcome in this study is in accordance with our previous reports^13^ as the benzyl group alleviates the steric hindrance of the substituted phenyl ring from the reaction center and delivers the product in a high ee value (Table 2, entry 14).

**Scheme 3:**
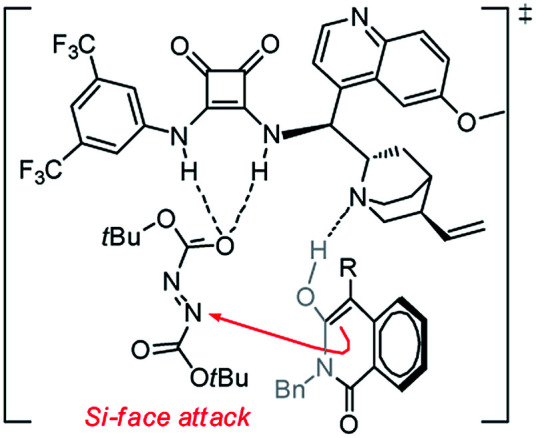
Proposed mechanism for the amination reaction.

To summarize, a highly enantioselective amination methodology with low catalyst loading was established (down to 1 mol%), which is compatible with a broad range of substrates and delivers the final products in excellent yields (up to 99%) and ee values (up to 99%). Moreover, the maintaining of yield and ee in up-scale preparation clearly demonstrates the synthetic potential of this methodology. Most importantly, this reaction is mild and operationally simple and could be performed without the exclusion of air or moisture at room temperature.

## Conflicts of interest

There are no conflicts to declare.

## Supplementary Material

RA-010-D0RA07806A-s001

RA-010-D0RA07806A-s002
