# Supplementary material for: Enantioselective amination of 4-alkylisoquinoline-1,3(2H,4H)-dione derivatives
Source: RSC Adv. 2020 Nov 25;10(70):42912–5. doi: 10.1039/d0ra07806a (PMC9058008; doi:10.1039/d0ra07806a)
Supplement: RA-010-D0RA07806A-s001 [file RA-010-D0RA07806A-s001.pdf]

Electronic Supplementary Material (ESI) for RSC Advances.
This journal is © The Royal Society of Chemistry 2020

Supporting Information

Enantioselective Amination of 4-Alkylisoquinoline-1,3(*2H,4H*)-dione Derivatives

Cheng Cheng, Ying-Xian Li, Xue-Ming Jia, Ji-Quan Zhang, Yong-Long Zhao, Wei Feng
Lei Tang,* and Yuan-Yong Yang*

State Key Laboratory of Functions and Applications of Medicinal Plants, School of Pharmacy, Guizhou Provincial Engineering Technology Research Center for Chemical Drug R&D, Guizhou Medical University, Guiyang 550004, China.

**Corresponding authors: yangyuanyong@gmc.edu.cn; tlei1974@gmc.edu.cn*

Table of Contents

1. General Information	2
2. General Procedure for Synthesis of **6a-6u**	3
3. Procedure for Synthesis of **7**	3
4. Substrate Scope for the Amination reaction	4
5. Spectroscopic Data for starting materials	5
6. Spectroscopic Data for Products	12
7. Crystal data and structure refinement for **6I**	28
8. ^{1}H and ^{13}C NMR Spectra	29
9. HPLC spectra	68
10. References	92

1. General Information

Proton nuclear magnetic resonance (^{1}H NMR) spectra and carbon nuclear magnetic resonance (^{13}C NMR) spectra were recorded on INOVA 400 MHz spectrometer (400 MHz and 100 MHz). Chemical shifts for protons are reported in parts per million downfield from tetramethylsilane and are referenced to residual protium in the NMR solvent ($CDCl_3$: δ 0.00). Chemical shifts for carbon are reported in parts per million downfield from tetramethylsilane and are referenced to the carbon resonances of the solvent ($CDCl_3$: δ 77.0). Data are represented as follows: chemical shift, integration, multiplicity (br = broad, s = singlet, d = doublet, t = triplet, q = quartet, m = multiplet), coupling constants in Hertz (Hz). All high resolution mass spectra were obtained on a Waters G2-XSQTof mass spectrometer. Melting points were determined on a Tektronix X-4 melting point apparatus. Analytical TLC was performed using EM separations percolated silica gel 0.2 mm layer UV 254 fluorescent sheets.

Starting Materials. All solvents and inorganic reagents were from commercial sources and used without purification unless otherwise noted. Starting material and catalysts were prepared following the literature procedures.[1,2]

2. General Procedure for Synthesis of 6a-6u

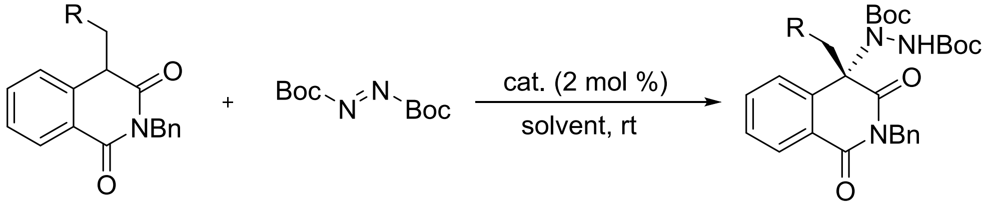

4 **5** **6**

Ditertbutyl azodicarboxylate **5** (0.036 mmol, 1.2 equiv.) and cat (2.0 mol%) was added to a miniature reaction bottle and the temperature was maintained at room temperature. After 5 min, **4** (0.03 mmol, 1 equiv.) was added and reacted for 48 h. The reaction was monitored by TLC analysis. Upon completion the solvent was removed by rotary evaporator and the crude product was purified by flash column chromatography (hexane/ethyl acetate = 50:1 to 20:1) to yield the desired product.

3. Procedure for Synthesis of 7

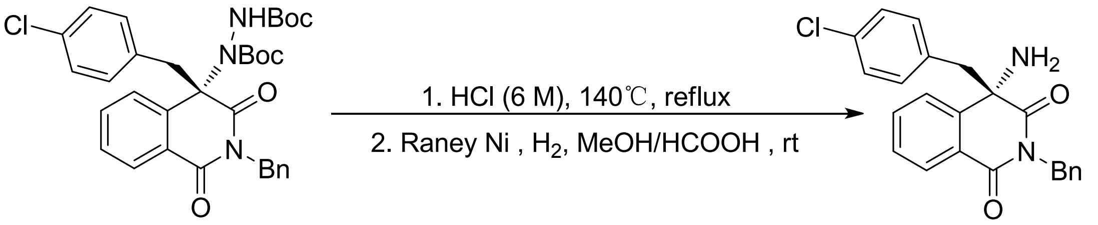

6i **7**

86% yield, 97% ee

The **6i** (0.03 mmol) was added in hydrochloric acid (1 mL, 6 M)) and the mixture was refluxed at 140 °C for 1 hour. The solvent was removed under reduced pressure, followed by cleavage of the hydrazine bond using Raney Ni and H_2 in methanol: Formic acid (9:1, 1 mL) afforded the **7** in 86% yield.

4. Substrate Scope for the Amination reaction

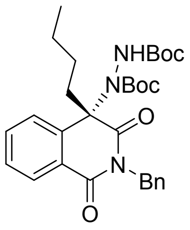
6a, 99% yield,ee 97%

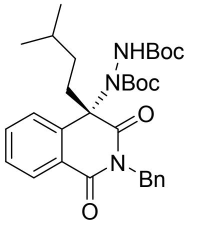
6b, 96% yield,ee 94%

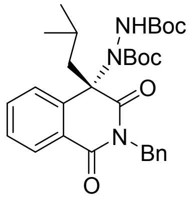
6c, 99% yield,ee 81%

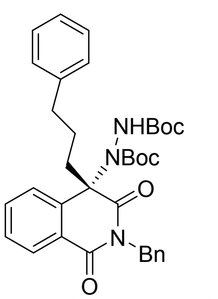
6d, 94% yield,ee 93%

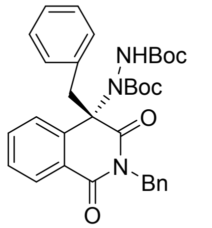
6e, 99% yield,ee 97%

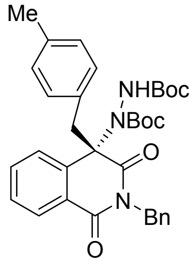
6f, 99% yield,ee 92%

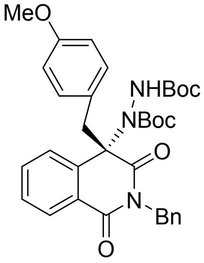
6g, 90% yield,ee 97%

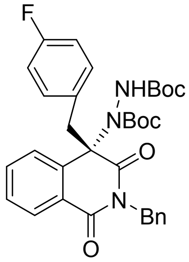
6h, 96% yield,ee 96%

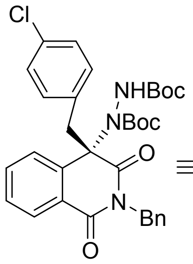
6i, 99% yield,ee 99%

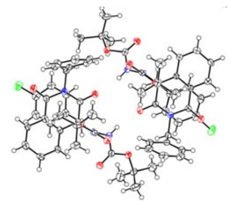
CDCC:2009628

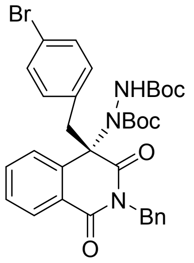
6j, 99% yield,ee 99%

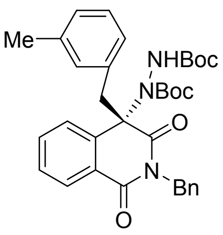
6k, 99% yield,ee 76%

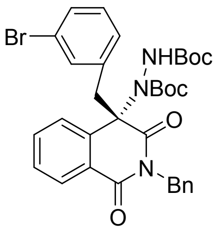
6l, 99% yield,ee 89%

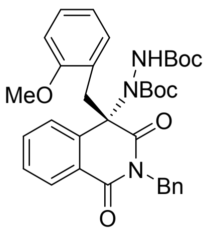
6m, 99% yield,ee 93%

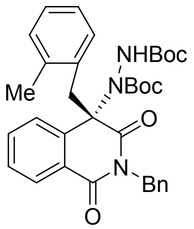
6n, 92% yield,ee 93%

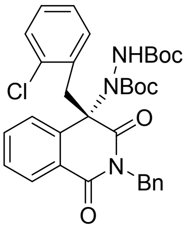
6o, 95% yield,ee 98%

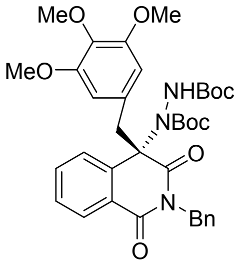
6p, 99% yield,ee 87%

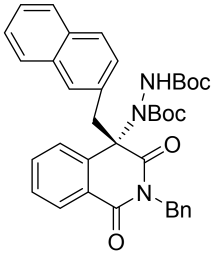
6q, 99% yield,ee >99.00%

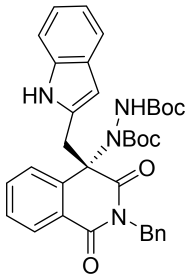
6r, 90% yield,ee 82%

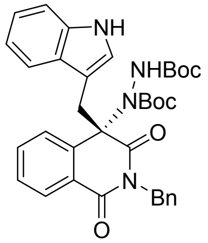
6s, 99% yield,ee 99%

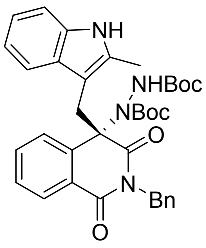
6t, 99% yield,ee 96%

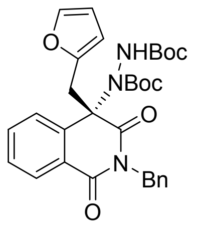
6u, 99% yield,ee 97%

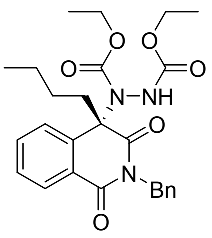
6v, 95% yield,ee 53%

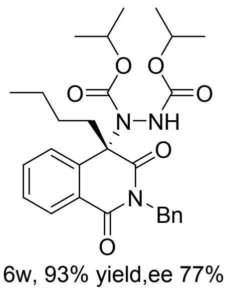
6w, 93% yield,ee 77%

5. Spectroscopic Data for starting materials

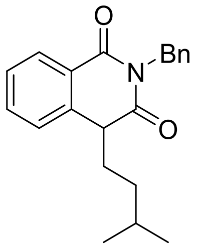

2-benzyl-4-isopentylisoquinoline-1,3(2H,4H)-dione (4b):

White oil, 327.4 mg, 51% yield;

^{1}H NMR ($CDCl_3$, 400 MHz): δ 8.16 (dd, *J* = 7.9, 1.3 Hz, 1H), 7.54 (td, *J* = 7.6, 1.4 Hz, 1H), 7.42 – 7.35 (m, 3H), 7.25 – 7.15 (m, 4H), 5.17 – 5.07 (m, 2H), 3.88 (t, *J* = 5.5 Hz, 1H), 2.09 (ddt, *J* = 13.3, 12.1, 5.1 Hz, 1H), 1.89 – 1.79 (m, 1H), 1.35 – 1.23 (m, 1H), 0.89 – 0.70 (m, 2H), 0.65 (dd, *J* = 8.6, 6.6 Hz, 6H);

^{13}C NMR ($CDCl_3$, 100 MHz): δ 173.7, 164.7, 139.3, 137.1, 133.7, 129.0, 128.3, 127.5, 127.4, 126.7, 125.5, 46.4, 43.2, 35.3, 33.6, 27.7, 22.3, 22.1;

HRMS (ESI): $C_{21}H_{24}NO_2$ for $[M+H]^+$, calculated 322.1807, found 322.1808;

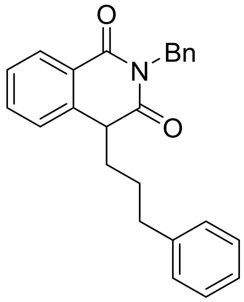

2-benzyl-4-(3-phenylpropyl)isoquinoline-1,3(2H,4H)-dione (4d):

White oil, 442.8 mg, 60% yield;

^{1}H NMR ($CDCl_3$, 400 MHz): δ 8.16 (d, *J* = 7.8 Hz, 1H), 7.56 – 7.49 (m, 1H), 7.39 (dd, *J* = 14.1, 7.1 Hz, 3H), 7.25 – 7.08 (m, 7H), 6.92 (d, *J* = 7.1 Hz, 2H), 5.13 (d, *J* = 1.1 Hz, 2H), 3.91 (t, *J* = 5.6 Hz, 1H), 2.51 – 2.33 (m, 2H), 2.20 – 2.10 (m, 1H), 1.96 – 1.85 (m, 1H), 1.31 – 1.19 (m, 2H);

^{13}C NMR ($CDCl_3$, 100 MHz): δ 173.5, 164.6, 141.2, 139.0, 137.1, 133.7, 129.0, 128.9, 128.4, 128.3, 128.2, 127.6, 127.5, 126.7, 125.9, 125.4, 46.1, 43.3, 36.7, 35.3, 26.5;

HRMS (ESI): $C_{25}H_{24}NO_2$ for $[M+H]^+$, calculated 370.1807, found 370.1809;

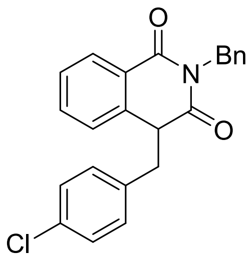

2-benzyl-4-(4-chlorobenzyl)isoquinoline-1,3(2H,4H)-dione (4i):

White solid, 427.5 mg, 57% yield. mp: 110.6-111.3 ℃;

^{1}H NMR ($CDCl_3$, 400 MHz): δ 8.03 (dd, *J* = 7.9, 1.3 Hz, 1H), 7.51 (td, *J* = 7.6, 1.4 Hz, 1H), 7.35 (ddd, *J* = 8.0, 7.5, 0.8 Hz, 1H), 7.29 – 7.23 (m, 2H), 7.23 – 7.17 (m, 3H), 7.14 (d, *J* = 7.7 Hz, 1H), 6.89 – 6.71 (m, 2H), 6.43 – 6.29 (m, 2H), 4.94 (q, *J* = 13.7 Hz, 2H), 4.13 (t, *J* = 5.4 Hz, 1H), 3.32 (dd, *J* = 13.3, 5.9 Hz, 1H), 3.14 (dd, *J* = 13.3, 4.9 Hz, 1H);

^{13}C NMR ($CDCl_3$, 100 MHz): δ 172.6, 164.1, 137.7, 136.7, 133.6, 133.5, 132.9, 130.5, 129.1, 129.0, 128.3, 128.3, 127.9, 127.5, 127.1, 125.8, 47.6, 43.3, 42.3;

HRMS (ESI): $C_{23}H_{19}NO_2Cl$ for $[M+H]^+$, calculated 376.1104, found 376.1105;

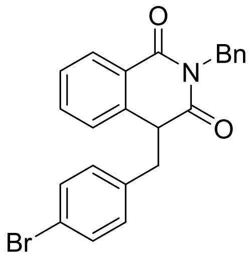

2-benzyl-4-(4-bromobenzyl)isoquinoline-1,3(2H,4H)-dione (4j):

White solid, 596.4 mg, 71% yield. mp: 103.2-105.1 ℃;

^{1}H NMR ($CDCl_3$, 400 MHz): δ 8.03 (dd, *J* = 7.9, 1.3 Hz, 1H), 7.50 (td, *J* = 7.6, 1.4 Hz, 1H), 7.34 (td, *J* = 7.8, 0.7 Hz, 1H), 7.27 – 7.17 (m, 5H), 7.13 (d, *J* = 7.7 Hz, 1H), 6.99 – 6.87 (m, 2H), 6.33 – 6.24 (m, 2H), 4.93 (q, *J* = 13.7 Hz, 2H), 4.12 (t, *J* = 5.4 Hz, 1H), 3.29 (dd, *J* = 13.3, 5.9 Hz, 1H), 3.11 (dd, *J* = 13.3, 4.9 Hz, 1H);

^{13}C NMR ($CDCl_3$, 100 MHz): δ 172.5, 164.1, 137.7, 136.7, 134.1, 133.5, 131.2, 130.8, 129.1, 129.0, 128.3, 127.9, 127.5, 127.1, 125.7, 121.1, 47.5, 43.3, 42.4;

HRMS (ESI): $C_{23}H_{19}NO_2Br$ for $[M+H]^+$, calculated 420.0599, found 420.0603;

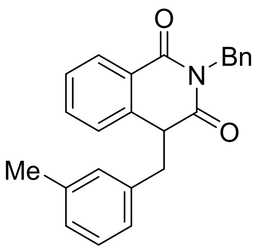

2-benzyl-4-(3-methylbenzyl)isoquinoline-1,3(2H,4H)-dione (4k):

White solid, 433.1 mg, 61% yield. mp: 114.1-115.3 ℃;

^{1}H NMR ($CDCl_3$, 400 MHz): δ 8.00 (d, J = 7.8 Hz, 1H), 7.46 (td, J = 7.6, 1.1 Hz, 1H), 7.38 – 7.25 (m, 3H), 7.21 – 7.12 (m, 3H), 7.05 (d, J = 7.7 Hz, 1H), 6.83 (dt, J = 14.8, 7.5 Hz, 2H), 6.38 – 6.27 (m, 2H), 4.92 (s, 2H), 4.12 (t, J = 5.6 Hz, 1H), 3.20 (ddd, J = 18.1, 13.2, 5.6 Hz, 2H), 2.04 (s, 3H);

^{13}C NMR ($CDCl_3$, 100 MHz): δ 173.0, 164.3, 138.1, 137.6, 136.9, 135.3, 133.2, 130.0, 128.9, 128.7, 128.3, 128.0, 127.8, 127.6, 127.3, 127.3, 126.2, 125.8, 48.2, 43.4, 43.3, 21.2;

HRMS (ESI): $C_{24}H_{22}NO_2$ for $[M+H]^+$, calculated 356.1651, found 356.1652;

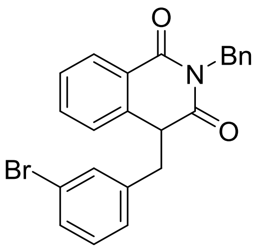

2-benzyl-4-(3-bromobenzyl)isoquinoline-1,3(2H,4H)-dione (4l):

White solid, 662.0 mg, 79% yield. mp: 117.7-119.0 ℃;

^{1}H NMR ($CDCl_3$, 400 MHz): δ 8.01 (d, J = 7.8 Hz, 1H), 7.52 – 7.45 (m, 1H), 7.34 (t, J = 7.6 Hz, 1H), 7.27 (d, J = 6.9 Hz, 2H), 7.21 – 7.12 (m, 4H), 7.07 (d, J = 7.7 Hz, 1H), 6.73 (dd, J = 16.8, 9.0 Hz, 2H), 6.40 (d, J = 7.7 Hz, 1H), 4.93 (s, 2H), 4.11 (t, J = 5.5 Hz, 1H), 3.25 (dd, J = 13.3, 6.1 Hz, 1H), 3.12 (dd, J = 13.3, 5.0 Hz, 1H);

^{13}C NMR ($CDCl_3$, 100 MHz): δ 172.6, 164.1, 137.7, 137.5, 136.8, 133.5, 132.3, 130.2, 129.7, 129.0, 129.0, 128.4, 127.9, 127.8, 127.4, 127.1, 125.7, 122.1, 47.8, 43.4, 42.8;

HRMS (ESI): $C_{23}H_{19}NO_2Br$ for $[M+H]^+$, calculated 420.0599, found 420.0598;

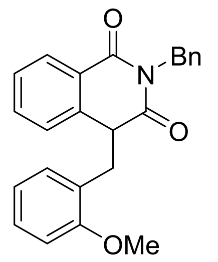

2-benzyl-4-(2-methoxybenzyl)isoquinoline-1,3(2H,4H)-dione (4m):

White solid, 526.8 mg, 71% yield. mp: 200.3-201.1 ℃;

^{1}H NMR ($CDCl_3$, 400 MHz): δ 8.02 (dd, J = 7.8, 1.4 Hz, 1H), 7.39 – 7.33 (m, 3H), 7.28 (td, J = 7.6, 1.1 Hz, 1H), 7.23 – 7.16 (m, 3H), 7.12 – 7.04 (m, 1H), 6.87 (dd, J = 7.6, 0.5 Hz, 1H), 6.62 (ddd, J = 11.9, 8.4, 4.6 Hz, 2H), 6.49 (dd, J = 7.4, 1.7 Hz, 1H), 5.01 (s, 2H), 4.14 (t, J = 6.6 Hz, 1H), 3.51 (s, 3H), 3.19 (dd, J = 13.0, 6.2 Hz, 1H), 3.07 (dd, J = 13.1, 7.0 Hz, 1H);

^{13}C NMR ($CDCl_3$, 100 MHz): δ 173.4, 164.7, 157.4, 138.7, 137.2, 132.7, 131.3, 129.0, 128.6, 128.5, 128.3, 127.6, 127.4, 125.3, 124.5, 124.0, 120.1, 110.0, 54.9, 47.6, 43.4, 38.9;

HRMS (ESI): $C_{24}H_{22}NO_3$ for $[M+H]^+$, calculated 372.1600, found 372.1601;

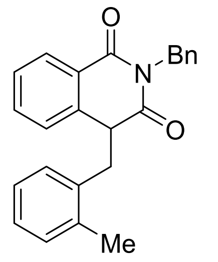

2-benzyl-4-(2-methylbenzyl)isoquinoline-1,3(2H,4H)-dione (4n):

White solid, 383.4 mg, 54% yield. mp: 108.5-109.7 ℃;

^{1}H NMR ($CDCl_3$, 400 MHz): δ 8.39 – 7.99 (m, 1H), 7.49 – 7.32 (m, 4H), 7.31 – 7.21 (m, 3H), 7.14 – 7.01 (m, 2H), 6.95 (t, J = 7.4 Hz, 1H), 6.77 – 6.50 (m, 2H), 5.15 – 5.04 (m, 2H), 4.09 (dd, J = 8.7, 4.9 Hz, 1H), 3.33 (dd, J = 13.4, 4.9 Hz, 1H), 3.04 (dd, J = 13.4, 8.8 Hz, 1H), 1.95 (s, 3H);

^{13}C NMR ($CDCl_3$, 100 MHz): δ 173.4, 164.6, 138.0, 137.0, 136.8, 134.4, 132.9, 130.4, 130.4, 129.1, 128.9, 128.4, 127.8, 127.6, 127.4, 127.2, 125.7, 125.3, 48.4, 43.5, 41.2, 19.0;

HRMS (ESI): $C_{24}H_{22}NO_2$ for $[M+H]^+$, calculated 356.1651, found 356.1653;

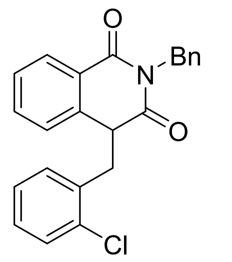

2-benzyl-4-(2-chlorobenzyl)isoquinoline-1,3(2H,4H)-dione (4o):

White solid, 517.5 mg, 69% yield. mp: 118.5-119.0 ℃;

^{1}H NMR ($CDCl_3$, 400 MHz): δ 8.10 (dd, *J* = 7.6, 1.6 Hz, 1H), 7.43 – 7.32 (m, 4H), 7.26 – 7.17 (m, 4H), 7.08 (td, *J* = 7.8, 1.6 Hz, 1H), 6.94 – 6.85 (m, 2H), 6.46 (dd, *J* = 7.6, 1.5 Hz, 1H), 5.06 (q, *J* = 13.8 Hz, 2H), 4.18 (t, *J* = 7.1 Hz, 1H), 3.18 (qd, *J* = 13.5, 7.1 Hz, 2H);

^{13}C NMR ($CDCl_3$, 100 MHz): δ 172.6, 164.6, 137.9, 137.1, 134.4, 134.1, 133.3, 131.9, 129.5, 129.1, 128.6, 128.4, 127.9, 127.5, 127.5, 126.5, 125.2, 47.3, 43.4, 41.8;

HRMS (ESI): $C_{23}H_{19}NO_2Cl$ for $[M+H]^+$, calculated 376.1104, found 376.1105；

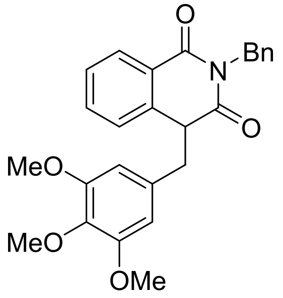

2-benzyl-4-(3,4,5-trimethylbenzyl)isoquinoline-1,3(2H,4H)-dione (4p):

Yellow solid, 459.6 mg, 60% yield. mp: 107.6-110.1 ℃;

^{1}H NMR ($CDCl_3$, 400 MHz): δ 8.11 (dd, *J* = 7.9, 1.2 Hz, 1H), 7.56 (td, *J* = 7.6, 1.4 Hz, 1H), 7.43 – 7.38 (m, 1H), 7.25 – 7.14 (m, 6H), 5.81 (s, 2H), 5.03 – 4.95 (m, 2H), 4.21 (t, *J* = 5.4 Hz, 1H), 3.75 (s, 3H), 3.51 (s, 6H), 3.30 – 3.25 (m, 2H);

^{13}C NMR ($CDCl_3$, 100 MHz): δ 172.9, 164.1, 152.7, 138.1, 137.0, 136.7, 133.3, 130.9, 128.9, 128.3, 127.7, 127.4, 127.3, 126.0, 106.2, 60.8, 58.4, 55.8, 48.1, 43.4;

HRMS (ESI): $C_{26}H_{25}NO_5Na$ for $[M+Na]^+$, calculated 454.1630, found 454.1629；

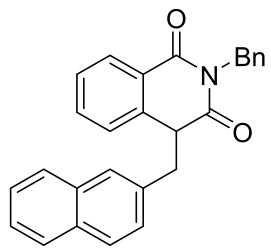

2-benzyl-4-(naphthalen-2-ylmethyl)isoquinoline-1,3(2H,4H)-dione (4q):

Yellow solid, 516.1 mg, 66% yield. mp: 155.6-155.8 ℃;

^{1}H NMR (CDCl$_3$, 400 MHz): δ 7.91 (d, *J* = 7.8 Hz, 1H), 7.58 – 7.53 (m, 1H), 7.39 – 7.32 (m, 3H), 7.27 – 7.21 (m, 3H), 7.14 – 7.09 (m, 2H), 7.00 (dt, *J* = 15.5, 6.8 Hz, 5H), 6.59 (dd, *J* = 8.4, 1.5 Hz, 1H), 4.85 (s, 2H), 4.14 (t, *J* = 5.7 Hz, 1H), 3.33 (qd, *J* = 13.3, 5.7 Hz, 2H);

^{13}C NMR (CDCl$_3$, 100 MHz): δ 172.9, 164.2, 138.0, 136.8, 133.3, 133.1, 133.1, 132.3, 128.9, 128.7, 128.3, 128.3, 127.8, 127.7, 127.6, 127.5, 127.3, 127.1, 126.0, 125.7, 125.6, 48.1, 43.4, 43.4;

HRMS (ESI): $C_{27}H_{22}NO_2$ for $[M+H]^+$, calculated 392.1651, found 392.1652；

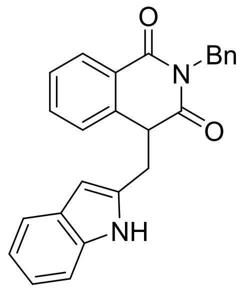

4-((1H-indol-2-yl)methyl)-2-benzylisoquinoline-1,3(2H,4H)-dione (4r):

Brown solid, 182.4 mg, 24% yield. mp: 139.7-142.4 ℃;

^{1}H NMR (CDCl$_3$, 400 MHz): δ 8.06 (d, *J* = 7.3 Hz, 1H), 7.74 (s, 1H), 7.50 (td, *J* = 7.6, 1.2 Hz, 1H), 7.33 (t, *J* = 7.6 Hz, 1H), 7.27 (d, *J* = 7.8 Hz, 1H), 7.20 (d, *J* = 7.7 Hz, 1H), 7.17 – 7.05 (m, 5H), 6.97 (d, *J* = 3.8 Hz, 2H), 6.93 – 6.88 (m, 1H), 5.72 (d, *J* = 1.7 Hz, 1H), 5.01 (s, 2H), 4.14 (t, *J* = 5.1 Hz, 1H), 3.51 (dd, *J* = 14.7, 5.4 Hz, 1H), 3.40 (dd, *J* = 14.7, 5.0 Hz, 1H);

^{13}C NMR (CDCl$_3$, 100 MHz): δ 173.3, 164.1, 137.7, 136.6, 135.9, 133.8, 133.3, 129.2, 128.5, 128.3, 128.1, 128.0, 127.4, 126.8, 125.4, 121.6, 120.1, 119.7, 110.7, 102.3, 46.3, 43.5, 34.3;

HRMS (ESI): $C_{25}H_{21}N_2O_2$ for $[M+H]^+$, calculated 381.1603, found 381.1605；

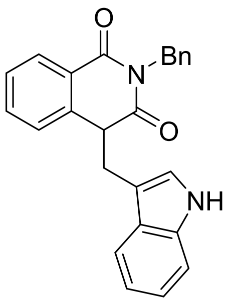

4-((1H-indol-3-yl)methyl)-2-benzylisoquinoline-1,3(2H,4H)-dione (4s):

Yellow solid, 281.2 mg, 37% yield. mp: 153.6-154.4 ℃;

^{1}H NMR ($CDCl_3$, 400 MHz): δ 8.03 (d, *J* = 7.8 Hz, 1H), 7.82 (s, 1H), 7.59 (dd, *J* = 10.8, 4.2 Hz, 1H), 7.41 (t, *J* = 7.6 Hz, 1H), 7.32 – 7.28 (m, 2H), 7.28 – 7.21 (m, 5H), 7.20 – 7.11 (m, 2H), 6.99 (t, *J* = 7.5 Hz, 1H), 6.19 (d, *J* = 2.2 Hz, 1H), 4.88 (dd, *J* = 31.3, 13.9 Hz, 2H), 4.35 – 4.27 (m, 1H), 3.66 (dd, *J* = 14.1, 6.1 Hz, 1H), 3.43 (dd, *J* = 14.1, 4.3 Hz, 1H);

^{13}C NMR ($CDCl_3$, 100 MHz): δ 173.6, 164.4, 138.9, 137.0, 135.6, 133.3, 128.9, 128.6, 128.2, 127.5, 127.3, 127.2, 127.1, 125.9, 123.2, 122.0, 119.5, 118.4, 111.0, 109.4, 47.8, 43.2, 33.7;

HRMS (ESI): $C_{25}H_{21}N_2O_2$ for $[M+H]^+$, calculated 381.1603, found 381.1601；

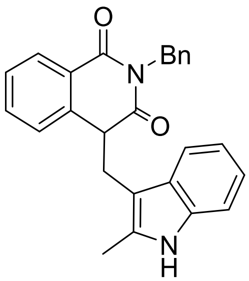

2-benzyl-4-((2-methyl-1H-indol-3-yl)methyl)isoquinoline-1,3(2H,4H)-dione (4t):

Yellow solid, 472.8 mg, 60% yield. mp: 175.9-176.2 ℃;

^{1}H NMR ($CDCl_3$, 400 MHz): δ 8.00 (dd, *J* = 7.7, 1.4 Hz, 1H), 7.67 (s, 1H), 7.42 (td, *J* = 7.5, 1.6 Hz, 1H), 7.36 (td, *J* = 7.5, 1.3 Hz, 1H), 7.28 – 7.13 (m, 7H), 7.09 – 7.02 (m, 1H), 7.00 – 6.89 (m, 2H), 4.84 (d, *J* = 14.1 Hz, 1H), 4.66 (d, *J* = 14.1 Hz, 1H), 4.21 (dd, *J* = 7.3, 4.1 Hz, 1H), 3.34 (ddd, *J* = 18.1, 14.0, 5.7 Hz, 2H), 1.49 (s, 3H).

^{13}C NMR ($CDCl_3$, 100 MHz): δ 174.0, 164.6, 138.9, 137.2, 135.1, 133.5, 133.1, 128.8, 128.7, 128.3, 128.3, 127.7, 127.6, 127.3, 125.9, 121.4, 119.5, 118.0, 110.3, 105.6, 48.4, 43.4, 33.8, 10.7;

HRMS (ESI): $C_{26}H_{23}N_2O_2$ for $[M+H]^+$, calculated 395.1760, found 395.1760；

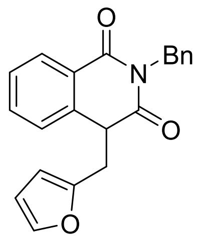

2-benzyl-4-(furan-2-ylmethyl)isoquinoline-1,3(2H,4H)-dione (4u):

Yellow solid, 331.0 mg, 50% yield. mp: 86.7-87.0 ℃;

^{1}H NMR ($CDCl_3$, 400 MHz): δ 8.01 (d, *J* = 7.6 Hz, 1H), 7.42 (td, *J* = 7.6, 1.0 Hz, 1H), 7.29 (dd, *J* = 15.3, 7.4 Hz, 3H), 7.19 – 7.11 (m, 3H), 7.00 (d, *J* = 7.7 Hz, 1H), 6.90 (d, *J* = 1.1 Hz, 1H), 5.99 – 5.83 (m, 1H), 5.43 (d, *J* = 3.1 Hz, 1H), 5.00 (q, *J* = 13.8 Hz, 2H), 4.09 (t, *J* = 5.5 Hz, 1H), 3.33 (dd, *J* = 14.7, 6.0 Hz, 1H), 3.20 (dd, *J* = 14.7, 5.2 Hz, 1H);

^{13}C NMR ($CDCl_3$, 100 MHz): δ 172.6, 164.3, 149.9, 141.8, 137.7, 136.9, 133.5, 129.1, 128.9, 128.7, 128.3, 127.7, 127.4, 126.9, 125.4, 110.1, 108.2, 46.0, 43.5, 35.1;

HRMS (ESI): $C_{21}H_{18}NO_3$ for $[M+H]^+$, calculated 332.1287, found 332.1290；

6. Spectroscopic Data for Products

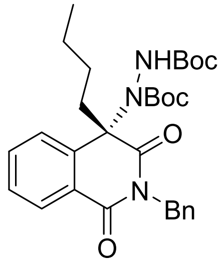

di-tert-butyl(S)-1-(2-benzyl-4-butyl-1,3-dioxo-1,2,3,4-tetrahydroisoquinolin-4-yl)hydrazine-1,2-dicarboxylate (6a):

White solid, 15.9 mg, 99% yield. mp: 115.9-118.2 ℃;

Optical rotation: [α]26.2 D= -11.03 (c 1.7, $CHCl_2$);

^{1}H NMR ($CDCl_3$, 400 MHz): δ 8.23 – 8.14 (m, 2H), 7.63 (t, *J* = 7.2 Hz, 1H), 7.41 (dd, *J* = 19.6, 11.8 Hz, 3H), 7.25 – 7.19 (m, 3H), 6.70 (s, 1H), 5.17 (d, *J* = 13.7 Hz, 1H), 5.11 (d, *J* = 13.6 Hz, 1H), 2.06 (dd, *J* = 36.5, 7.1 Hz, 2H), 1.51 (s, 18H), 1.23 (s, 2H), 1.00 (dd, *J* = 7.3, 3.2 Hz, 2H), 0.59 (t, *J* = 7.3 Hz, 3H);

^{13}C NMR ($CDCl_3$, 100 MHz): δ 175.7, 167.0, 164.1, 156.5, 143.3, 136.6, 134.4, 129.5, 128.3, 128.2, 127.5, 125.6, 124.5, 82.9, 81.3, 43.7, 29.7, 28.2, 25.1, 22.5, 13.5;

HRMS (ESI): $C_{30}H_{39}N_3O_6Na$ for $[M+Na]^+$, calculated 560.2737, found 560.2735；

The enantiomeric excess was determined to be 97.40% by HPLC. [IC column, 230 nm, n-hexane:IPA = 95:5, 0.5 mL/min]: 12.4 min (major), 15.6 min (minor);

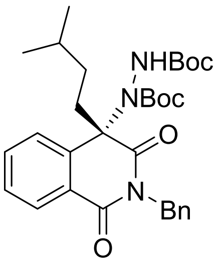

di-tert-butyl(S)-1-(2-benzyl-4-isopentyl-1,3-dioxo-1,2,3,4-tetrahydroisoquinolin-4-yl)hydrazine-1,2-dicarboxylate (6b):

White solid, 15.9 mg, 96% yield. mp: 139.6-140.6 ℃;

Optical rotation: [α]26.1 D= -10.93 (c 1.2, $CHCl_2$);

^{1}H NMR ($CDCl_3$, 400 MHz): δ 8.17 (dd, *J* = 14.8, 7.4 Hz, 2H), 7.63 (t, *J* = 7.2 Hz, 1H), 7.49 – 7.36 (m, 3H), 7.21 (dt, *J* = 12.7, 3.7 Hz, 3H), 6.69 (s, 1H), 5.18 (d, *J* = 13.6 Hz, 1H), 5.08 (d, *J* = 13.6 Hz, 1H), 2.08 (d, *J* = 11.4 Hz, 1H), 1.87 – 1.76 (m, 1H), 1.58 (s, 1H), 1.55 (s, 2H), 1.50 (s, 18H), 0.55 (dd, *J* = 6.6, 2.0 Hz, 6H);

^{13}C NMR ($CDCl_3$, 100 MHz): δ 175.7, 164.2, 156.4, 150.0, 136.7, 134.5, 128.4, 128.2, 127.7, 127.6, 127.6, 125.7, 124.6, 82.9, 81.3, 43.7, 31.6, 28.3, 27.9, 27.5, 22.0;

HRMS (ESI): $C_{31}H_{41}N_3O_6Na$ for $[M+Na]^+$, calculated 574.2893, found 574.2895；

The enantiomeric excess was determined to be 94.07% by HPLC. [IC column, 230 nm, n-hexane:IPA = 97:3, 0.5 mL/min]: 15.7 min (major), 20.4 min (minor);

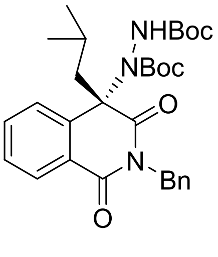

di-tert-butyl(S)-1-(2-benzyl-4-isobutyl-1,3-dioxo-1,2,3,4-tetrahydroisoquinolin-4-yl)hydrazine-1,2-dicarboxylate (6c):

White solid, 15.9 mg, 99% yield. mp: 139.9-141.1 ℃;

Optical rotation: [α]25.7 D= -21.93 (c 1.7, $CHCl_2$);

^{1}H NMR ($CDCl_3$, 400 MHz): δ 8.23 (d, *J* = 7.7 Hz, 1H), 8.17 (dd, *J* = 7.9, 0.9 Hz, 1H), 7.66 – 7.59 (m, 1H), 7.49 – 7.37 (m, 3H), 7.33 – 7.09 (m, 4H), 6.71 (s, 1H), 5.12 (s, 2H), 2.00 (dd, *J* =

13.6, 5.1 Hz, 1H), 1.84 (dd, J = 13.6, 4.4 Hz, 1H), 1.52 (s, 18H), 0.44 (d, J = 6.6 Hz, 3H), 0.39 (d, J = 6.7 Hz, 3H);

^{13}C NMR ($CDCl_3$, 100 MHz): δ 175.6, 164.1, 156.4, 143.3, 136.5, 134.3, 133.4, 128.4, 128.3, 127.6, 127.5, 126.0, 124.4, 82.9, 81.3, 49.9, 44.0, 28.3, 23.9, 22.9;

HRMS (ESI): $C_{30}H_{39}N_3O_6Na$ for $[M+Na]^+$, calculated 560.2737, found 560.2736；

The enantiomeric excess was determined to be 80.55% by HPLC. [IC column, 230 nm, n-hexane:IPA = 97:3, 0.5 mL/min]: 13.8 min (major), 19.6 min (minor);

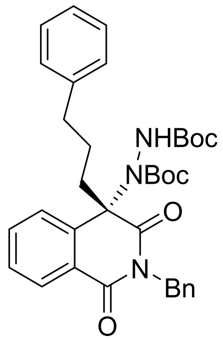

di-tert-butyl(S)-1-(2-benzyl-1,3-dioxo-4-(3-phenylpropyl)-1,2,3,4-tetrahydroisoquinolin-4-yl)hydrazine-1,2-dicarboxylate(6d):

White oil, 16.9 mg, 94% yield;

Optical rotation: [α]26.2 D= -17.88 (c 0.9, $CHCl_2$);

^{1}H NMR ($CDCl_3$, 400 MHz): δ 8.03 (d, J = 7.9 Hz, 1H), 7.51 (s, 1H), 7.29 (dd, J = 17.6, 9.9 Hz, 3H), 7.15 – 7.08 (m, 4H), 7.04 – 6.97 (m, 3H), 6.65 (d, J = 6.7 Hz, 2H), 6.58 (s, 1H), 5.01 (dd, J = 37.3, 13.6 Hz, 2H), 3.03 – 1.98 (m, 4H), 1.97 – 1.64 (m, 2H), 1.39 (s, 18H);

^{13}C NMR ($CDCl_3$, 100 MHz): δ 175.5, 164.1, 154.1, 140.7, 136.6, 134.5, 129.5, 128.6, 128.4, 128.2, 128.1, 127.7, 125.9, 125.7, 124.5, 82.9, 81.4, 43.7, 35.4, 29.7, 28.3, 24.5;

HRMS (ESI): $C_{35}H_{41}N_3O_6Na$ for $[M+Na]^+$, calculated 622.2893, found 622.2892；

The enantiomeric excess was determined to be 93.23% by HPLC. [IC column, 230 nm, n-hexane:IPA = 97:3, 1.0 mL/min]: 9.9 min (major), 15. 8 min (minor);

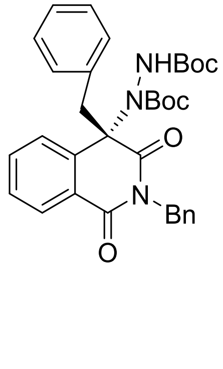

(di-tert-butyl(S)-1-(2,4-dibenzyl-1,3-dioxo-1,2,3,4-tetrahydroisoquinolin-4-yl)hydrazine-1,2-dicarboxylate (6e):

White solid, 17.0 mg, 99% yield. mp: 167.8-168.6 ℃;

Optical rotation: [α]25.6 D= -15.24 (c 1.8, $CHCl_2$);

^{1}H NMR ($CDCl_3$, 400 MHz): δ 8.39 (d, *J* = 6.7 Hz, 1H), 7.77 (d, *J* = 7.7 Hz, 1H), 7.65 (t, *J* = 7.4 Hz, 1H), 7.39 – 7.24 (m, 3H), 7.15 (dd, *J* = 12.8, 5.3 Hz, 3H), 6.98 (dd, *J* = 12.6, 5.3 Hz, 1H), 6.79 (dd, *J* = 19.4, 11.8 Hz, 3H), 6.17 (d, *J* = 7.4 Hz, 2H), 4.85 (d, *J* = 13.5 Hz, 1H), 4.68 (d, *J* = 13.5 Hz, 1H), 3.58 (d, *J* = 12.0 Hz, 1H), 3.01 (d, *J* = 12.1 Hz, 1H), 1.48 (s, 18H);

^{13}C NMR ($CDCl_3$, 100 MHz): δ 174.9, 163.0, 156.7, 142.6, 137.5, 136.3, 134.2, 132.1, 130.0, 128.2, 127.7, 127.5, 125.8, 125.3, 124.3, 123.5, 83.1, 81.5, 47.4, 43.7, 28.3;

HRMS (ESI): $C_{33}H_{37}N_3O_6Na$ for $[M+Na]^+$, calculated 594.2580, found 594.2581;

The enantiomeric excess was determined to be 97.45% by HPLC. [IC column, 230 nm, n-hexane:IPA = 98:2, 0.3 mL/min]: 40.8 min (major), 48.0 min (minor);

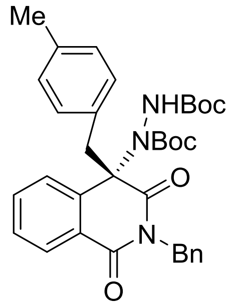

di-tert-butyl(S)-1-(2-benzyl-4-(4-methylbenzyl)-1,3-dioxo-1,2,3,4-tetrahydroisoquinolin-4-yl)hydrazine-1,2-dicarboxylate (6f):

Yellow solid, 17.4 mg, 99% yield. mp: 98.5-100.2 ℃;

Optical rotation: [α]25.1 D= -7.91 (c 1.6, $CHCl_2$);

^{1}H NMR ($CDCl_3$, 400 MHz): δ 8.45 (d, *J* = 7.1 Hz, 1H), 7.90 – 7.83 (m, 1H), 7.71 (t, *J* = 7.1 Hz, 1H), 7.38 (dd, *J* = 14.8, 7.0 Hz, 3H), 7.23 (dd, *J* = 13.0, 5.5 Hz, 3H), 6.87 (s, 1H), 6.65 (d, *J* = 7.8 Hz, 2H), 6.13 (d, *J* = 7.9 Hz, 2H), 4.93 (d, *J* = 13.6 Hz, 1H), 4.77 (d, *J* = 13.6 Hz, 1H), 3.62 (d, *J* = 12.1 Hz, 1H), 3.05 (d, *J* = 12.2 Hz, 1H), 2.16 (s, 3H), 1.55 (s, 18H);

^{13}C NMR ($CDCl_3$, 100 MHz): δ 175.1, 163.1, 156.7, 142.8, 137.1, 136.4, 134.2, 129.8, 128.9, 128.5, 128.3, 127.7, 127.7, 127.7, 127.5, 125.8, 125.4, 83.1, 81.5, 47.1, 43.7, 28.3, 21.1;

HRMS (ESI): $C_{34}H_{39}N_3O_6Na$ for $[M+Na]^+$, calculated 608.2737, found 608.2736;

The enantiomeric excess was determined to be 91.50% by HPLC. [IA column, 230 nm, n-hexane:IPA = 90:10, 0.5 mL/min]: 22.6 min (major), 34.1 min (minor);

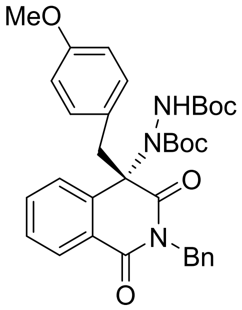

di-tert-butyl(S)-1-(2-benzyl-4-(4-methoxybenzyl)-1,3-dioxo-1,2,3,4-tetrahydroisoquinolin-4-yl)hydrazine-1,2-dicarboxylate (6g):

White solid, 16.2 mg, 90% yield. mp: 157.0-158.8 ℃;

Optical rotation: [α]25.5 D= -2.82 (c 1.4, $CHCl_2$);

^{1}H NMR ($CDCl_3$, 400 MHz): δ 8.44 (d, *J* = 7.3 Hz, 1H), 7.90 – 7.85 (m, 1H), 7.71 (t, *J* = 7.3 Hz, 1H), 7.38 (dd, *J* = 18.8, 11.1 Hz, 3H), 7.25 – 7.20 (m, 3H), 6.88 (s, 1H), 6.34 (d, *J* = 8.6 Hz, 2H), 6.12 (d, *J* = 8.6 Hz, 2H), 4.93 (d, *J* = 13.5 Hz, 1H), 4.79 (d, *J* = 13.5 Hz, 1H), 3.64 (s, 3H), 3.02 (d, *J* = 12.4 Hz, 1H), 2.82 (dd, *J* = 38.9, 15.6 Hz, 1H), 1.54 (s, 18H);

^{13}C NMR ($CDCl_3$, 100 MHz): δ 175.1, 169.8, 163.1, 158.8, 142.8, 136.4, 134.2, 131.0, 129.5, 128.2, 127.7, 127.6, 127.5, 125.8, 125.3, 123.8, 113.2, 83.1, 81.5, 55.0, 43.7, 30.5, 28.3;

HRMS (ESI): $C_{34}H_{39}N_3O_7Na$ for $[M+Na]^+$, calculated 624.2686, found 624.2686；

The enantiomeric excess was determined to be 97.16% by HPLC. [OD-H column, 230 nm, n-hexane:IPA = 99:1, 0.5 mL/min]: 38.5 min (major), 55.2 min (minor);

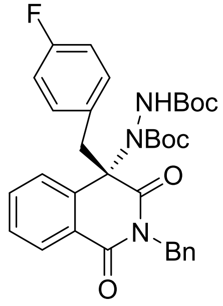

di-tert-butyl(S)-1-(2-benzyl-4-(4-fluorobenzyl)-1,3-dioxo-1,2,3,4-tetrahydroisoquinolin-4-yl)hydrazine-1,2-dicarboxylate (6h):

White solid, 17.0 mg, 96% yield. mp: 93.4-97.1 ℃;

Optical rotation: [α]25.4 D= -10.00 (c 1.6, $CHCl_2$);

^{1}H NMR ($CDCl_3$, 400 MHz): δ 8.43 (d, *J* = 7.0 Hz, 1H), 7.87 (d, *J* = 7.1 Hz, 1H), 7.70 (t, *J* = 7.2 Hz, 1H), 7.38 (dd, *J* = 15.6, 7.8 Hz, 3H), 7.21 (dt, *J* = 7.6, 3.4 Hz, 3H), 6.84 (s, 1H), 6.46 (t, *J* =

8.6 Hz, 2H), 6.13 (dd, J = 8.4, 5.4 Hz, 2H), 4.93 (d, J = 13.5 Hz, 1H), 4.78 (d, J = 13.5 Hz, 1H), 3.59 (d, J = 12.3 Hz, 1H), 3.02 (d, J = 12.4 Hz, 1H), 1.52 (s, 18H);

^{13}C NMR ($CDCl_3$, 100 MHz): δ 174.9, 163.3, 163.0, 160.8, 142.5, 136.2, 134.4, 131.5 (d, J = 8.2 Hz, 1H), 128.3, 127.9, 127.7, 125.8, 125.3, 114.9, 114.7, 83.2, 81.7, 46.6, 43.7, 28.3;

HRMS (ESI): $C_{33}H_{36}N_3O_6FNa$ for $[M+Na]^+$, calculated 612.2486, found 612.2487;

The enantiomeric excess was determined to be 95.67% by HPLC. [OD-H column, 230 nm, n-hexane:IPA = 99:1, 0.5 mL/min]: 23.1 min (major), 32.4 min (minor);

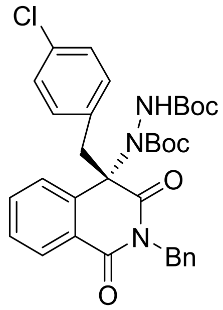

di-tert-butyl(S)-1-(2-benzyl-4-(4-chlorobenzyl)-1,3-dioxo-1,2,3,4-tetrahydroisoquinolin-4-yl)hydrazine-1,2-dicarboxylate(6i):

White solid, 18.0 mg, 99% yield. mp: 90.4-91.7 ℃;

Optical rotation: [α]25.8 D= 1.93 (c 1.7, $CHCl_2$);

^{1}H NMR ($CDCl_3$, 400 MHz): δ 8.43 (d, J = 7.2 Hz, 1H), 7.98 – 7.79 (m, 1H), 7.70 (t, J = 7.2 Hz, 1H), 7.49 – 7.30 (m, 3H), 7.25 – 7.18 (m, 3H), 6.87 (s, 1H), 6.72 (d, J = 8.3 Hz, 2H), 6.08 (d, J = 8.4 Hz, 2H), 4.86 (dd, J = 63.1, 13.5 Hz, 2H), 3.64 (dt, J = 36.6, 9.6 Hz, 1H), 2.99 (t, J = 13.9 Hz, 1H), 1.52 (s, 18H);

^{13}C NMR ($CDCl_3$, 100 MHz): δ 174.7, 163.0, 156.7, 142.4, 136.2, 134.4, 133.4, 131.2, 130.6, 128.3, 127.9, 127.9, 127.6, 125.8, 125.2, 83.2, 81.7, 46.7, 43.6, 28.3;

HRMS (ESI): $C_{33}H_{36}N_3O_6ClNa$ for $[M+Na]^+$, calculated 628.2190, found 628.2191;

The enantiomeric excess was determined to be 99.46% by HPLC. [OD-H column, 230 nm, n-hexane:IPA = 99:1, 0.3 mL/min]: 54.6 min (major), 68.4 min (minor);

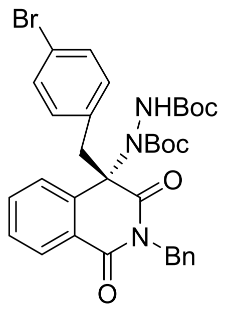

di-tert-butyl(S)-1-(2-benzyl-4-(4-bromobenzyl)-1,3-dioxo-1,2,3,4-tetrahydroisoquinolin-4-yl)hydrazine-1,2-dicarboxylate (6j):

White solid, 19.3 mg, 99% yield. mp: 141.0-142.3 ℃;

Optical rotation: [α]25.9 D= 3.45 (c 2.5, $CHCl_2$);

^{1}H NMR ($CDCl_3$, 400 MHz): δ 8.43 (d, *J* = 7.2 Hz, 1H), 7.89 (d, *J* = 7.2 Hz, 1H), 7.70 (t, *J* = 7.2 Hz, 1H), 7.45 – 7.34 (m, 3H), 7.26 – 7.20 (m, 3H), 6.94 – 6.78 (m, 3H), 6.02 (d, *J* = 8.3 Hz, 2H), 4.94 (d, *J* = 13.5 Hz, 1H), 4.78 (d, *J* = 13.5 Hz, 1H), 3.56 (d, *J* = 12.2 Hz, 1H), 3.00 (d, *J* = 12.3 Hz, 1H), 1.52 (s, 18H);

^{13}C NMR ($CDCl_3$, 100 MHz): δ 174.7, 163.0, 156.7, 142.3, 136.2, 134.4, 133.4, 131.5, 131.1, 130.9, 130.1, 128.3, 127.9, 127.6, 125.8, 125.3, 121.6, 83.3, 81.7, 46.7, 43.6, 28.3;

HRMS (ESI): $C_{33}H_{36}N_3O_6BrNa$ for $[M+Na]^+$, calculated 672.1685, found 672.1687;

The enantiomeric excess was determined to be 98.96% by HPLC. [OD-H column, 230 nm, n-hexane:IPA = 99:1, 0.3 mL/min]: 57.6 min (major), 74.2 min (minor);

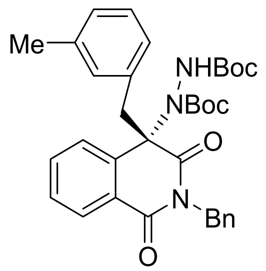

di-tert-butyl(S)-1-(2-benzyl-4-(3-methylbenzyl)-1,3-dioxo-1,2,3,4-tetrahydroisoquinolin-4-yl)hydrazine-1,2-dicarboxylate (6k):

White solid, 17.4 mg, 99% yield. mp: 87.9-90.3 ℃;

Optical rotation: [α]26.0 D= -12.17 (c 0.6, $CHCl_2$);

^{1}H NMR ($CDCl_3$, 400 MHz): δ 8.48 (d, *J* = 7.2 Hz, 1H), 7.86 (d, *J* = 7.3 Hz, 1H), 7.73 (t, *J* = 7.2 Hz, 1H), 7.52 – 7.33 (m, 3H), 7.26 – 7.16 (m, 3H), 6.90 (s, 2H), 6.78 (t, *J* = 7.6 Hz, 1H), 6.12 (d, *J* = 7.6 Hz, 1H), 6.07 (s, 1H), 4.91 (d, *J* = 13.5 Hz, 1H), 4.75 (d, *J* = 13.5 Hz, 1H), 3.64 (d, *J* =

12.0 Hz, 1H), 3.07 (d, J = 12.1 Hz, 1H), 2.04 (s, 3H), 1.56 (s, 18H);

^{13}C NMR ($CDCl_3$, 100 MHz): δ 175.0, 163.0, 156.7, 142.8, 137.3, 136.4, 134.2, 132.1, 130.8, 128.3, 128.2, 127.6, 127.6, 127.5, 127.0, 125.7, 125.4, 124.0, 83.1, 81.5, 47.4, 43.7, 28.3, 21.0;

HRMS (ESI): $C_{34}H_{39}N_3O_6Na$ for $[M+Na]^+$, calculated 608.2737, found 608.2736;

The enantiomeric excess was determined to be 75.98% by HPLC. [IC column, 230 nm, n-hexane:IPA = 97:3, 0.1 mL/min]: 98.6 min (major), 112.8 min (minor);

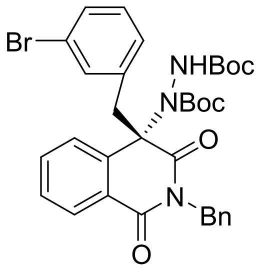

di-tert-butyl(S)-1-(2-benzyl-4-(3-bromobenzyl)-1,3-dioxo-1,2,3,4-tetrahydroisoquinolin-4-yl)hydrazine-1,2-dicarboxylate (6l):

White solid, 19.3 mg, 99% yield. mp: 96.7-97.7 ℃;

Optical rotation: [α]25.5 D= -15.53 (c 1.8, $CHCl_2$);

^{1}H NMR ($CDCl_3$, 400 MHz): δ 8.46 (d, J = 6.9 Hz, 1H), 7.90 (d, J = 7.8 Hz, 1H), 7.75 (t, J = 7.6 Hz, 1H), 7.42 (t, J = 7.5 Hz, 3H), 7.26 – 7.19 (m, 4H), 6.87 (s, 1H), 6.70 (t, J = 7.8 Hz, 1H), 6.45 (s, 1H), 6.19 (d, J = 7.6 Hz, 1H), 4.95 (d, J = 13.6 Hz, 1H), 4.82 (d, J = 13.6 Hz, 1H), 3.63 (d, J = 11.9 Hz, 1H), 3.05 (d, J = 12.2 Hz, 1H), 1.56 (s, 18H);

^{13}C NMR ($CDCl_3$, 100 MHz): δ 174.7, 162.8, 156.7, 142.3, 136.3, 134.6, 134.4, 133.1, 130.6, 129.2, 128.5, 128.3, 128.0, 127.8, 127.7, 127.6, 125.6, 125.2, 121.7, 83.3, 81.7, 47.1, 43.8, 28.3;

HRMS (ESI): $C_{33}H_{36}N_3O_6BrNa$ for $[M+Na]^+$, calculated 672.1685, found 672.1688;

The enantiomeric excess was determined to be 88.55% by HPLC. [IC column, 230 nm, n-hexane:IPA = 97:3, 0.1 mL/min]: 101.3 min (major), 116.3 min (minor);

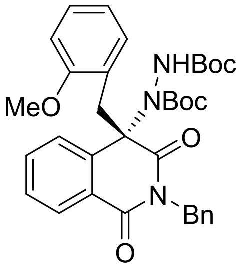

di-tert-butyl(S)-1-(2-benzyl-4-(2-methoxybenzyl)-1,3-dioxo-1,2,3,4-tetrahydroisoquinolin-4-

yl)hydrazine-1,2-dicarboxylate (6m):

White solid, 17.8 mg, 99% yield. mp: 93.9-95.0 ℃;

Optical rotation: [α]25.2 D= -25.95 (c 1.5, $CHCl_2$);

^{1}H NMR ($CDCl_3$, 400 MHz): δ 8.37 (d, *J* = 7.0 Hz, 1H), 7.71 (d, *J* = 7.8 Hz, 1H), 7.59 (t, *J* = 7.5 Hz, 1H), 7.27 (dd, *J* = 33.1, 25.7 Hz, 3H), 7.18 – 7.10 (m, 3H), 7.04 – 6.99 (m, 1H), 6.87 (s, 1H), 6.52 (d, *J* = 8.2 Hz, 1H), 6.41 (t, *J* = 7.4 Hz, 1H), 6.08 (d, *J* = 6.9 Hz, 1H), 4.87 (d, *J* = 11.4 Hz, 1H), 4.72 (d, *J* = 13.5 Hz, 1H), 3.70 (d, *J* = 11.9 Hz, 1H), 3.38 (s, 3H), 3.07 (d, *J* = 12.2 Hz, 1H), 1.49 (s, 18H);

^{13}C NMR ($CDCl_3$, 100 MHz): δ 175.0, 163.2, 157.6, 156.8, 142.9, 136.6, 133.6, 132.1, 130.2, 129.2, 129.1, 128.3, 128.2, 127.3, 127.3, 126.1, 124.8, 120.7, 119.7, 109.6, 82.9, 81.4, 54.8, 44.0, 29.7, 28.3;

HRMS (ESI): $C_{34}H_{39}N_3O_7Na$ for $[M+Na]^+$, calculated 624.2686, found 624.2687;

The enantiomeric excess was determined to be 92.88% by HPLC. [OD-H column, 230 nm, n-hexane:IPA = 97:3, 0.5 mL/min]: 18.6 min (major), 33.9 min (minor);

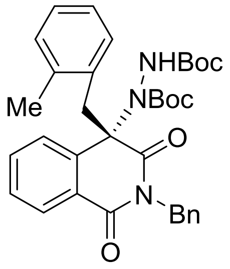

di-tert-butyl(S)-1-(2-benzyl-4-(2-methylbenzyl)-1,3-dioxo-1,2,3,4-tetrahydroisoquinolin-4-yl)hydrazine-1,2-dicarboxylate (6n):

White solid, 16.1mg, 92% yield. mp: 180.6-181.8 ℃;

Optical rotation: [α]25.5 D= -36.42 (c 1.8, $CHCl_2$);

^{1}H NMR ($CDCl_3$, 400 MHz): δ 8.56 (d, *J* = 7.1 Hz, 1H), 7.88 (d, *J* = 7.8 Hz, 1H), 7.69 (t, *J* = 7.5 Hz, 1H), 7.40 (t, *J* = 7.5 Hz, 3H), 7.24 – 7.15 (m, 3H), 7.01 (t, *J* = 7.4 Hz, 1H), 6.95 (s, 1H), 6.87 (d, *J* = 7.6 Hz, 1H), 6.75 (t, *J* = 7.5 Hz, 1H), 6.23 (d, *J* = 7.2 Hz, 1H), 4.93 (d, *J* = 12.9 Hz, 1H), 4.75 (d, *J* = 13.6 Hz, 1H), 3.83 – 3.70 (m, 1H), 3.16 (d, *J* = 12.5 Hz, 1H), 1.59 (d, *J* = 15.4 Hz, 21H);

^{13}C NMR ($CDCl_3$, 100 MHz): δ 175.1, 171.1, 163.0, 142.6, 138.0, 136.3, 134.1, 133.2, 133.2, 131.1, 130.4, 130.3, 128.2, 127.7, 127.6, 127.5, 126.3, 125.2, 125.0, 83.1, 81.5, 43.9, 28.3, 18.6, 14.2;

HRMS (ESI): $C_{34}H_{39}N_3O_6Na$ for $[M+Na]^+$, calculated 608.2737, found 608.2736;

The enantiomeric excess was determined to be 92.67% by HPLC. [IA column, 230 nm, n-hexane:IPA = 95:5, 0.5 mL/min]: 39.3 min (major), 44.1 min (minor);

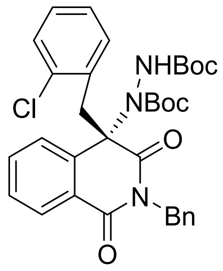

di-tert-butyl(S)-1-(2-benzyl-4-(2-chlorobenzyl)-1,3-dioxo-1,2,3,4-tetrahydroisoquinolin-4-yl)hydrazine-1,2-dicarboxylate (6o):

White solid, 11.5 mg, 95% yield. mp: 186.6-187.6 ℃;

Optical rotation: [[α]25.4 D= -31.43 (c 1.4, $CHCl_2$);

^{1}H NMR ($CDCl_3$, 400 MHz): δ 8.40 (d, J = 7.0 Hz, 1H), 7.73 (d, J = 7.3 Hz, 1H), 7.60 (t, J = 7.2 Hz, 1H), 7.29 (d, J = 7.9 Hz, 2H), 7.11 (ddd, J = 27.1, 15.6, 8.1 Hz, 5H), 6.94 (td, J = 7.9, 1.5 Hz, 1H), 6.87 (s, 1H), 6.62 (t, J = 7.4 Hz, 1H), 5.98 (d, J = 7.5 Hz, 1H), 4.96 (d, J = 13.4 Hz, 1H), 4.77 (d, J = 13.7 Hz, 1H), 3.90 (d, J = 12.6 Hz, 1H), 3.12 (d, J = 12.7 Hz, 1H), 1.48 (s, 18H);

^{13}C NMR ($CDCl_3$, 100 MHz): δ 174.4, 163.0, 156.8, 136.5, 135.6, 134.1, 132.5, 130.5, 129.3, 129.0, 128.2, 127.8, 126.1, 125.9, 125.1, 83.2, 81.6, 44.4, 28.3, 27.2;

HRMS (ESI): $C_{33}H_{36}N_3O_6ClNa$ for $[M+Na]^+$, calculated 628.2190, found 628.2191;

The enantiomeric excess was determined to be 98.04% by HPLC. [OD-H column, 230 nm, n-hexane:IPA = 97:3, 0.5 mL/min]: 14.1 min (major), 18.7 min (minor);

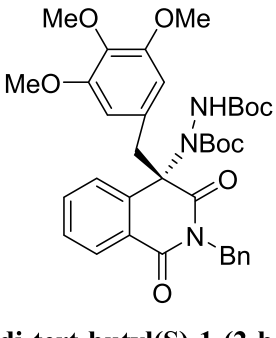

di-tert-butyl(S)-1-(2-benzyl-1,3-dioxo-4-(3,4,5-trimethoxybenzyl)-1,2,3,4-tetrahydroisoquinolin-4-yl)hydrazine-1,2-dicarboxylate (6p):

Yellow solid, 19.6 mg, 99% yield. mp: 103.7-105.3 ℃;

Optical rotation: [α]26.0 D= -15.32 (c 1.9, $CHCl_2$);

^{1}H NMR ($CDCl_3$, 400 MHz): δ 8.50 (d, *J* = 7.6 Hz, 1H), 7.96 – 7.89 (m, 1H), 7.74 (dd, *J* = 11.1, 4.1 Hz, 1H), 7.45 – 7.12 (m, 7H), 6.90 (s, 1H), 5.56 (s, 2H), 4.91 (d, *J* = 13.4 Hz, 1H), 4.79 (d, *J* = 13.5 Hz, 1H), 3.74 (s, 3H), 3.64 (d, *J* = 12.2 Hz, 1H), 3.48 (s, 6H), 3.07 (d, *J* = 12.3 Hz, 1H), 1.56 (s, 18H);

^{13}C NMR ($CDCl_3$, 100 MHz): δ 174.9, 162.7, 156.7, 152.4, 142.9, 137.6, 136.2, 134.2, 128.3, 127.8, 127.6, 127.6, 127.5, 125.9, 125.7, 107.2, 83.2, 81.6, 60.9, 55.8, 47.5, 43.8, 28.3;

HRMS (ESI): $C_{36}H_{44}N_3O_9$ for $[M+H]^+$, calculated 662.3078, found 662.3077；

The enantiomeric excess was determined to be 86.97% by HPLC. [IA column, 230 nm, n-hexane:IPA = 80:20, 1.0 mL/min]: 11.9min (major), 25.5 min (minor);

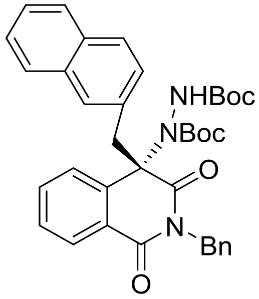

di-tert-butyl(S)-1-(2-benzyl-4-(naphthalen-2-ylmethyl)-1,3-dioxo-1,2,3,4-tetrahydroisoquinolin-4-yl)hydrazine-1,2-dicarboxylate (6q):

Yellow solid, 18.4 mg, 99% yield. mp: 104.8-105.2 ℃;

Optical rotation: [α]25.7 D= 11.76 (c 0.7, $CHCl_2$);

^{1}H NMR ($CDCl_3$, 400 MHz): δ 8.56 (d, *J* = 7.2 Hz, 1H), 7.78 (t, *J* = 8.6 Hz, 2H), 7.67 (d, *J* = 7.9 Hz, 1H), 7.47 – 7.29 (m, 7H), 7.23 – 7.16 (m, 3H), 6.98 (s, 1H), 6.80 (s, 1H), 6.36 (dd, *J* = 8.4, 1.2 Hz, 1H), 4.93 (d, *J* = 13.6 Hz, 1H), 4.66 (d, *J* = 13.6 Hz, 1H), 3.85 (d, *J* = 12.1 Hz, 1H), 3.28 (d, *J* = 12.2 Hz, 1H), 1.58 (s, 18H);

^{13}C NMR ($CDCl_3$, 100 MHz): δ 175.0, 171.1, 162.8, 142.7, 136.3, 134.3, 132.7, 132.3, 129.7, 129.3, 128.2, 127.7, 127.5, 127.5, 127.3, 126.0, 126.0, 125.9, 125.2, 83.2, 81.6, 43.7, 29.7, 28.3;

HRMS (ESI): $C_{37}H_{39}N_3O_6Na$ for $[M+Na]^+$, calculated 644.2737, found 644.2739；

The enantiomeric excess was determined to be >99.00% by HPLC. [IA column, 230 nm, n-hexane:IPA = 97:3, 0.5 mL/min]: 128.6 min (major);

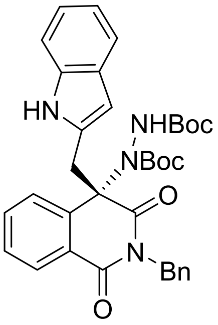

di-tert-butyl(S)-1-(4-((1H-indol-2-yl)methyl)-2-benzyl-1,3-dioxo-1,2,3,4-tetrahydroisoquinolin-4-yl)hydrazine-1,2-dicarboxylate (6r):

Yellow oil, 16.5 mg, 90% yield;

Optical rotation: [α]25.8 D= 3.70 (c 1.1, $CHCl_2$);

^{1}H NMR ($CDCl_3$, 400 MHz): δ 8.39 (d, *J* = 7.6 Hz, 1H), 7.94 (d, *J* = 7.8 Hz, 1H), 7.73 (t, *J* = 7.6 Hz, 1H), 7.42 (dd, *J* = 16.2, 7.8 Hz, 3H), 7.27 (d, *J* = 9.4 Hz, 5H), 6.97 (dq, *J* = 13.6, 7.7 Hz, 5H), 5.04 (d, *J* = 13.6 Hz, 1H), 4.87 (d, *J* = 13.6 Hz, 1H), 3.74 – 3.68 (m, 1H), 3.34 (d, *J* = 13.7 Hz, 1H), 1.45 (s, 18H);

^{13}C NMR ($CDCl_3$, 100 MHz): δ 175.0, 162.9, 156.9, 136.3, 135.9, 134.3, 129.6, 129.1, 128.4, 128.4, 128.0, 127.6, 125.3, 124.9, 121.9, 120.2, 119.7, 110.4, 104.1, 83.4, 81.9, 43.8, 29.7, 28.3;

HRMS (ESI): $C_{35}H_{39}N_4O_6$ for $[M+H]^+$, calculated 611.2870, found 611.2871;

The enantiomeric excess was determined to be 81.97% by HPLC. [OD-H column, 230 nm, n-hexane:IPA = 97:3, 0.3 mL/min]: 71.4 min (major), 89.4 min (minor);

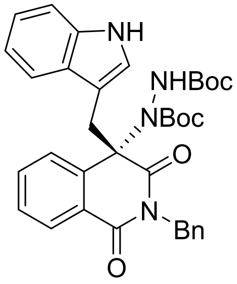

di-tert-butyl(S)-1-(4-((1H-indol-3-yl)methyl)-2-benzyl-1,3-dioxo-1,2,3,4-tetrahydroisoquinolin-4-yl)hydrazine-1,2-dicarboxylate (6s):

Yellow oil, 18.1 mg, 99% yield;

Optical rotation: [α]25.8 D= 35.76 (c 0.9, $CHCl_2$);

^{1}H NMR ($CDCl_3$, 400 MHz): δ 8.47 (d, *J* = 7.3 Hz, 1H), 7.79 (s, 1H), 7.72 (d, *J* = 7.8 Hz, 1H), 7.65 (t, *J* = 7.5 Hz, 1H), 7.29 (d, *J* = 7.5 Hz, 1H), 7.19 (s, 1H), 7.16 – 7.07 (m, 5H), 7.01 (t, *J* = 7.5 Hz, 2H), 6.94 (s, 1H), 6.88 (t, *J* = 7.4 Hz, 1H), 5.72 (s, 1H), 4.57 (d, *J* = 13.7 Hz, 1H), 4.20 (d,

J = 13.7 Hz, 1H), 3.92 (d, *J* = 13.0 Hz, 1H), 3.16 (d, *J* = 13.2 Hz, 1H), 1.51 (s, 18H);

^{13}C NMR ($CDCl_3$, 100 MHz): δ 171.2, 163.2, 156.7, 143.5, 140.8, 136.6, 135.3, 134.1, 130.1, 130.1, 129.9, 129.8, 128.1, 127.5, 127.3, 125.6, 124.3, 122.0, 119.6, 117.9, 111.1, 106.2, 83.0, 81.5, 43.7, 37.5, 28.3;

HRMS (ESI): $C_{35}H_{38}N_4O_6Na$ for $[M+Na]^+$, calculated 633.2689, found 633.2689;

The enantiomeric excess was determined to be 99.34% by HPLC. [IA column, 230 nm, n-hexane:IPA = 95:5, 1.0 mL/min]: 35.1 min (major), 47.5 min (minor);

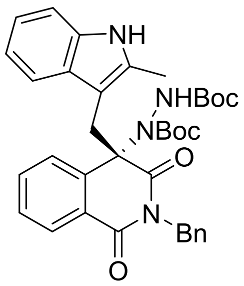

di-tert-butyl(S)-1-(2-benzyl-4-((2-methyl-1H-indol-3-yl)methyl)-1,3-dioxo-1,2,3,4-tetrahydroisoquinolin-4-yl)hydrazine-1,2-dicarboxylate (6t):

Yellow solid, 18.5 mg, 99% yield. mp: 80.3-81.8 ℃;

Optical rotation: [α]25.0 D= -13.33 (c 0.8, $CHCl_2$);

^{1}H NMR ($CDCl_3$, 400 MHz): δ 8.56 (s, 1H), 7.88 (s, 1H), 7.80 (s, 1H), 7.67 (s, 1H), 7.42 (d, *J* = 16.5 Hz, 2H), 7.10 (s, 9H), 4.42 (d, *J* = 11.7 Hz, 1H), 4.13 (dd, *J* = 7.3, 3.0 Hz, 1H), 3.86 (d, *J* = 13.2 Hz, 1H), 3.06 (d, *J* = 11.4 Hz, 1H), 1.35 (d, *J* = 191.9 Hz, 21H);

^{13}C NMR ($CDCl_3$, 100 MHz): δ 175.3, 157.2, 154.2, 143.6, 136.8, 135.4, 134.9, 134.1, 130.1, 128.8, 128.2, 127.6, 126.3, 126.0, 125.4, 121.6, 119.5, 117.8, 110.5, 102.2, 83.1, 81.7, 44.0, 28.4, 27.0, 9.8;

HRMS (ESI): $C_{36}H_{40}N_4O_6Na$ for $[M+Na]^+$, calculated 647.2846, found 647.2846;

The enantiomeric excess was determined to be 95.55% by HPLC. [IC column, 230 nm, n-hexane:IPA = 97:3, 0.5 mL/min]: 67.0 min (major), 84.3 min (minor);

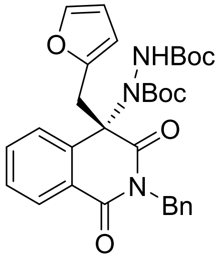

di-tert-butyl(S)-1-(2-benzyl-4-(furan-2-ylmethyl)-1,3-dioxo-1,2,3,4-tetrahydroisoquinolin-4-

yl)hydrazine-1,2-dicarboxylate (6u):

White solid, 16.7 mg, 99% yield. mp: 81.8-82.1 ℃;

Optical rotation: [α]25.7 D= -4.96 (c 1.1, $CHCl_2$);

^{1}H NMR ($CDCl_3$, 400 MHz): δ 8.24 (d, *J* = 7.1 Hz, 1H), 7.87 (d, *J* = 7.8 Hz, 1H), 7.58 (t, *J* = 7.5 Hz, 1H), 7.37 (s, 2H), 7.28 (t, *J* = 7.5 Hz, 1H), 7.18 – 7.10 (m, 3H), 6.77 (d, *J* = 10.9 Hz, 2H), 5.85 (s, 1H), 5.10 (d, *J* = 2.7 Hz, 1H), 4.89 (t, *J* = 9.9 Hz, 2H), 3.61 (dd, *J* = 10.5, 2.9 Hz, 1H), 3.07 (d, *J* = 13.8 Hz, 1H), 2.03 – 0.77 (m, 18H);

^{13}C NMR ($CDCl_3$, 100 MHz): δ 174.7, 163.3, 156.6, 146.8, 142.3, 136.5, 136.0, 134.3, 128.2, 127.8, 127.7, 127.5, 125.4, 124.8, 119.5, 110.3, 109.9, 83.2, 81.6, 44.0, 40.2, 28.2;

HRMS (ESI): $C_{31}H_{35}N_3O_7Na$ for $[M+Na]^+$, calculated 584.2373, found 584.2371；

The enantiomeric excess was determined to be 96.95% by HPLC. [IC column, 230 nm, n-hexane:IPA = 97:3, 0.5 mL/min]: 20.2 min (major), 26.9 min (minor);

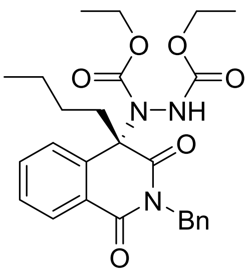

diethyl (S)-1-(2-benzyl-4-butyl-1,3-dioxo-1,2,3,4-tetrahydroisoquinolin-4-yl)hydrazine-1,2-dicarboxylate (6v):

White oil, 13.7 mg, 95% yield;

Optical rotation: [α]20.3 D= -3.98 (c 1.7, $CHCl_3$);

^{1}H NMR ($CDCl_3$, 400 MHz): δ 8.24 – 8.18 (m, 2H), 7.65 (dd, *J* = 11.1, 4.2 Hz, 1H), 7.44 (dd, *J* = 6.8, 4.1 Hz, 3H), 7.29 – 7.27 (m, 1H), 7.26 – 7.19 (m, 2H), 6.95 (s, 1H), 5.25 (d, *J* = 13.8 Hz, 1H), 5.12 (d, *J* = 13.8 Hz, 1H), 4.29 (dddd, *J* = 17.7, 10.6, 7.1, 3.5 Hz, 2H), 3.91 (s, 2H), 2.19 (dd, *J* = 12.1, 8.8 Hz, 1H), 1.82 (td, *J* = 12.6, 4.1 Hz, 1H), 1.55 – 1.12 (m, 5H), 1.11 – 0.70 (m, 5H), 0.58 (t, *J* = 7.3 Hz, 3H);

^{13}C NMR ($CDCl_3$, 100 MHz): δ 175.4, 164.0, 157.2, 155.6, 142.5, 136.6, 134.6, 128.8, 128.3, 127.8, 127.5, 125.4, 124.7, 68.5, 63.1, 62.3, 43.7, 24.9, 22.4, 14.5, 13.5;

HRMS (ESI): $C_{26}H_{32}N_3O_6$ for $[M+H]^+$, calculated 482.2291, found 482.2292;

The enantiomeric excess was determined to be 53.35% by HPLC. [IB column, 230 nm, n-hexane:IPA = 95:5, 0.5 mL/min]: 16.67, min (major), 25.9 min (minor);

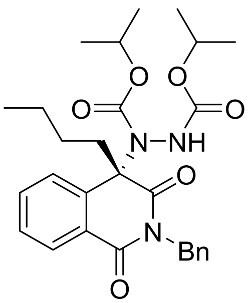

diisopropyl (S)-1-(2-benzyl-4-butyl-1,3-dioxo-1,2,3,4-tetrahydroisoquinolin-4-yl)hydrazine-1,2-dicarboxylate (6w):

White oil, 14.2 mg, 93% yield;

Optical rotation: [α]21.6 D= 3.09 (c 1.3, $CHCl_3$);

^{1}H NMR ($CDCl_3$, 400 MHz): δ 8.22 (t, *J* = 7.8 Hz, 2H), 7.66 (t, *J* = 7.4 Hz, 1H), 7.48 – 7.42 (m, 3H), 7.30 – 7.27 (m, 2H), 7.25 – 7.20 (m, 1H), 6.84 (s, 1H), 5.23 (d, *J* = 13.7 Hz, 1H), 5.13 (d, *J* = 13.8 Hz, 1H), 5.06 (dt, *J* = 12.4, 6.2 Hz, 1H), 4.61 (s, 1H), 1.95 (ddd, *J* = 34.1, 21.4, 10.3 Hz, 2H), 1.44 – 1.25 (m, 8H), 1.19 – 0.70 (m, 8H), 0.59 (t, *J* = 7.3 Hz, 3H);

^{13}C NMR ($CDCl_3$, 100 MHz): δ 175.4, 164.1, 157.1, 155.1, 142.8, 136.6, 134.5, 133.8, 128.3, 128.3, 127.7, 127.5, 125.5, 124.6, 71.2, 70.1, 43.7, 24.9, 22.4, 22.0, 21.9, 13.5;

HRMS (ESI): $C_{28}H_{36}N_3O_6$ for $[M+H]^+$, calculated 510.2604, found 510.2603;

The enantiomeric excess was determined to be 76.84% by HPLC. [IA column, 230 nm, n-hexane:IPA = 85:15, 1.0 mL/min]: 7.3 min (major), 12.4 min (minor);

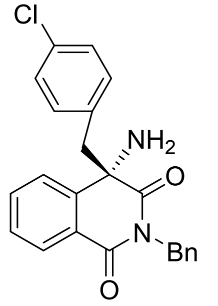

(R)-N-benzyl-1-(4-chlorobenzyl)-3-oxoisoindoline-1-carboxamide (7):

Yellow oil, 10.1 mg, 86% yield;

Optical rotation: [α]25.6 D= -6.27 (c 0.5, $CHCl_2$);

^{1}H NMR ($CDCl_3$, 400 MHz): δ 8.03 (dd, *J* = 7.9, 1.1 Hz, 1H), 7.83 – 7.75 (m, 1H), 7.62 (td, *J* = 7.7, 1.4 Hz, 1H), 7.42 (td, *J* = 7.8, 1.2 Hz, 1H), 7.31 (dd, *J* = 7.7, 1.8 Hz, 2H), 7.25 – 7.18 (m, 4H), 6.85 – 6.70 (m, 2H), 6.31 – 6.09 (m, 2H), 4.97 (d, *J* = 13.7 Hz, 1H), 4.84 (d, *J* = 13.7 Hz, 1H), 3.05 (d, *J* = 12.8 Hz, 1H), 2.91 (d, *J* = 12.8 Hz, 1H);

^{13}C NMR ($CDCl_3$, 100 MHz): δ 176.9, 163.4, 140.9, 136.5, 134.1, 133.2, 132.1, 130.9, 129.5, 128.5, 128.4, 128.1, 127.7, 126.1, 125.5, 61.3, 52.1, 43.8;

HRMS (ESI): $C_{23}H_{20}N_2O_2Cl$ for $[M+H]^+$, calculated 391.1213, found 391.1214;

The enantiomeric excess was determined to be 97.36% by HPLC. [IB column, 230 nm, n-hexane:IPA = 95:5, 1.0 mL/min]: 18.4 min (major), 20.6 min (minor);

7. Crystal data and structure refinement for 6I.

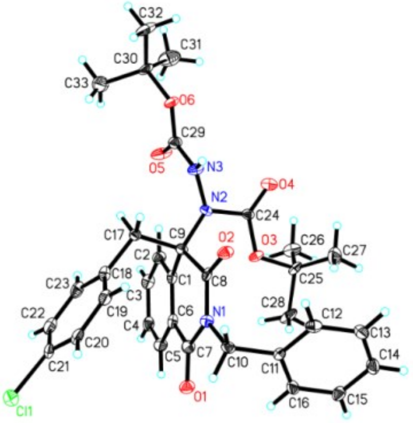

Identification code	6I
Empirical formula	$C_{33}H_{36}ClN_3O_6$
Formula weight	606.10
Temperature/K	293(2)
Crystal system	monoclinic
Space group	$P2_1$
a/Å	10.7532(4)
b/Å	22.6105(9)
c/Å	13.3665(6)
α/°	90
β/°	93.588(4)
γ/°	90
Volume/Å^3	3243.5(2)
Z	4
ρ_{calc}g/cm^3	1.241
μ/mm^{-1}	1.427
F(000)	1280.0
Crystal size/mm^3	0.13 × 0.12 × 0.11
Radiation	CuKα (λ = 1.54184)
2Θ range for data collection/°	6.626 to 147.29
Index ranges	$-13 \leq h \leq 13$, $-27 \leq k \leq 27$, $-1 \leq l \leq 16$
Reflections collected	10317
Independent reflections	10317 [R_{int} = ?, R_{sigma} = 0.0491]
Data/restraints/parameters	10317/8/788
Goodness-of-fit on F^2	1.061
Final R indexes [I>=2σ (I)]	R_1 = 0.0750, wR_2 = 0.2168
Final R indexes [all data]	R_1 = 0.0785, wR_2 = 0.2191
Largest diff. peak/hole / e Å^{-3}	1.09/-0.56
Flack/Hooft parameter	0.028(11)/0.037(9)

Crystal structure determination of [6I]

Crystal Data for $C_{33}H_{36}ClN_3O_6$ (M =606.10 g/mol): monoclinic, space group $P2_1$ (no. 4), a = 10.7532(4) Å, b = 22.6105(9) Å, c = 13.3665(6) Å, β = 93.588(4)°, V = 3243.5(2) Å^3, Z = 4, T = 293(2) K, μ(CuKα) = 1.427 mm^{-1}, $Dcalc$ = 1.241 g/cm^3, 10317 reflections measured (6.626° ≤ 2Θ ≤ 147.29°), 10317 unique (R_{int} = 0.0313, R_{sigma} = 0.0491) which were used in all calculations. The final R_1 was 0.0750 (I > 2σ(I)) and wR_2 was 0.2191 (all data).

8. ^{1}H and ^{13}C NMR Spectra

2-benzyl-4-isopentylisoquinoline-1,3(2H,4H)-dione (4b):

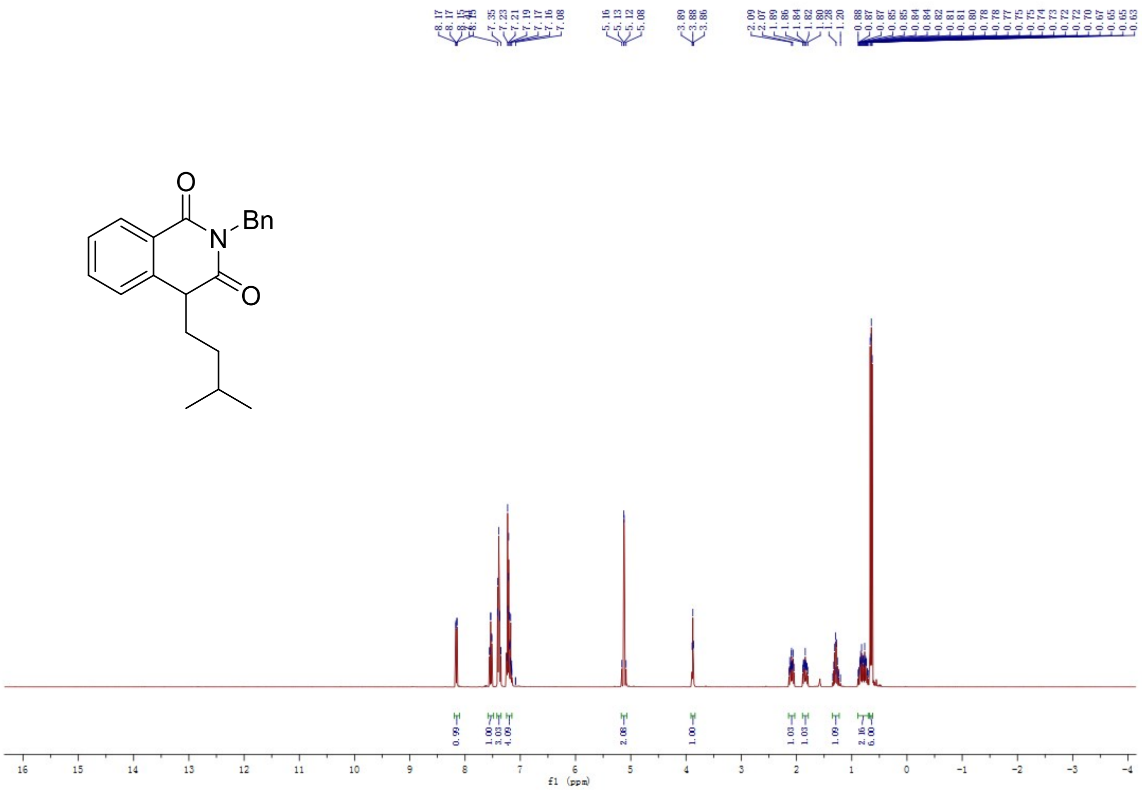

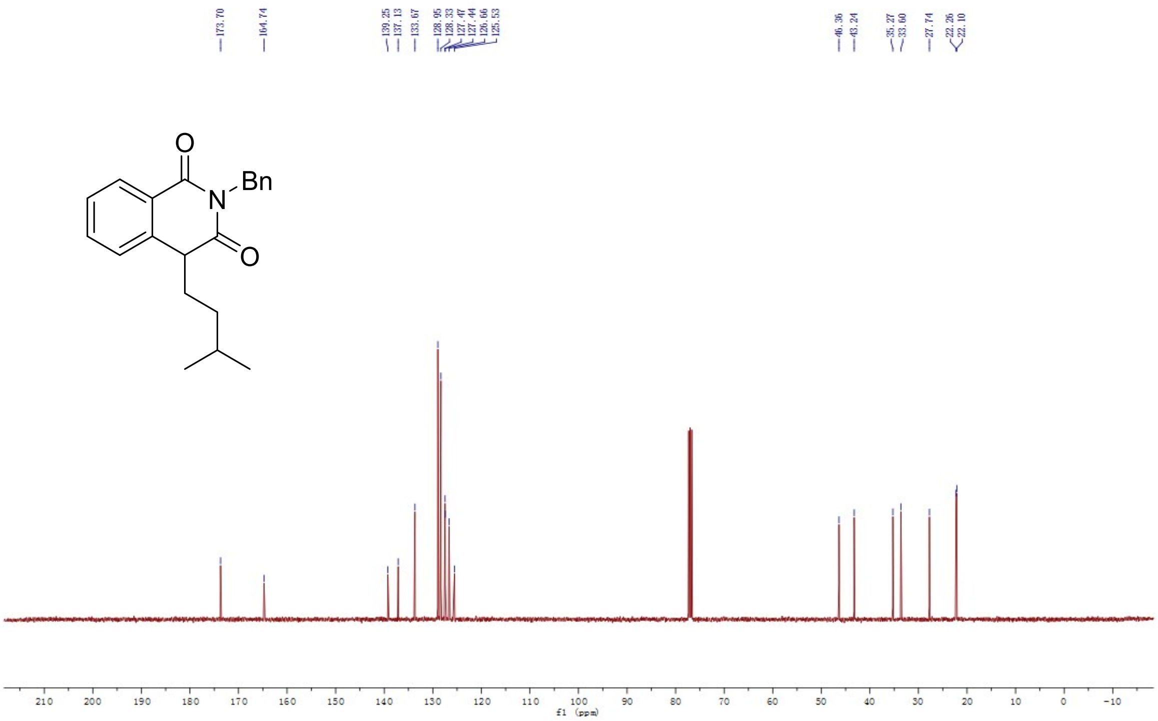

2-benzyl-4-(3-phenylpropyl)isoquinoline-1,3(2H,4H)-dione (4d):

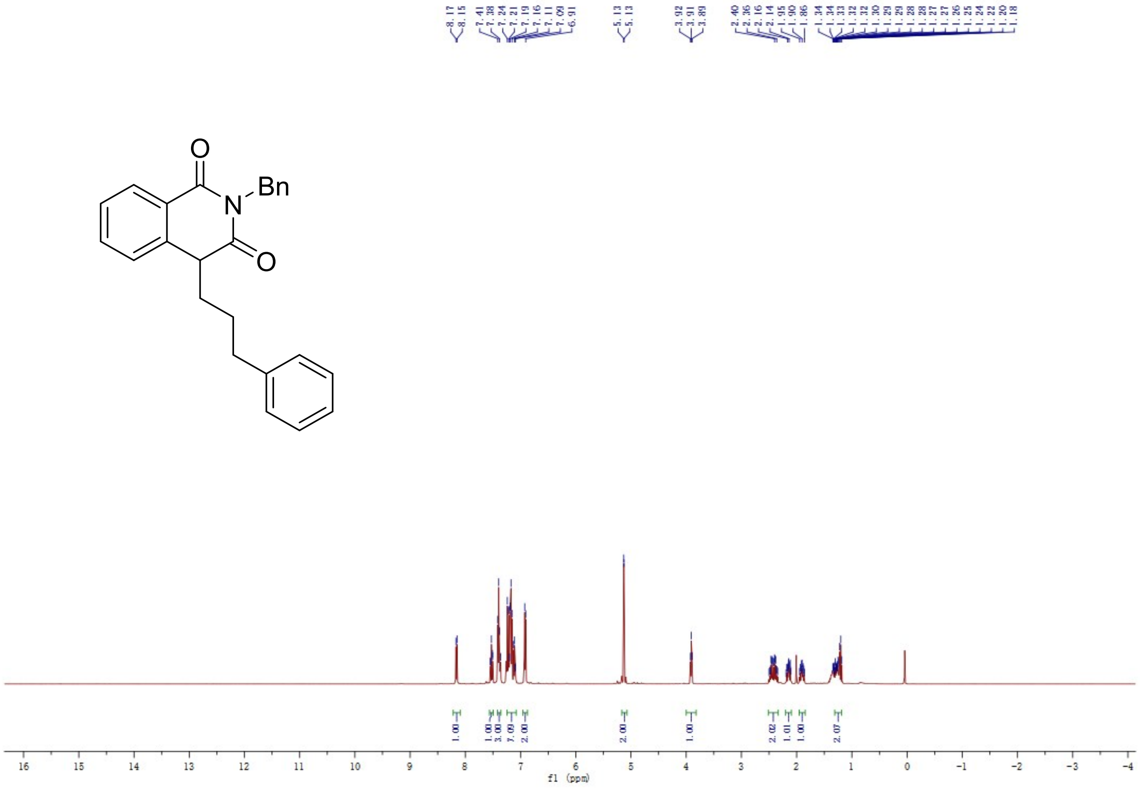

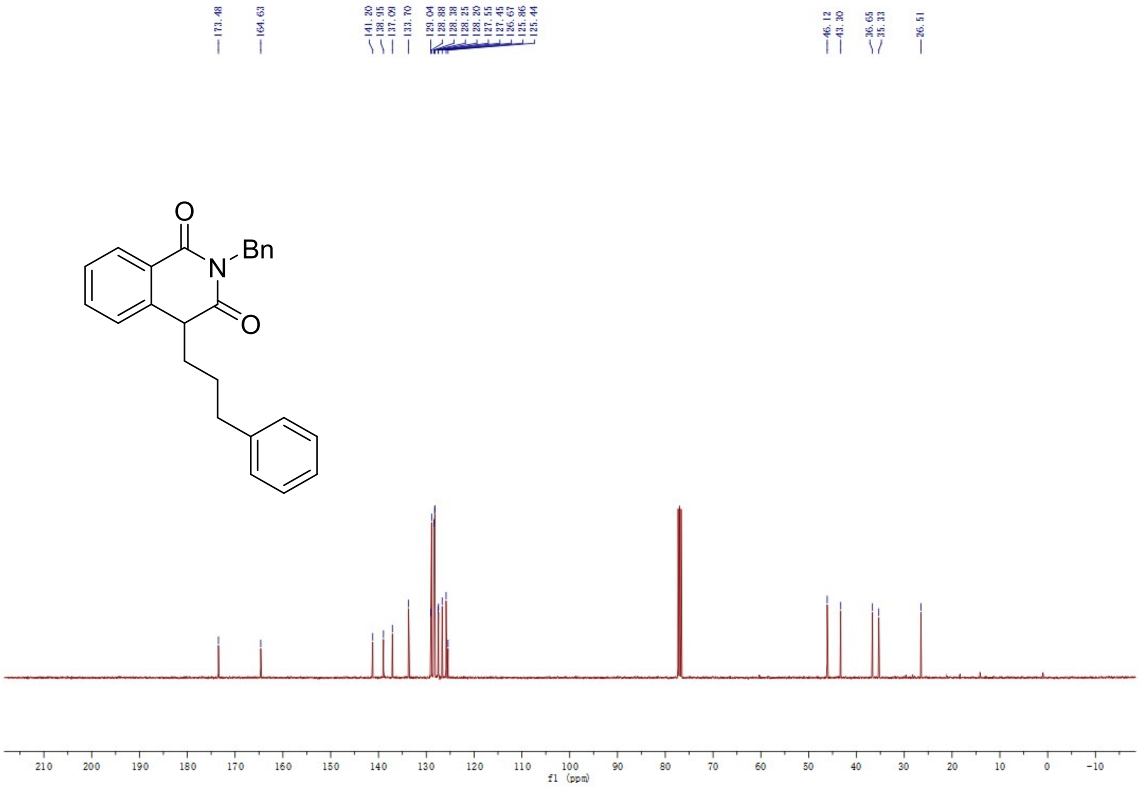

2-benzyl-4-(4-chlorobenzyl)isoquinoline-1,3(2H,4H)-dione (4i):

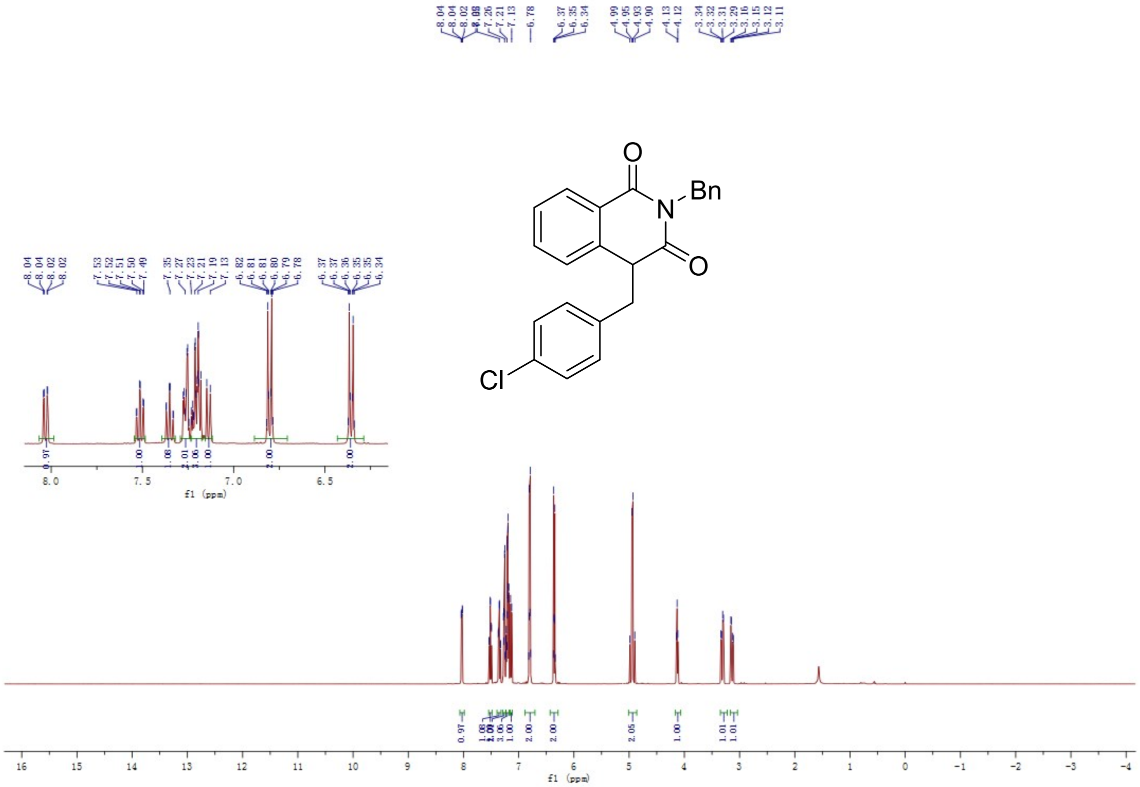

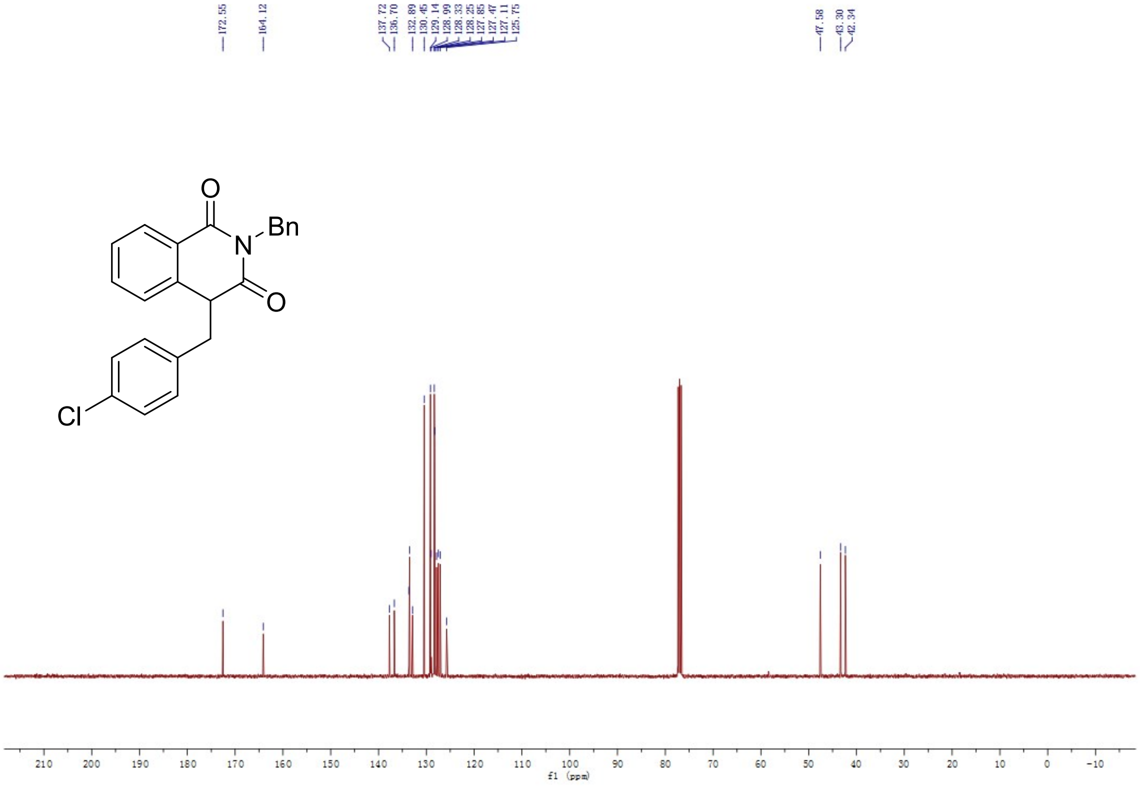

2-benzyl-4-(4-bromobenzyl)isoquinoline-1,3(2H,4H)-dione (4j):

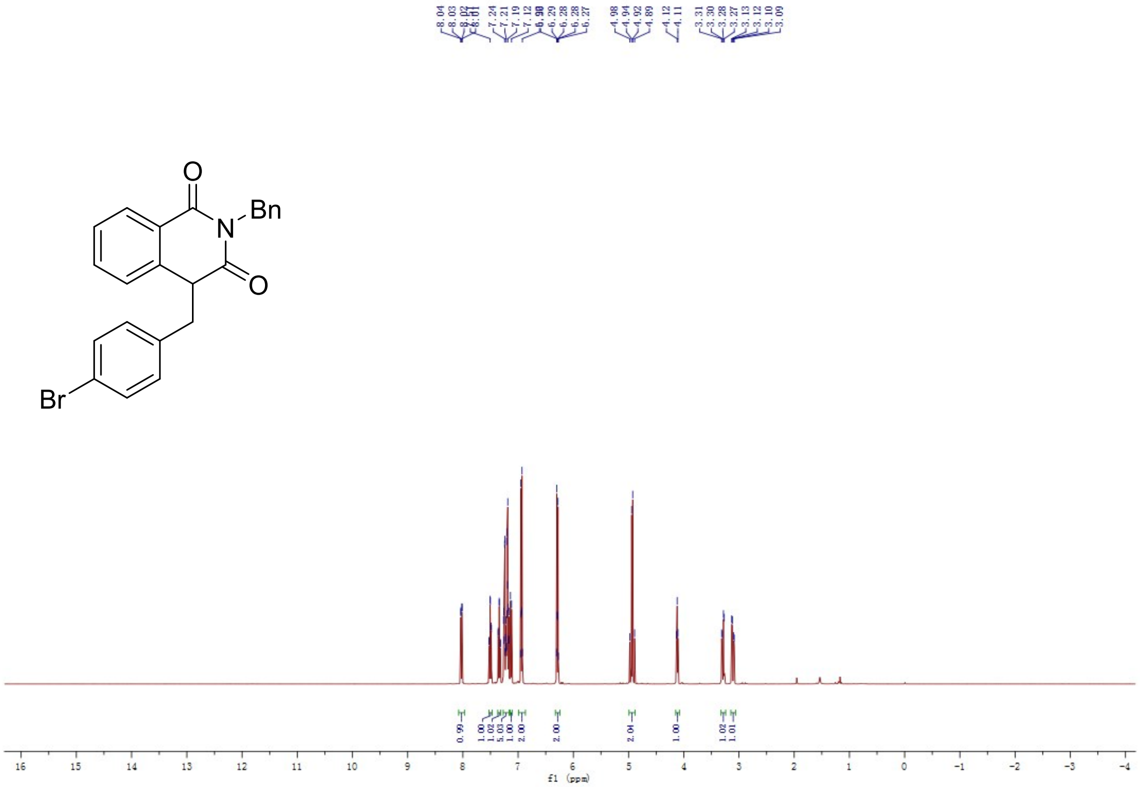

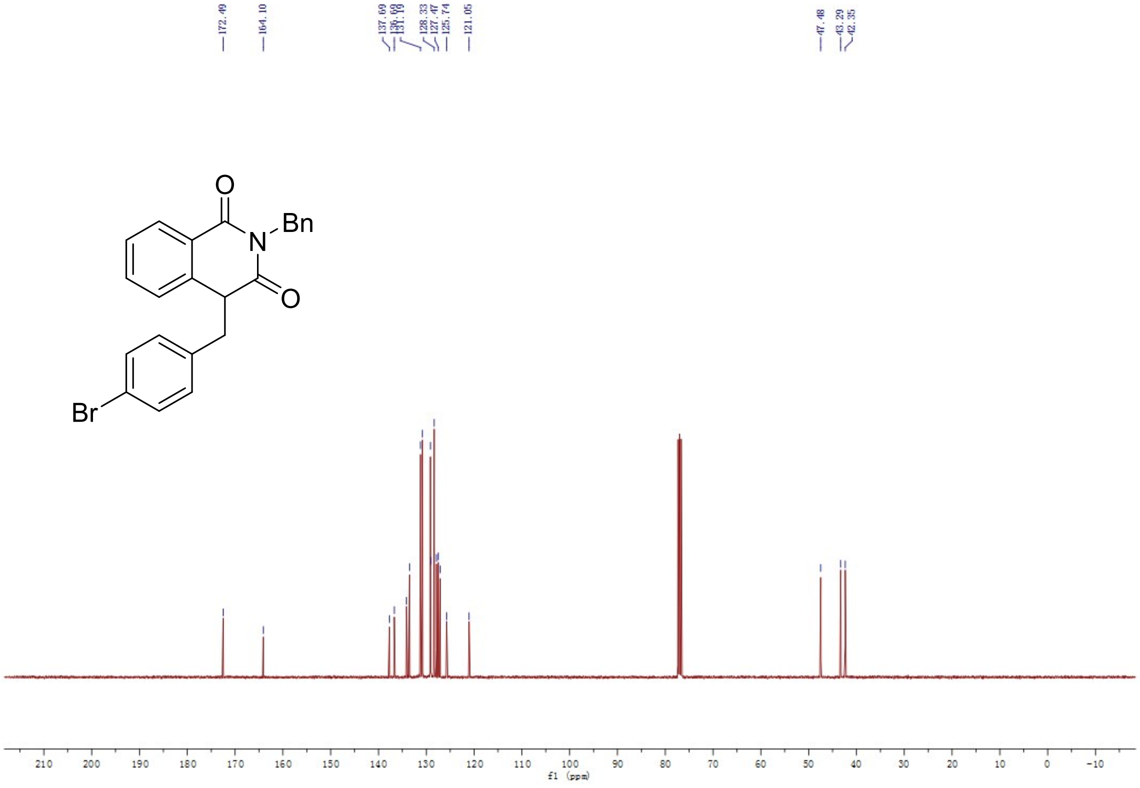

2-benzyl-4-(3-methylbenzyl)isoquinoline-1,3(2H,4H)-dione (4k):

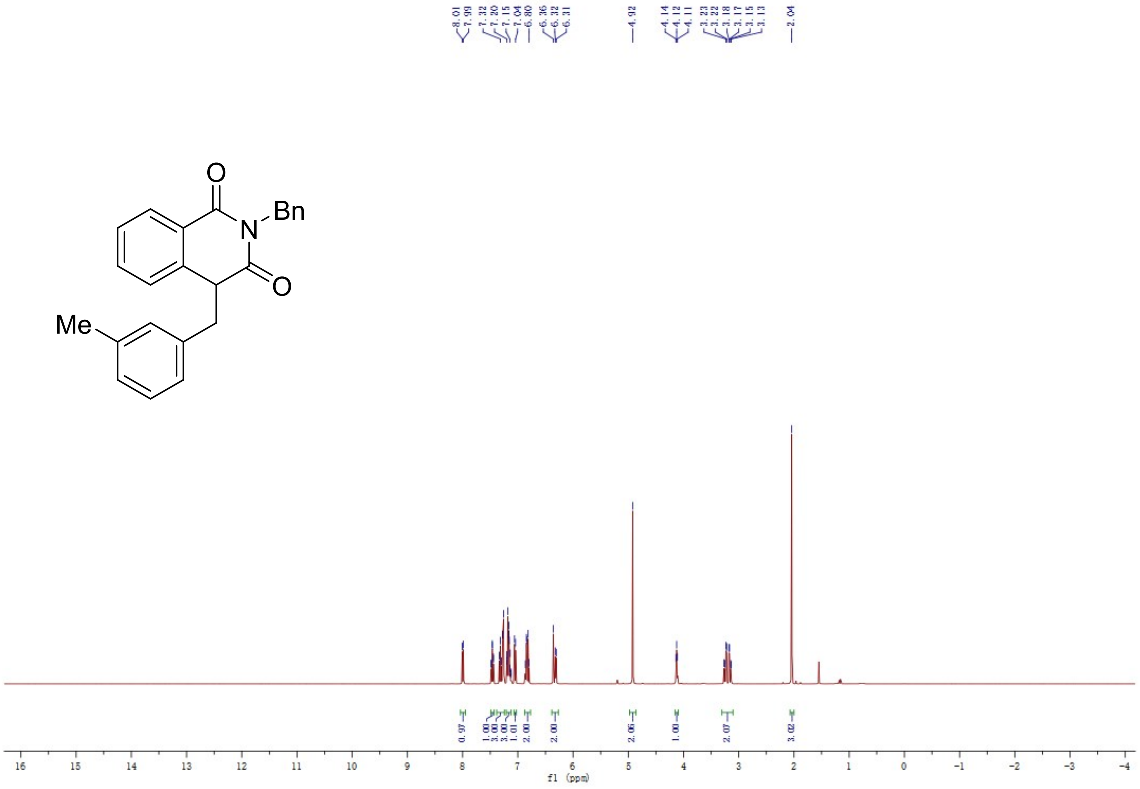

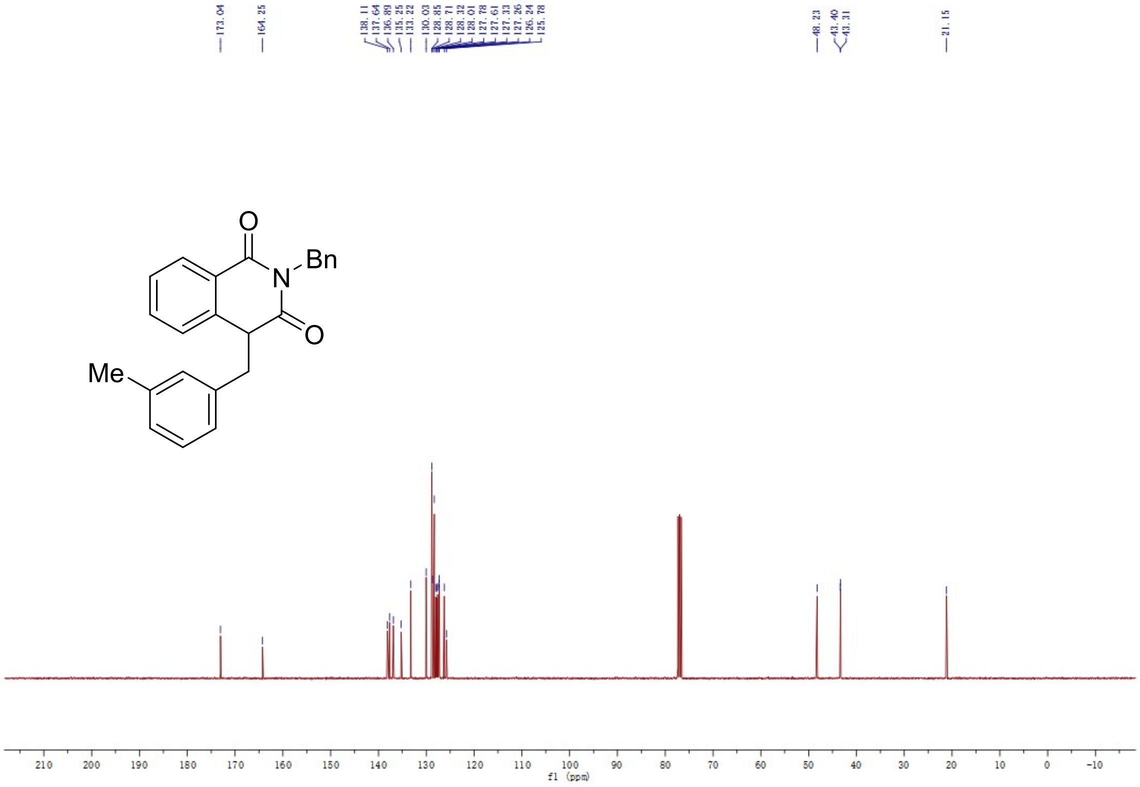

2-benzyl-4-(3-bromobenzyl)isoquinoline-1,3(2H,4H)-dione (4l):

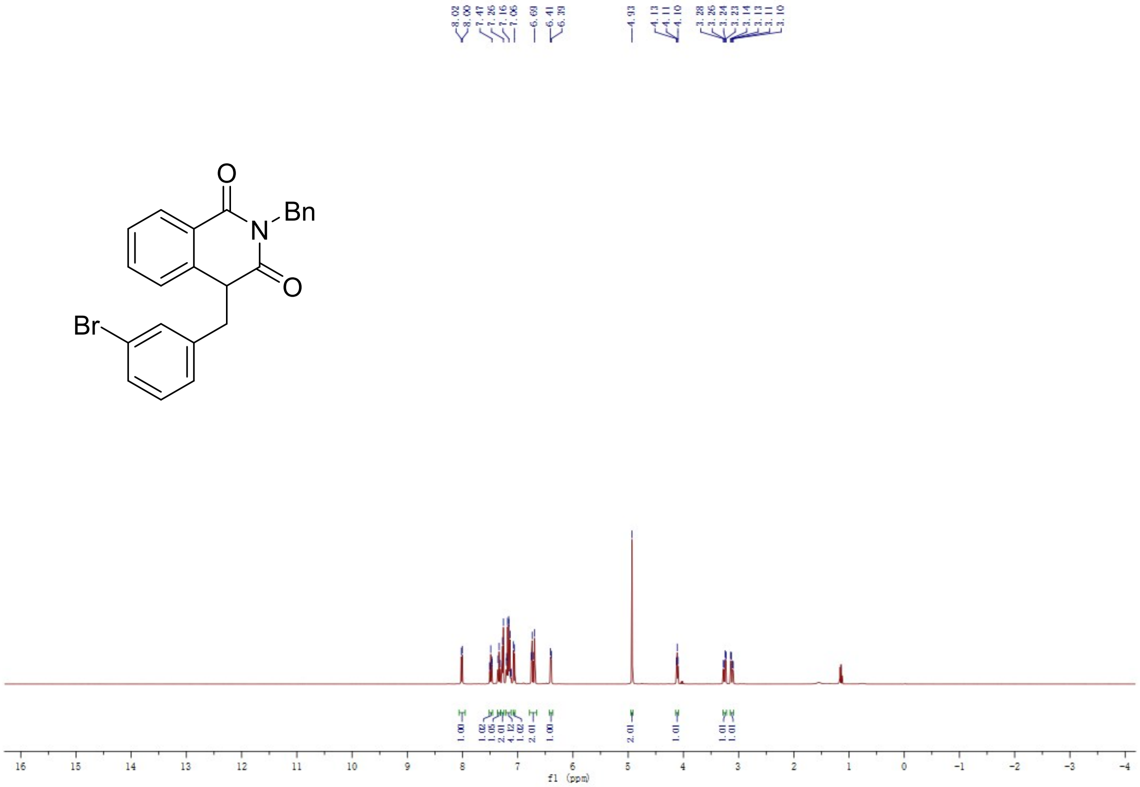

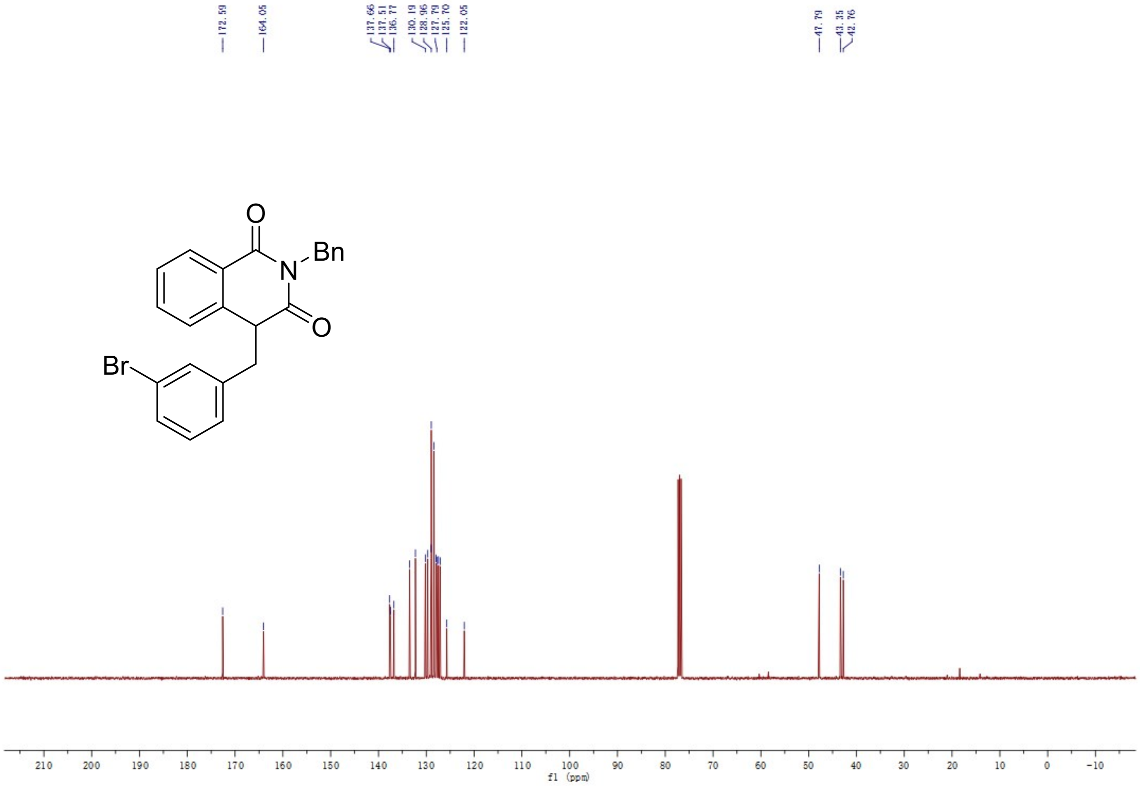

2-benzyl-4-(2-methoxybenzyl)isoquinoline-1,3(2H,4H)-dione (4m):

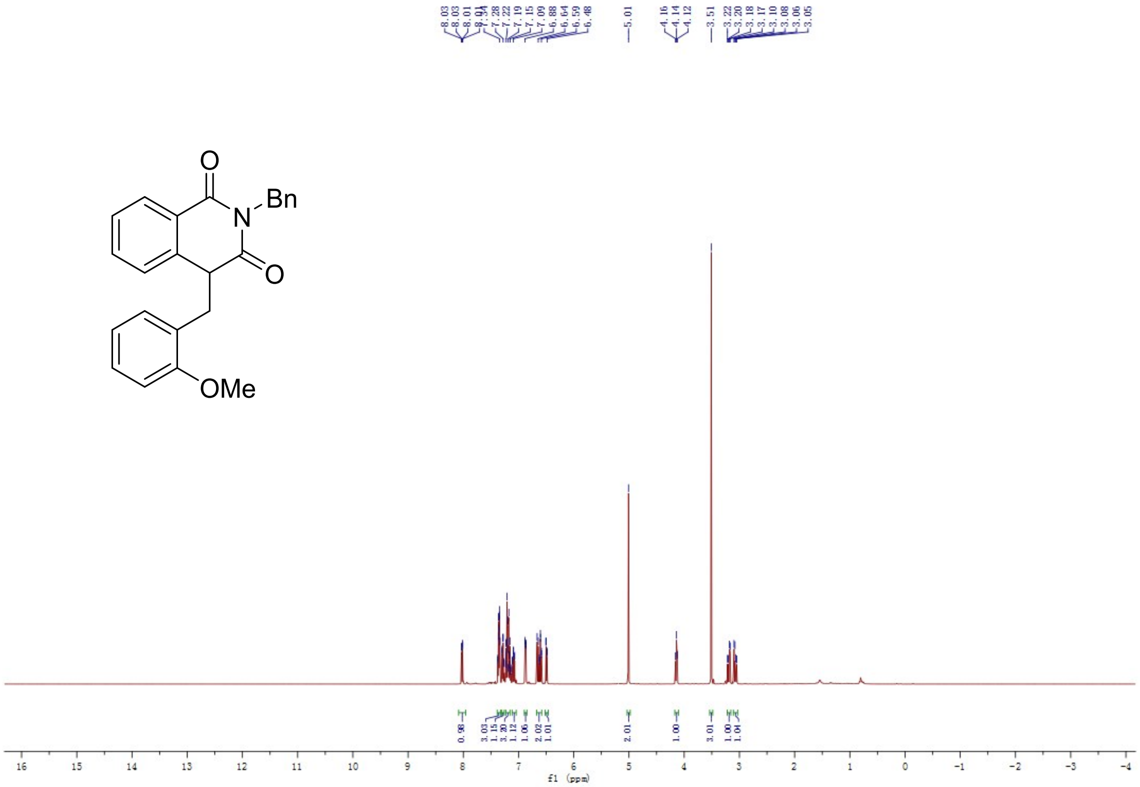

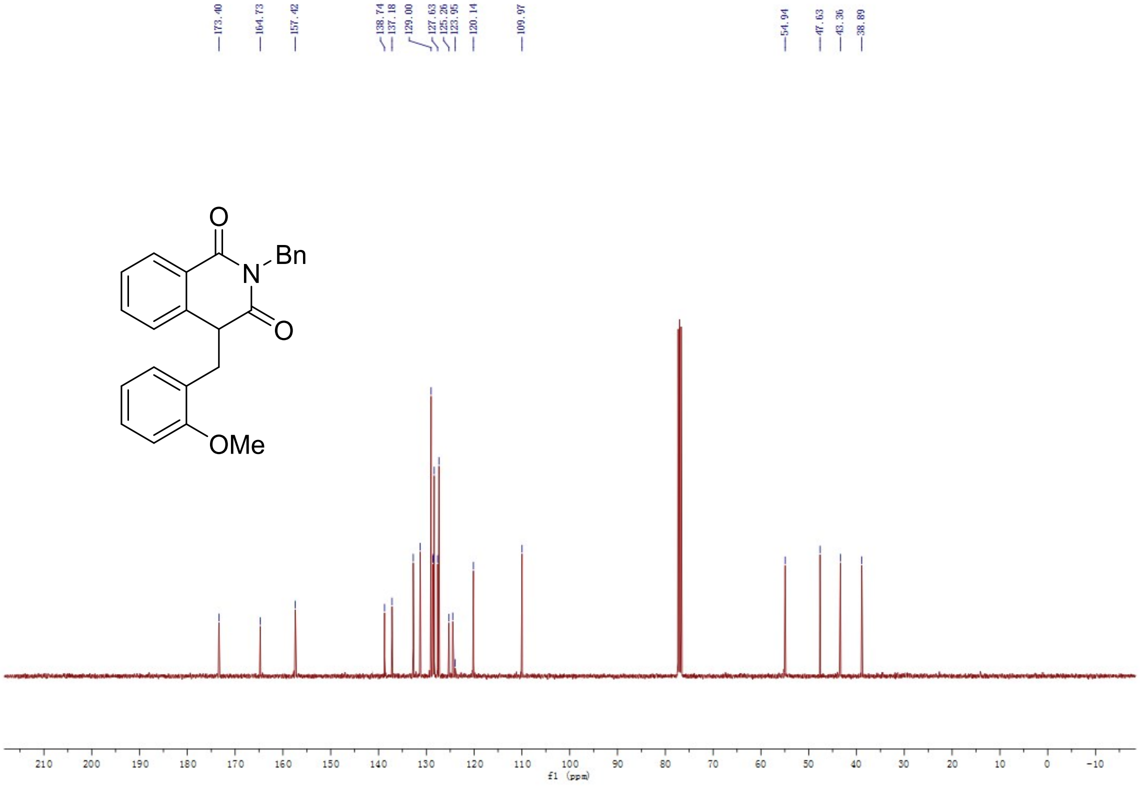

2-benzyl-4-(2-methylbenzyl)isoquinoline-1,3(2H,4H)-dione (4n):

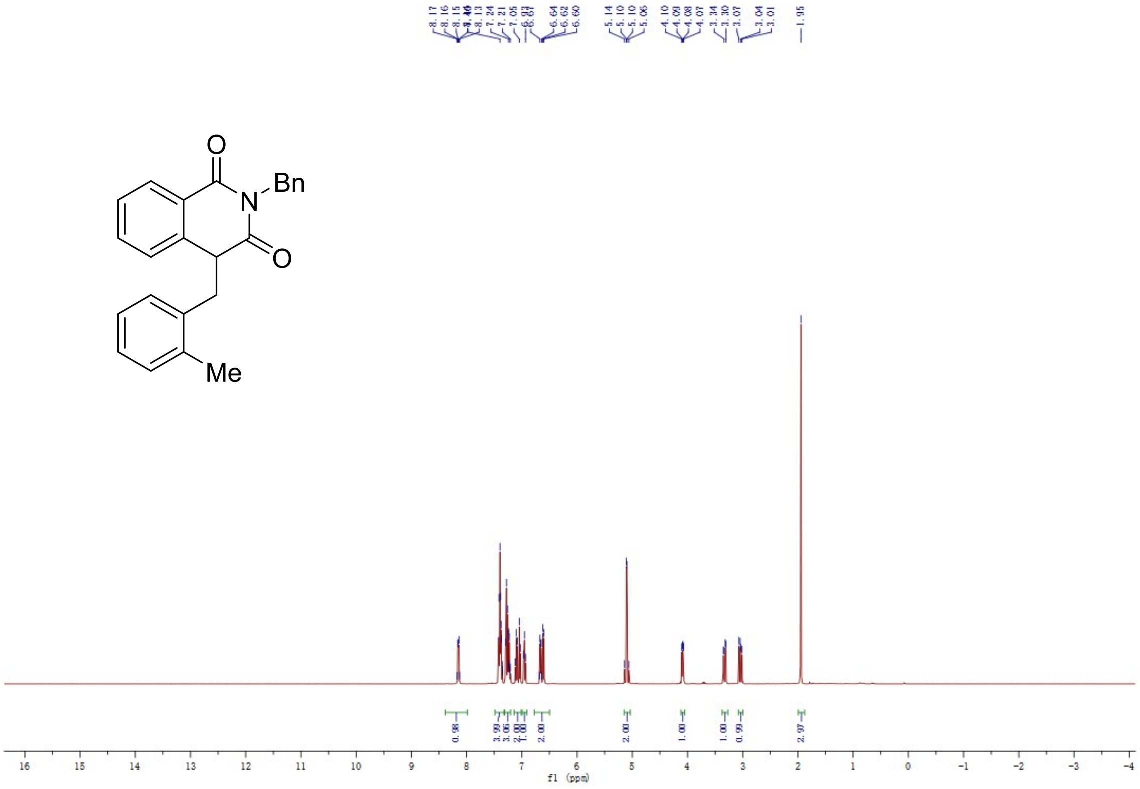

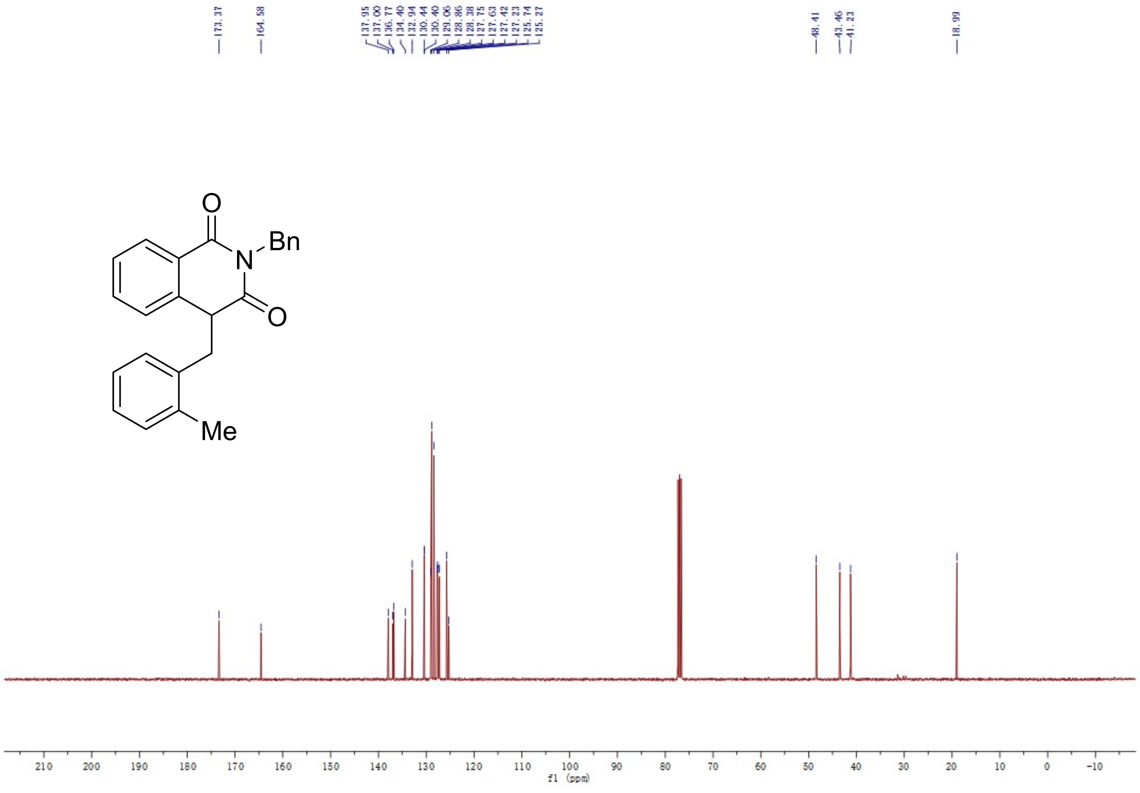

2-benzyl-4-(2-chlorobenzyl)isoquinoline-1,3(2H,4H)-dione (4o):

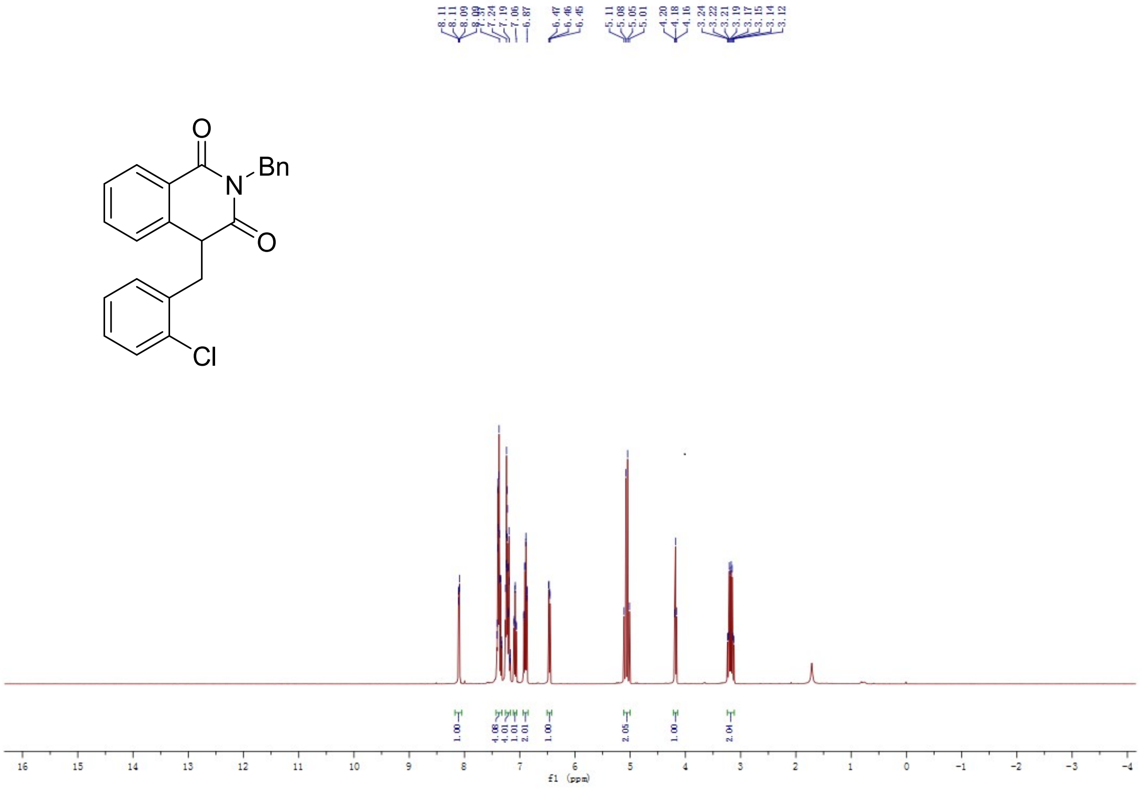

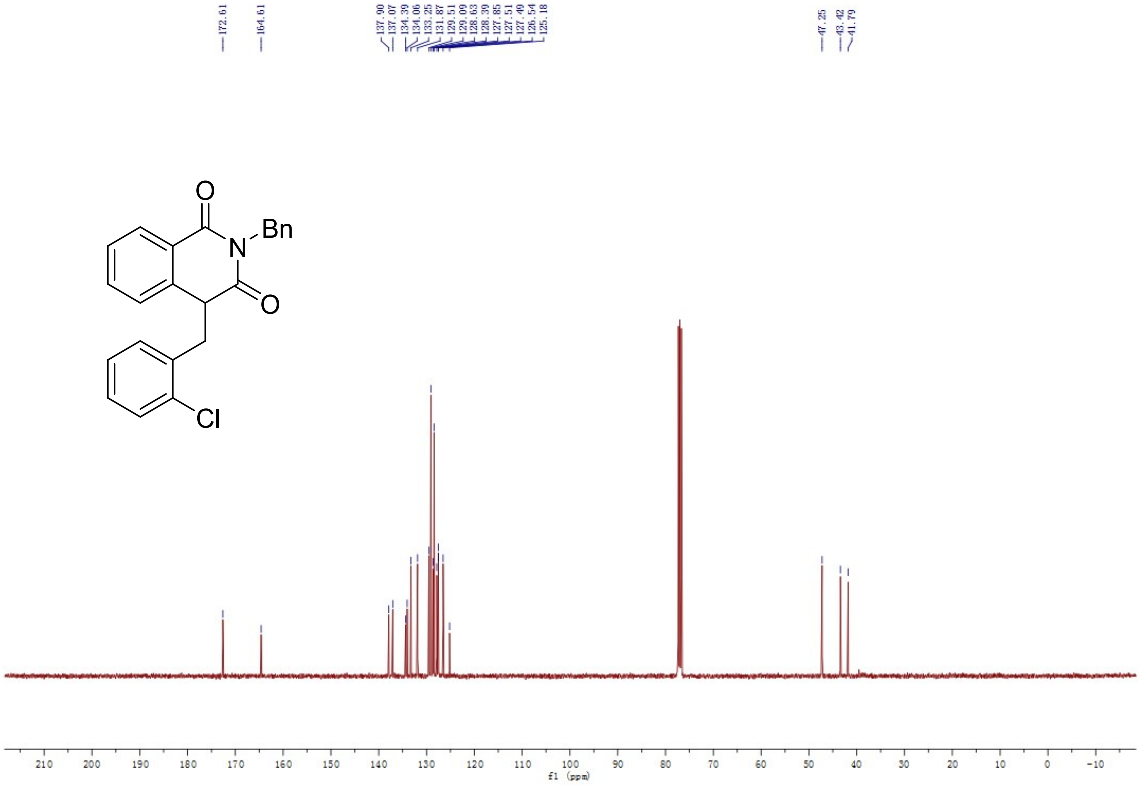

2-benzyl-4-(3,4,5-trimethylbenzyl)isoquinoline-1,3(2H,4H)-dione (4p):

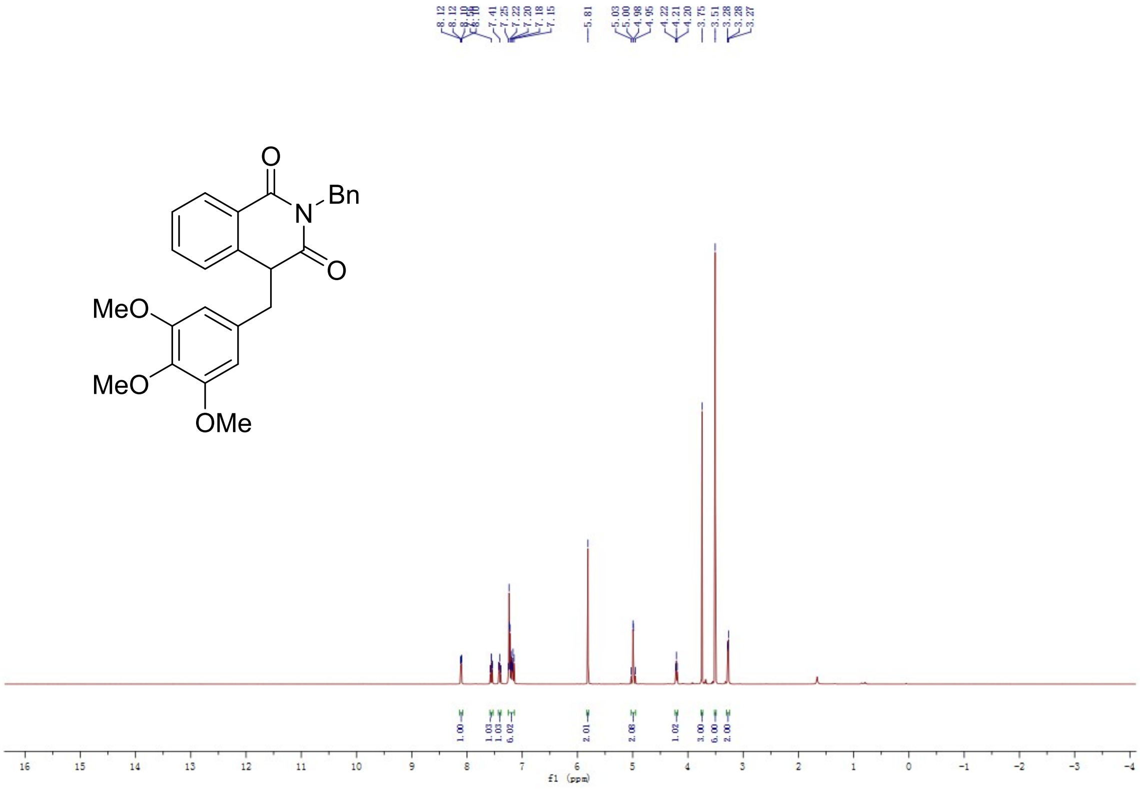

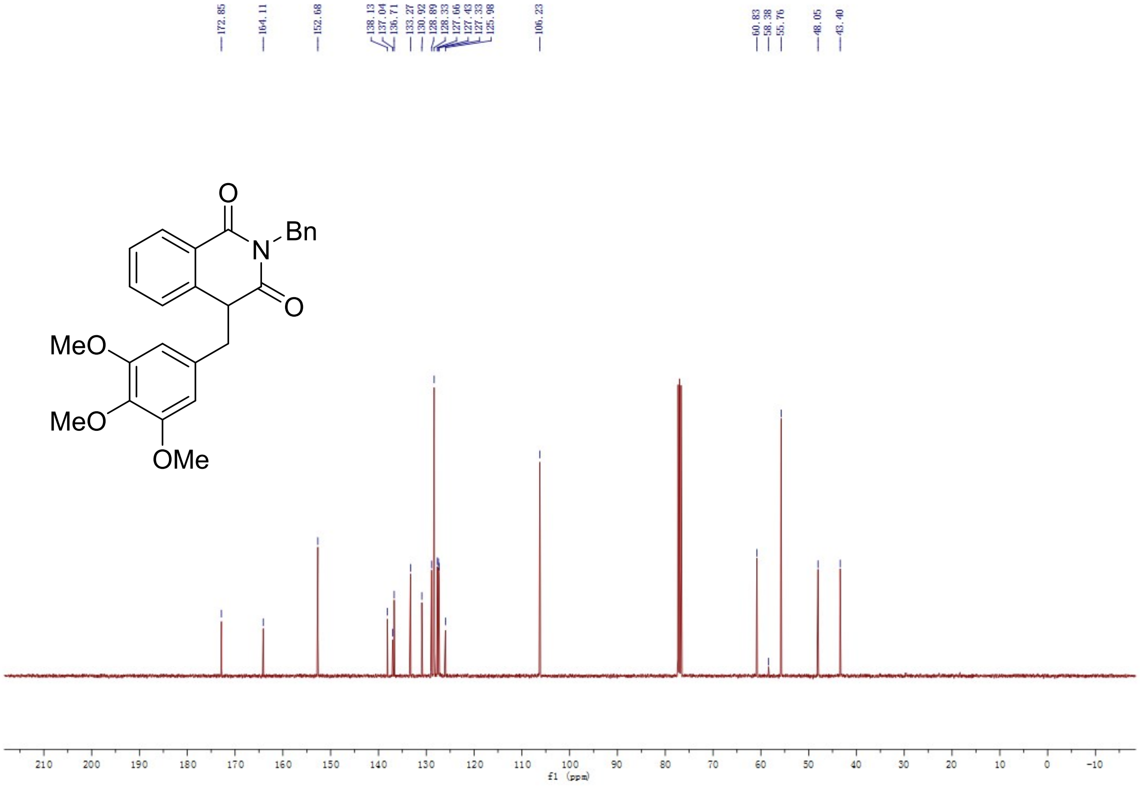

2-benzyl-4-(naphthalen-2-ylmethyl)isoquinoline-1,3(2H,4H)-dione (4q):

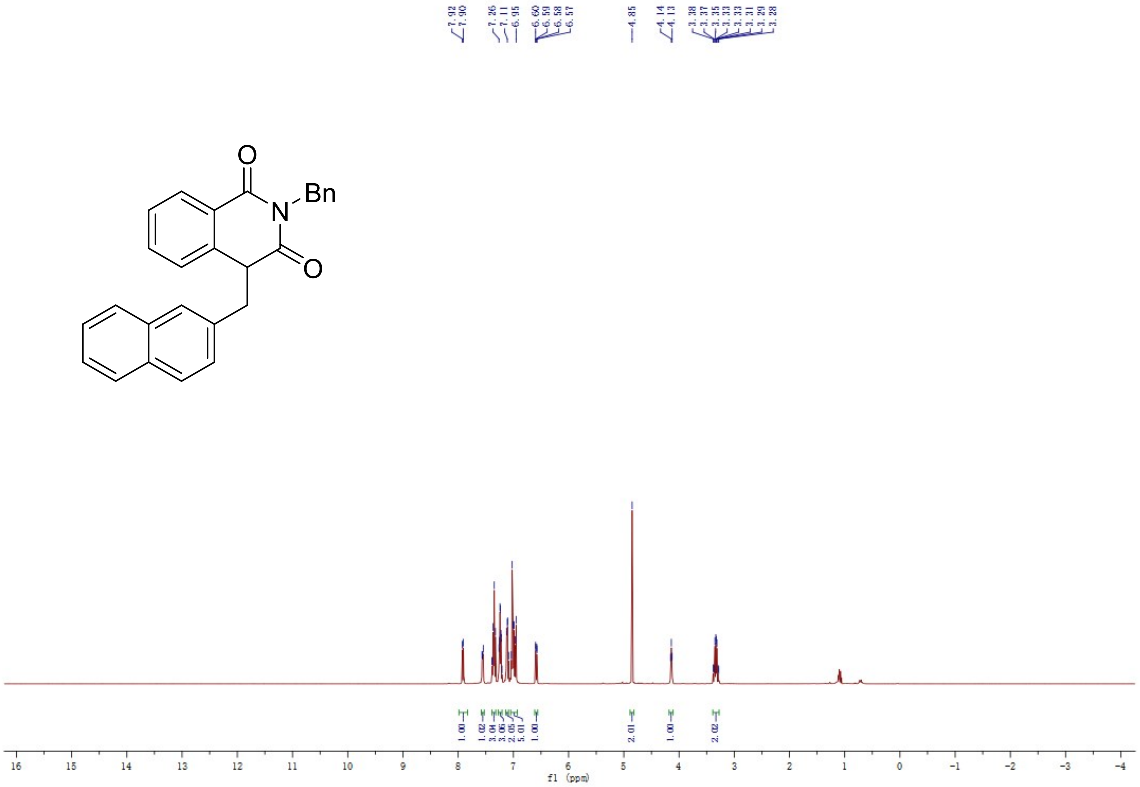

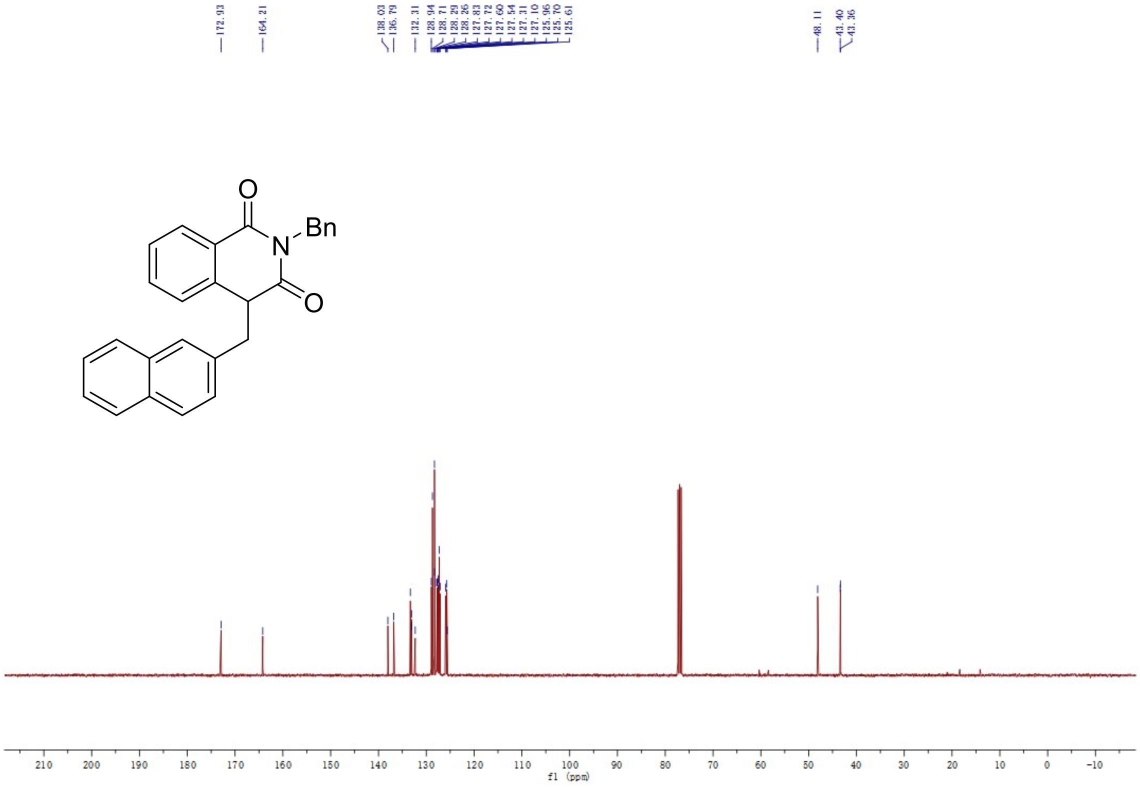

4-((1H-indol-2-yl)methyl)-2-benzylisoquinoline-1,3(2H,4H)-dione (4r):

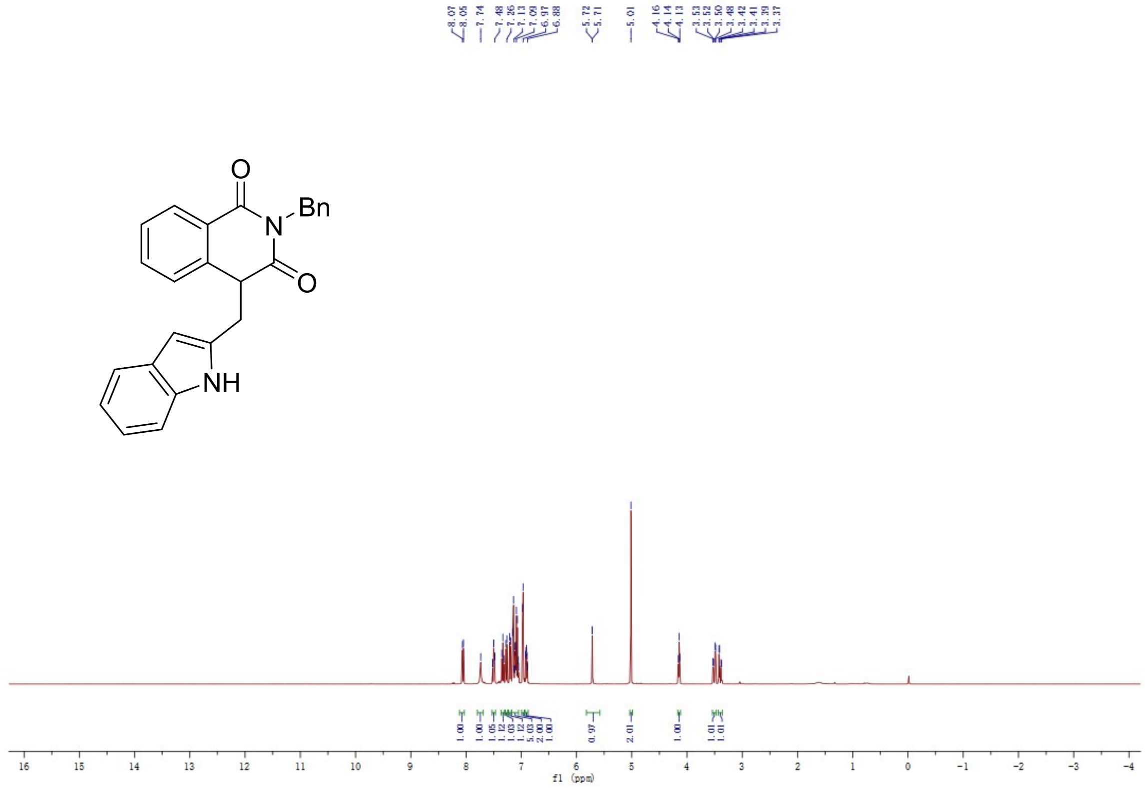

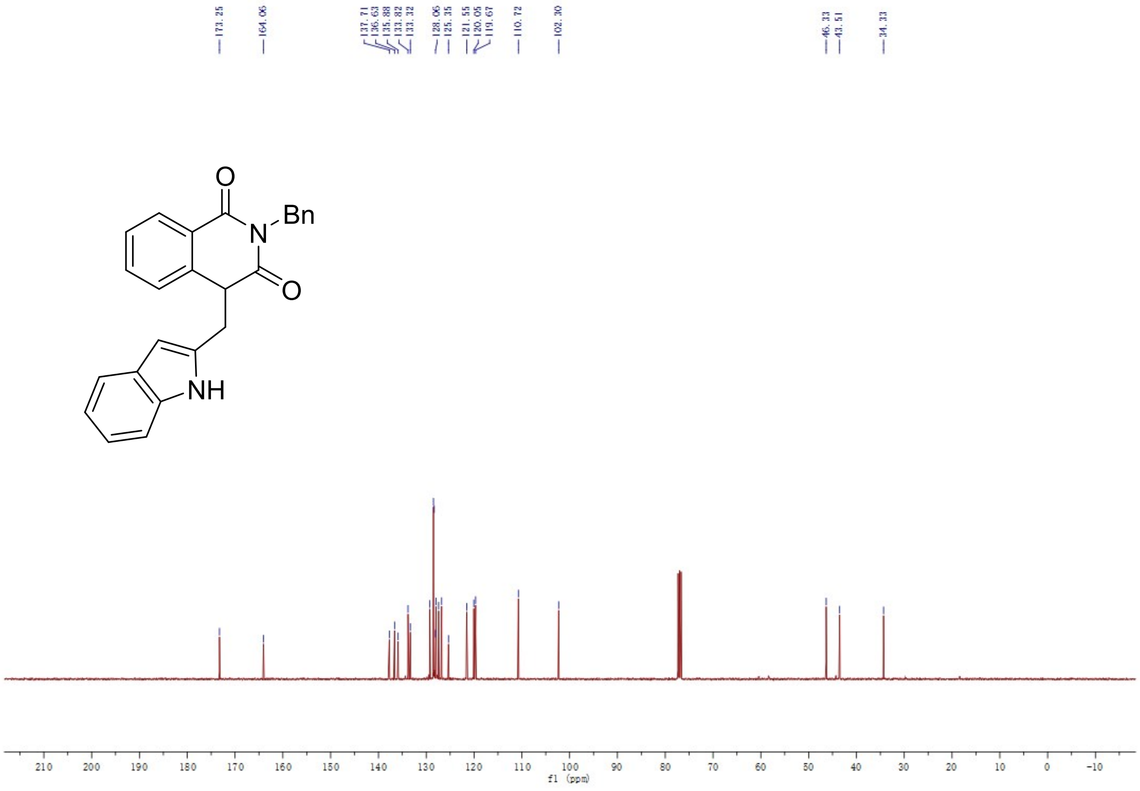

4-((1H-indol-3-yl)methyl)-2-benzylisoquinoline-1,3(2H,4H)-dione (4s):

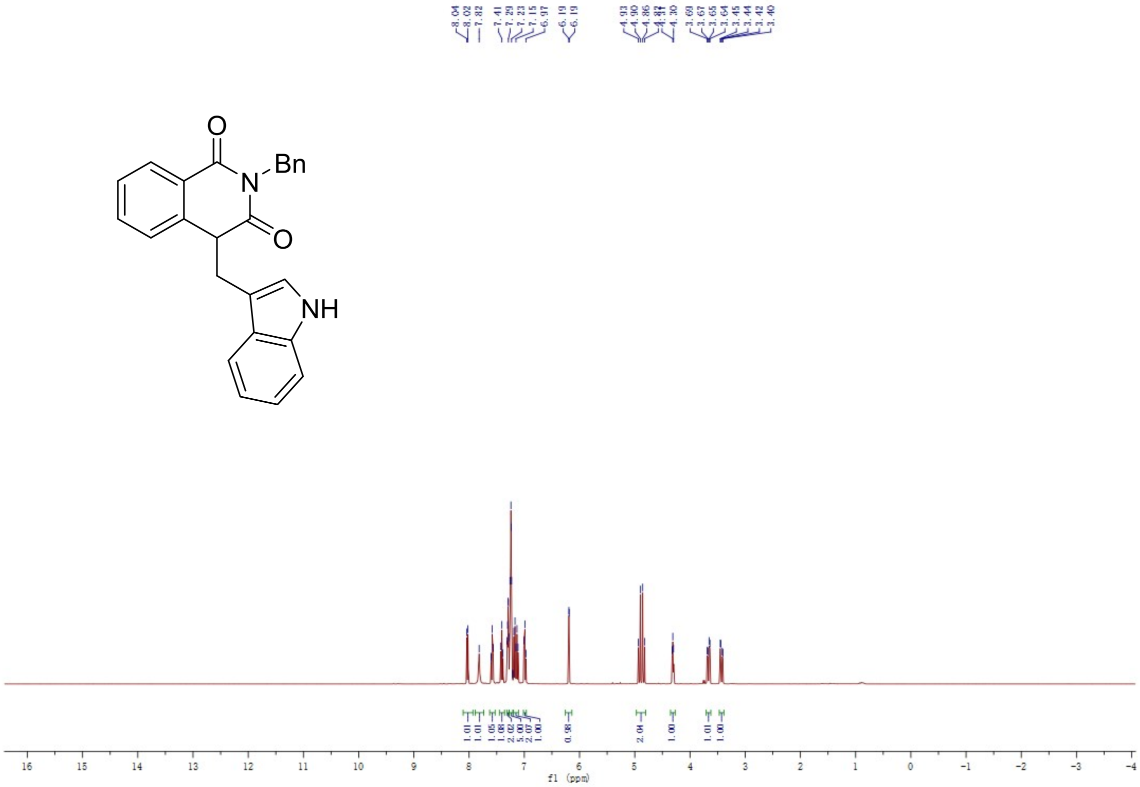

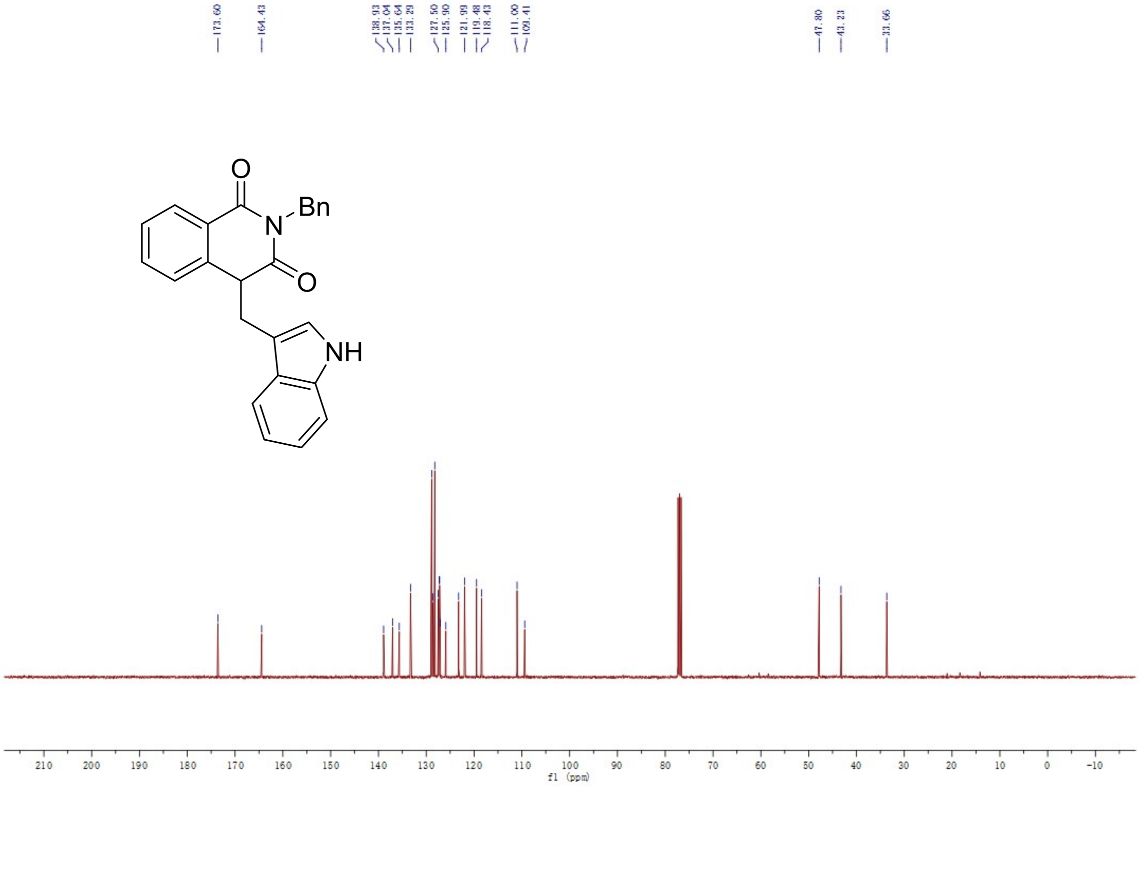

2-benzyl-4-((2-methyl-1H-indol-3-yl)methyl)isoquinoline-1,3(2H,4H)-dione (4t):

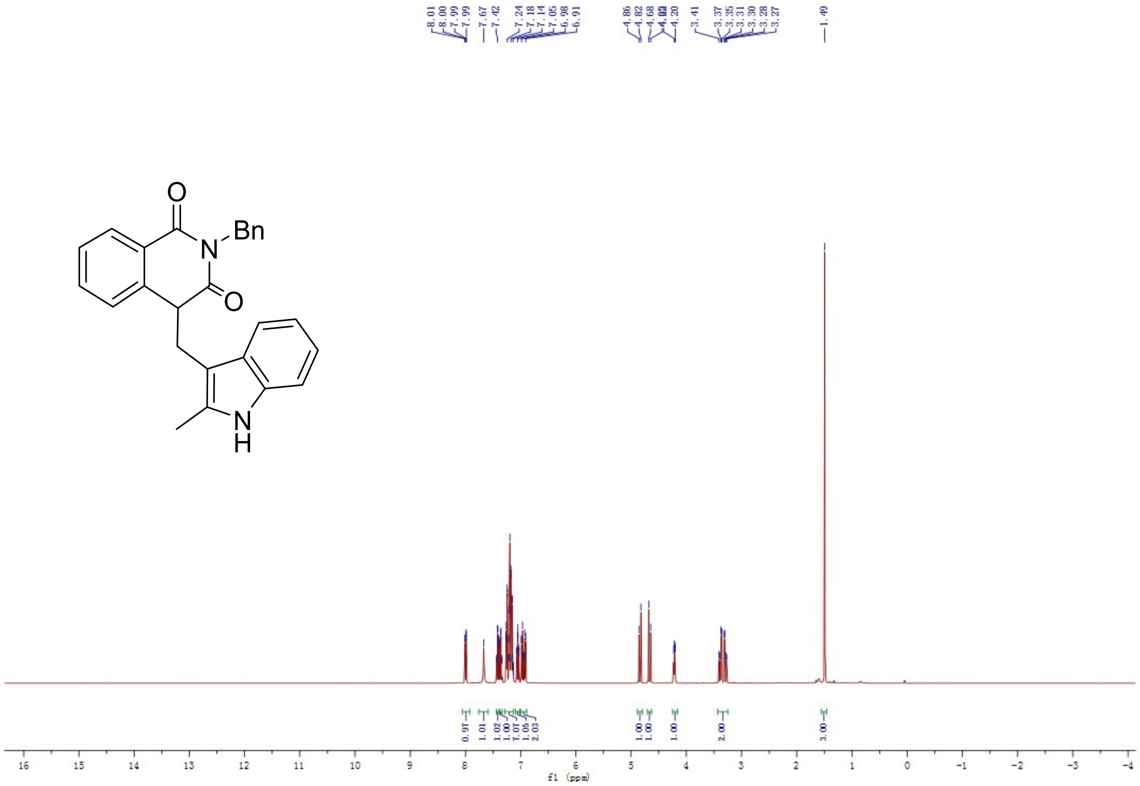

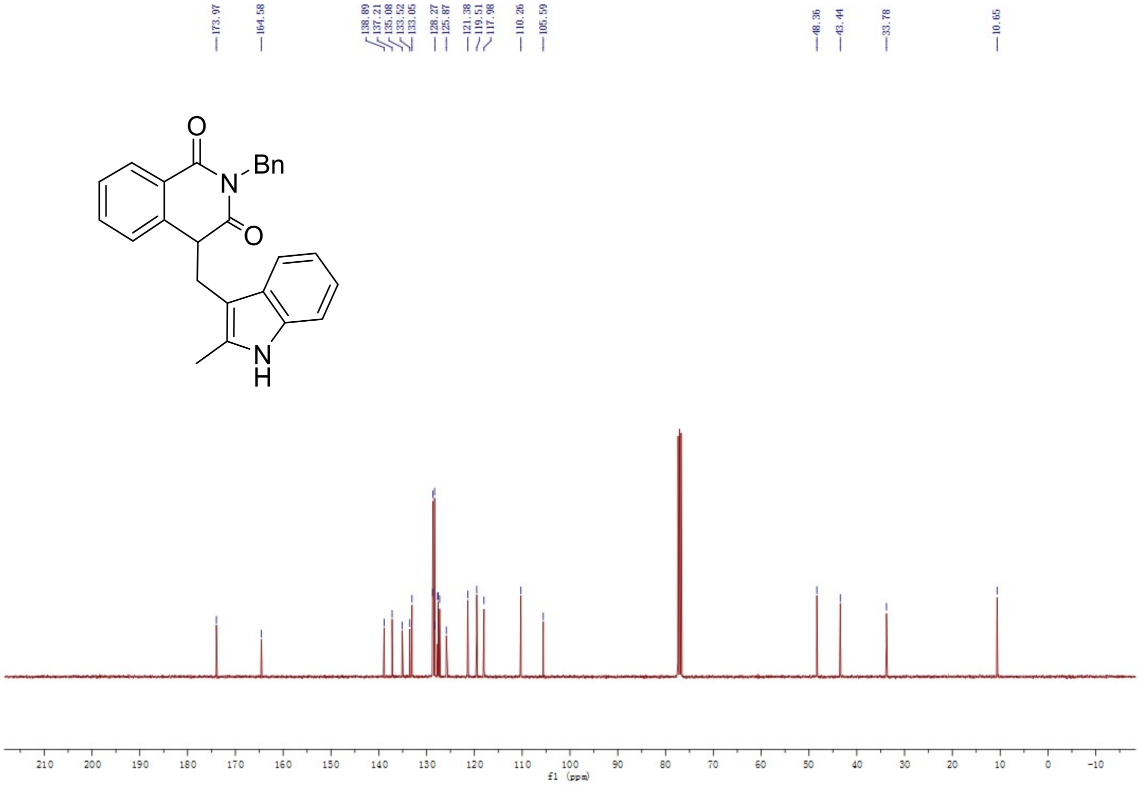

2-benzyl-4-(furan-2-ylmethyl)isoquinoline-1,3(2H,4H)-dione (4u):

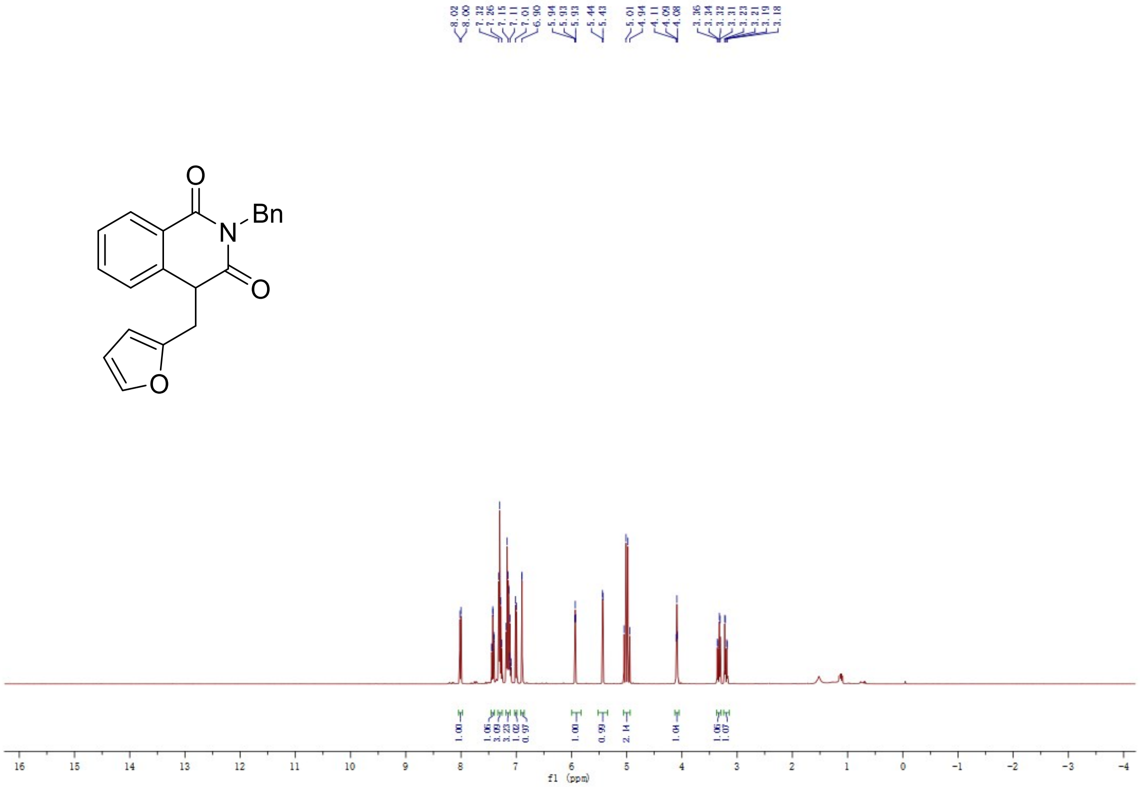

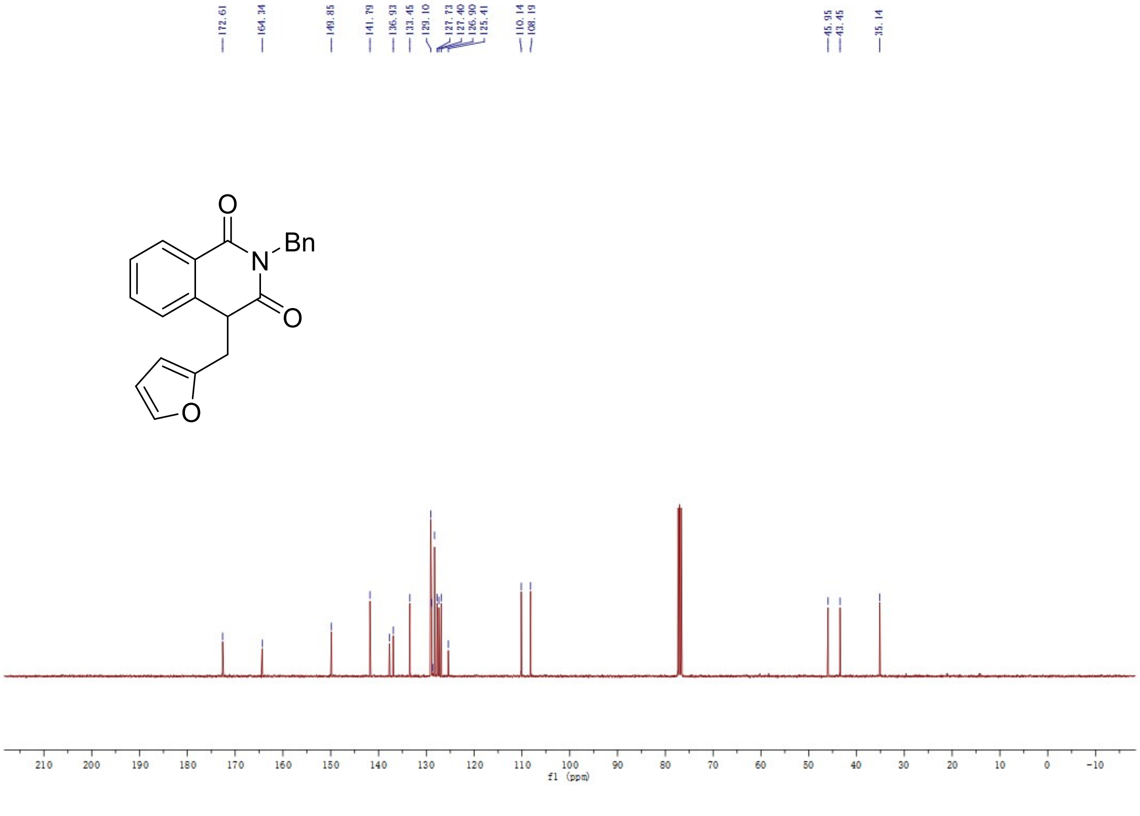

di-tert-butyl(S)-1-(2-benzyl-4-butyl-1,3-dioxo-1,2,3,4-tetrahydroisoquinolin-4-yl)hydrazine-1,2-dicarboxylate (6a):

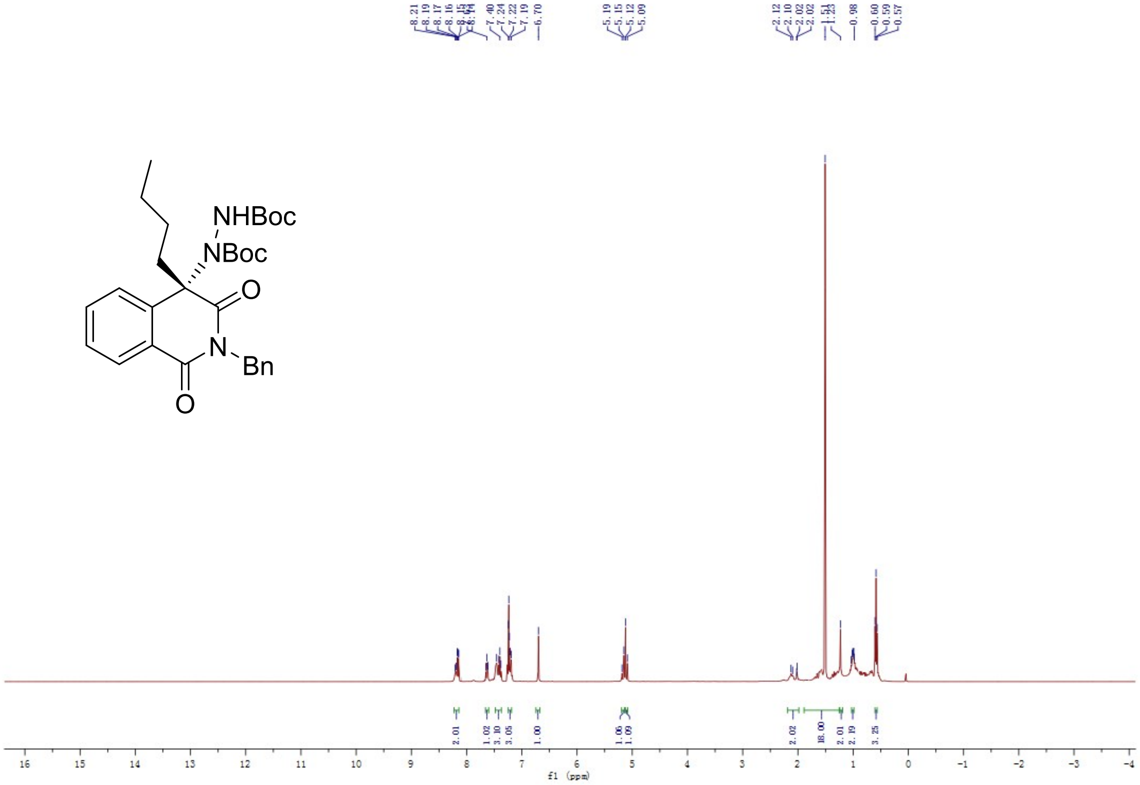

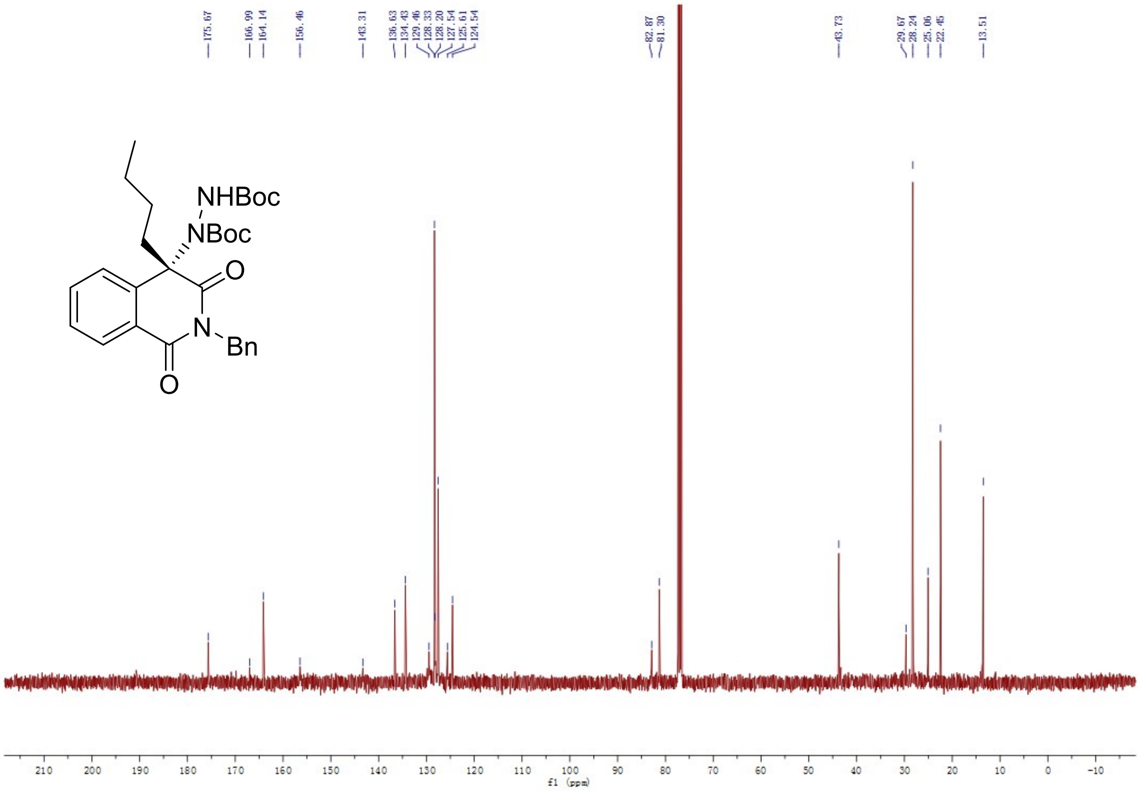

di-tert-butyl(S)-1-(2-benzyl-4-isopentyl-1,3-dioxo-1,2,3,4-tetrahydroisoquinolin-4-yl)hydrazine-1,2-dicarboxylate (6b):

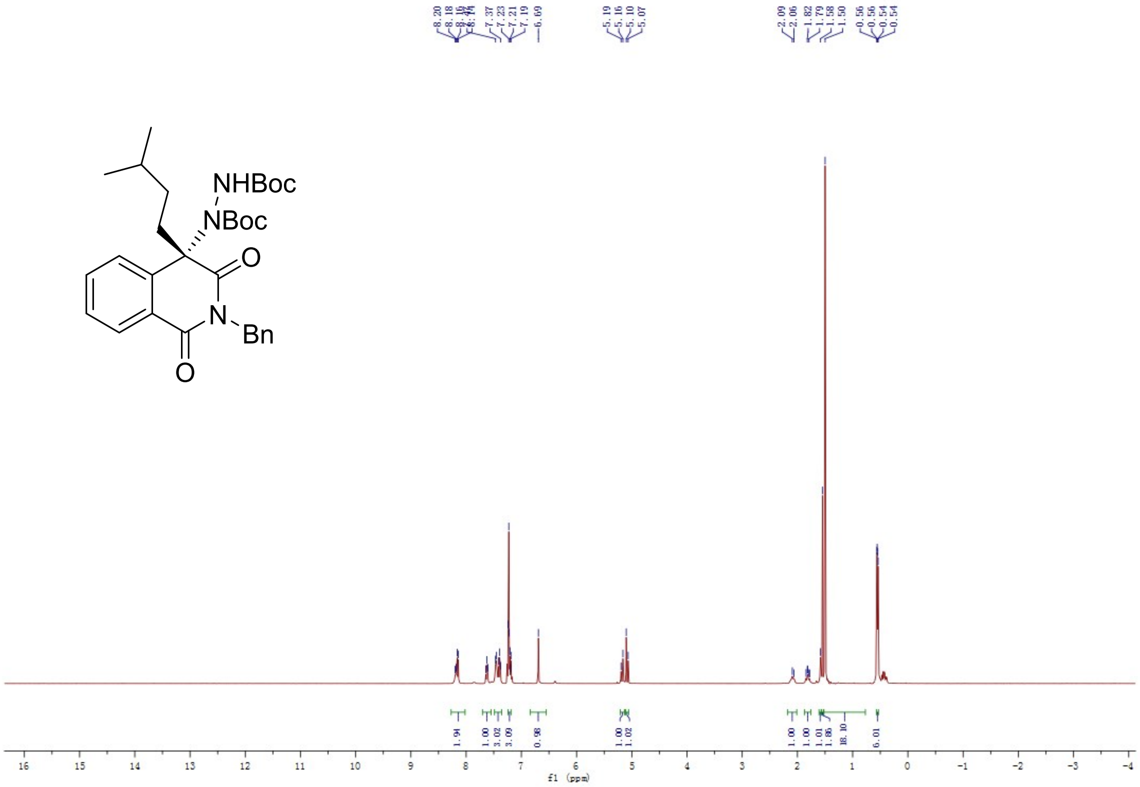

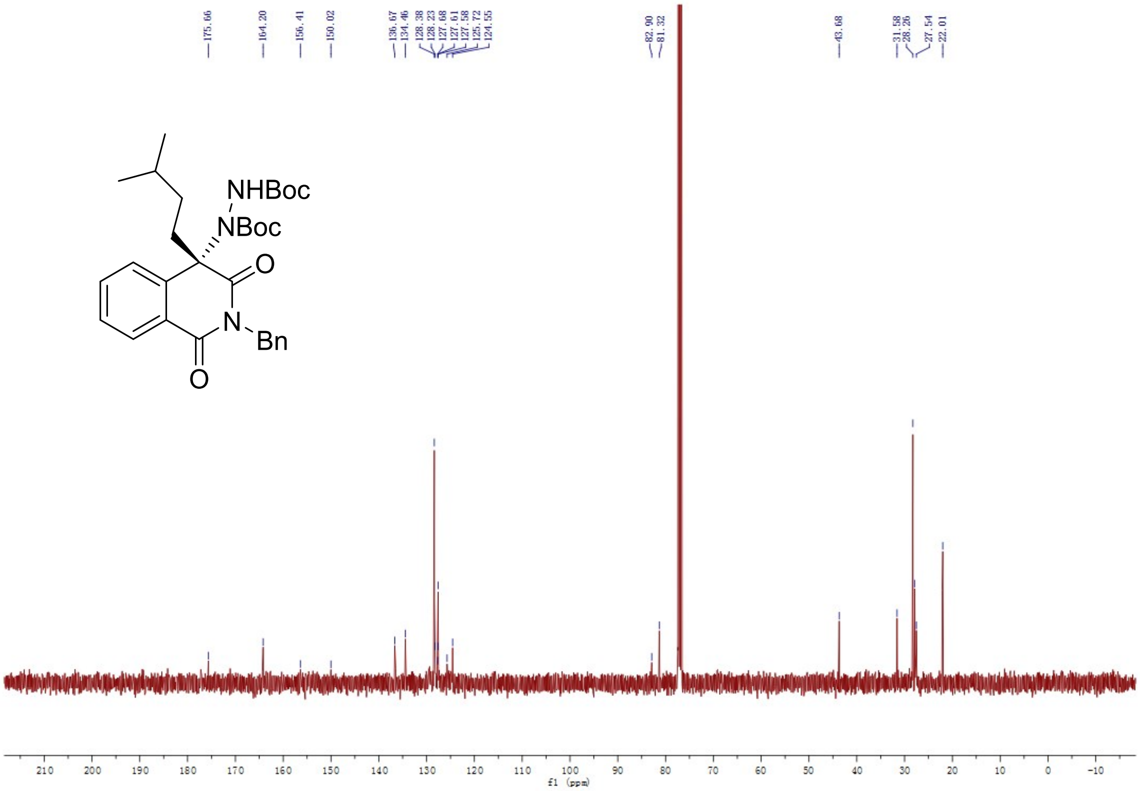

di-tert-butyl(S)-1-(2-benzyl-4-isobutyl-1,3-dioxo-1,2,3,4-tetrahydroisoquinolin-4-yl)hydrazine-1,2-dicarboxylate (6c):

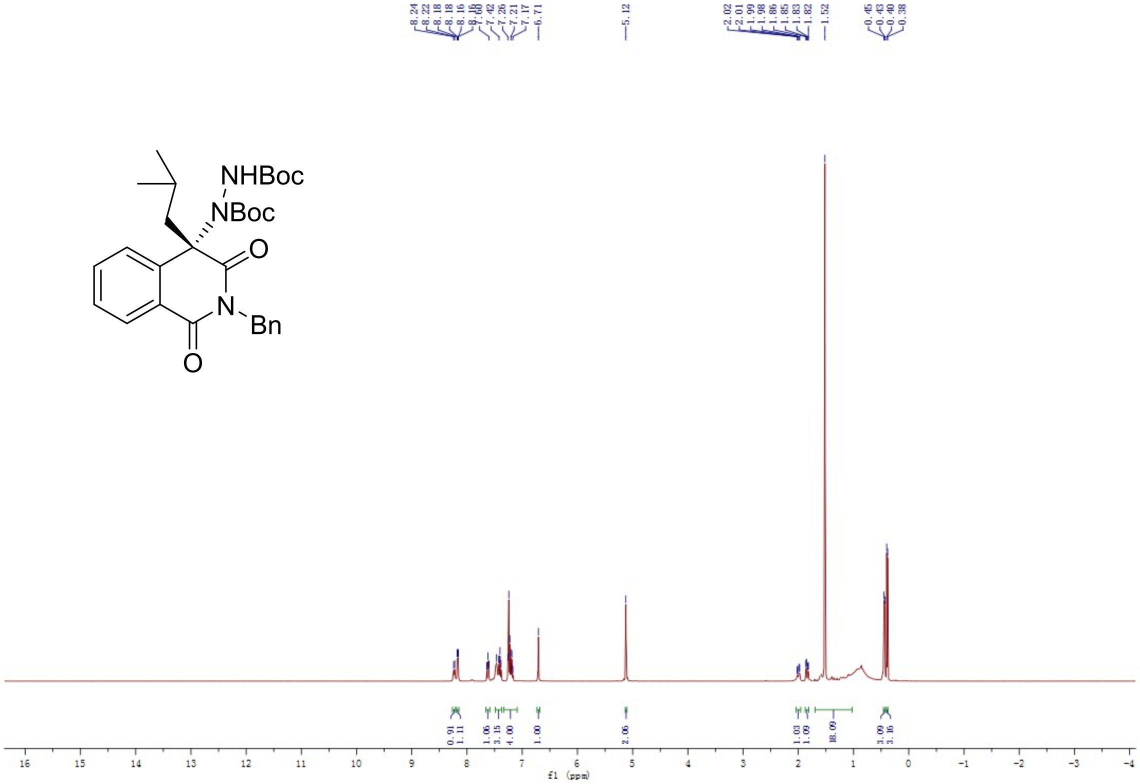

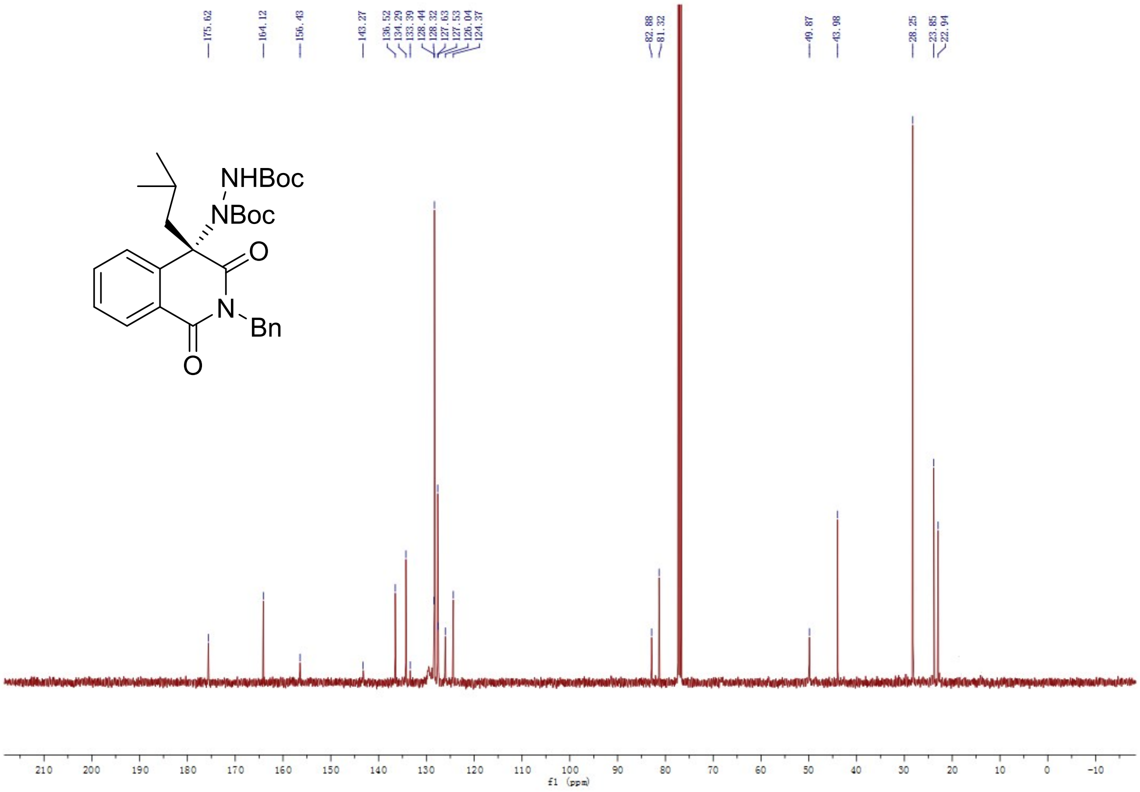

di-tert-butyl(S)-1-(2-benzyl-1,3-dioxo-4-(3-phenylpropyl)-1,2,3,4-tetrahydroisoquinolin-4-yl)hydrazine-1,2-dicarboxylate(6d):

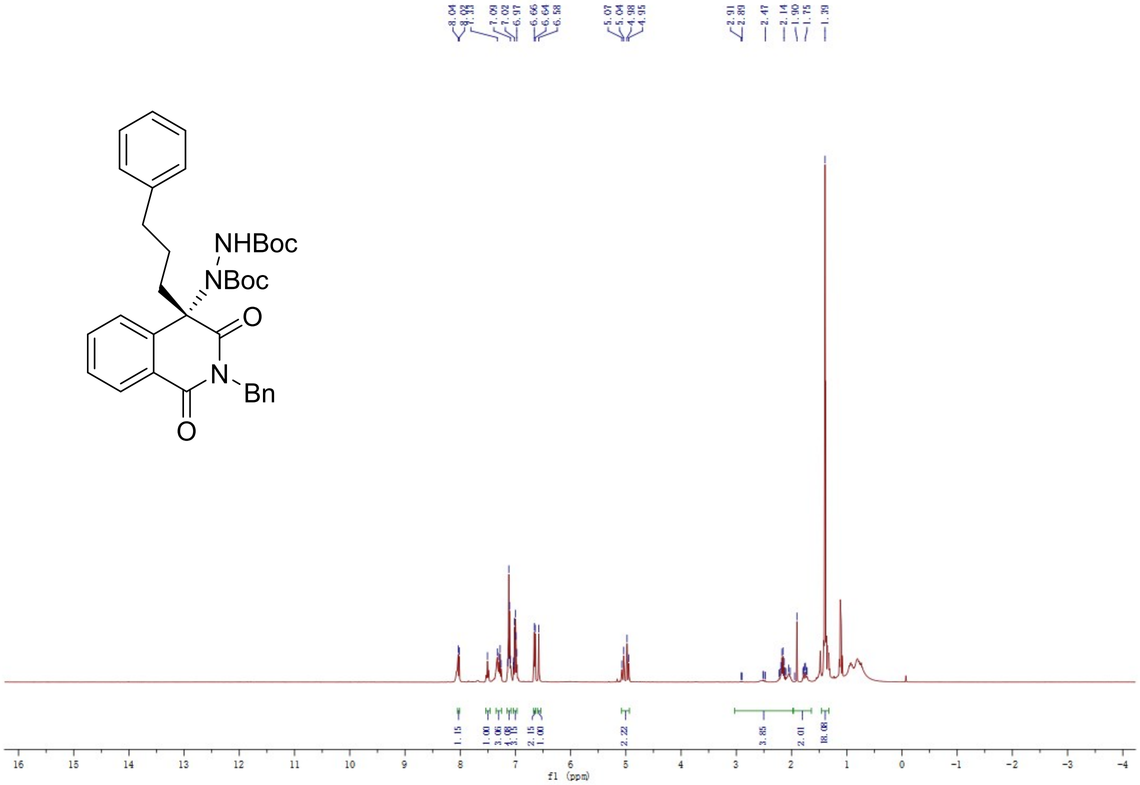

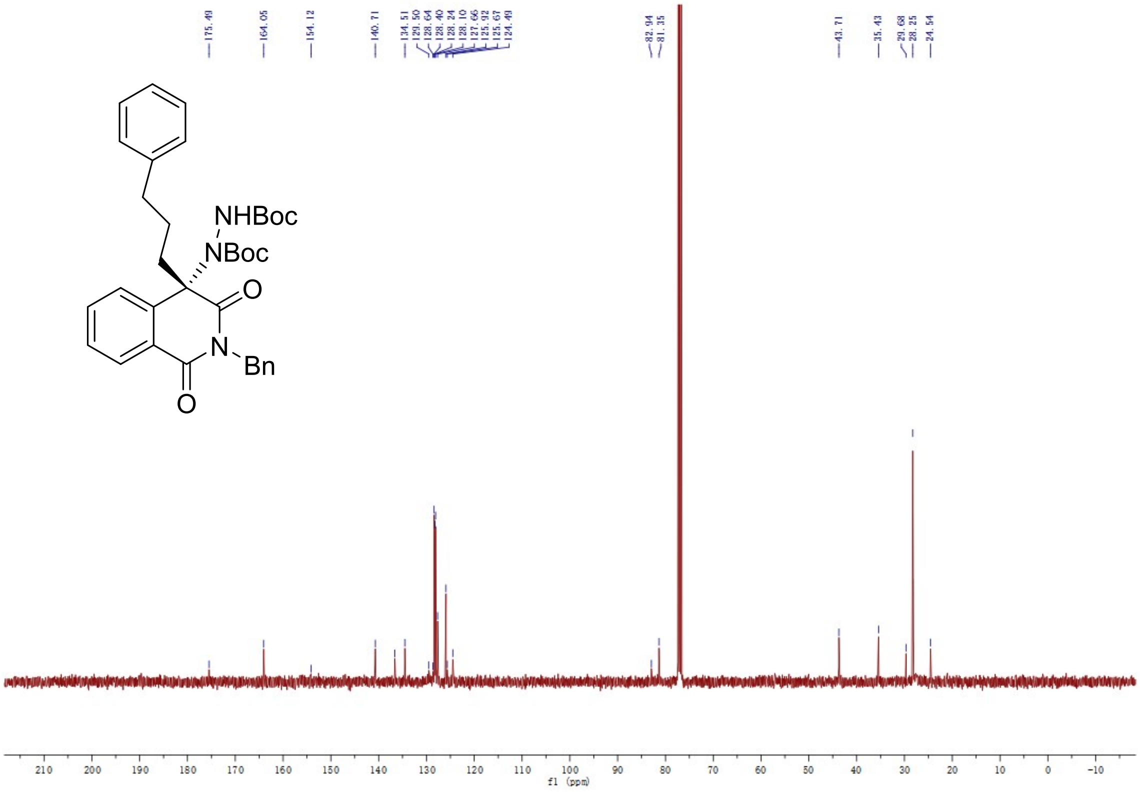

(di-tert-butyl(S)-1-(2,4-dibenzyl-1,3-dioxo-1,2,3,4-tetrahydroisoquinolin-4-yl)hydrazine-1,2-dicarboxylate (6e):

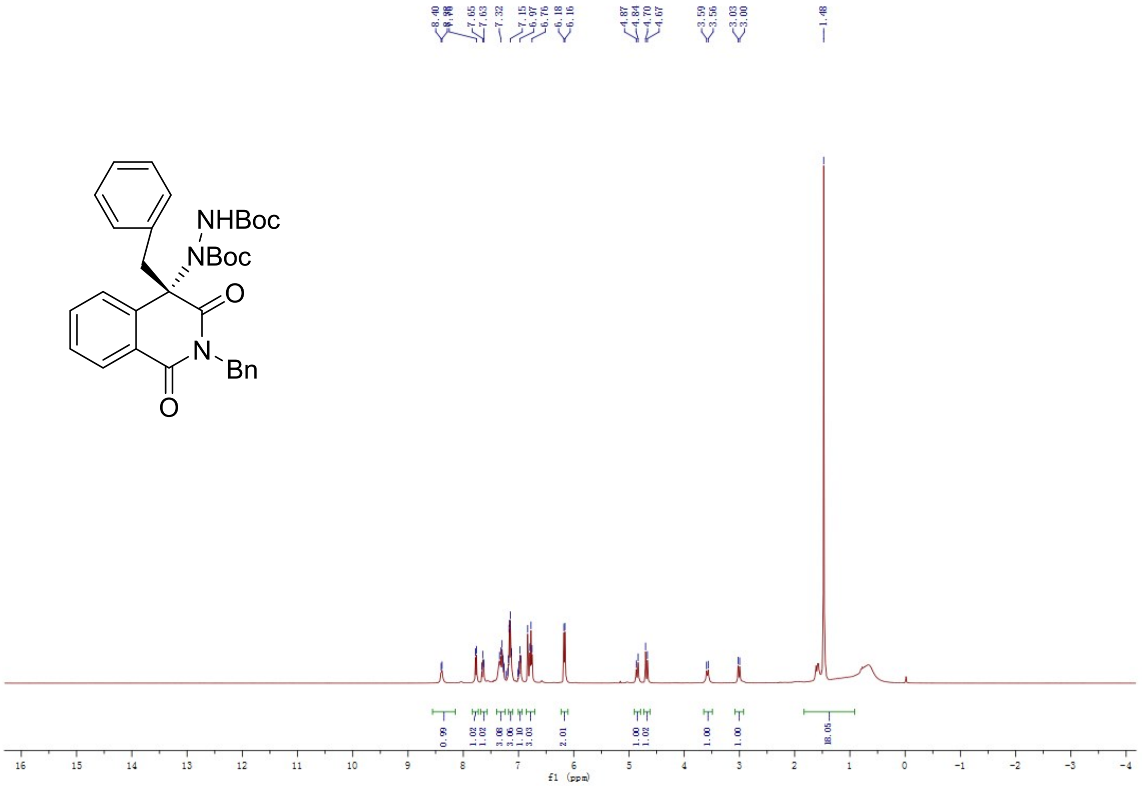

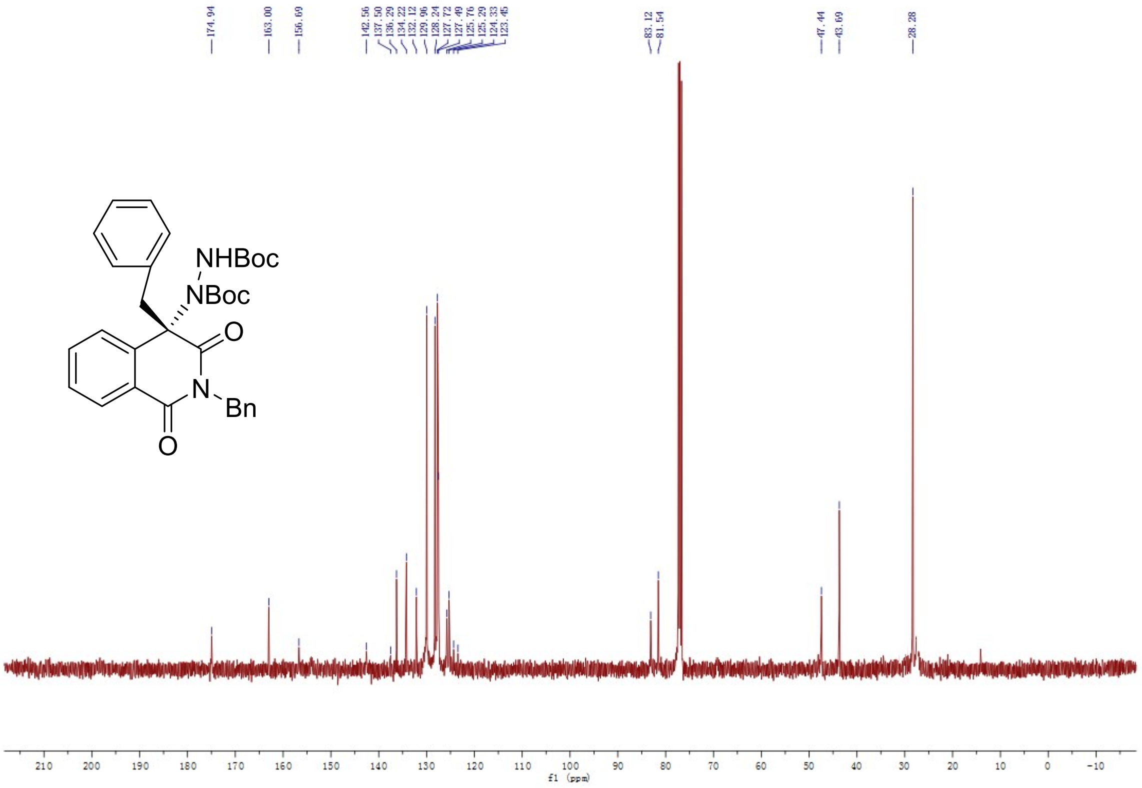

di-tert-butyl(S)-1-(2-benzyl-4-(4-methylbenzyl)-1,3-dioxo-1,2,3,4-tetrahydroisoquinolin-4-yl)hydrazine-1,2-dicarboxylate (6f):

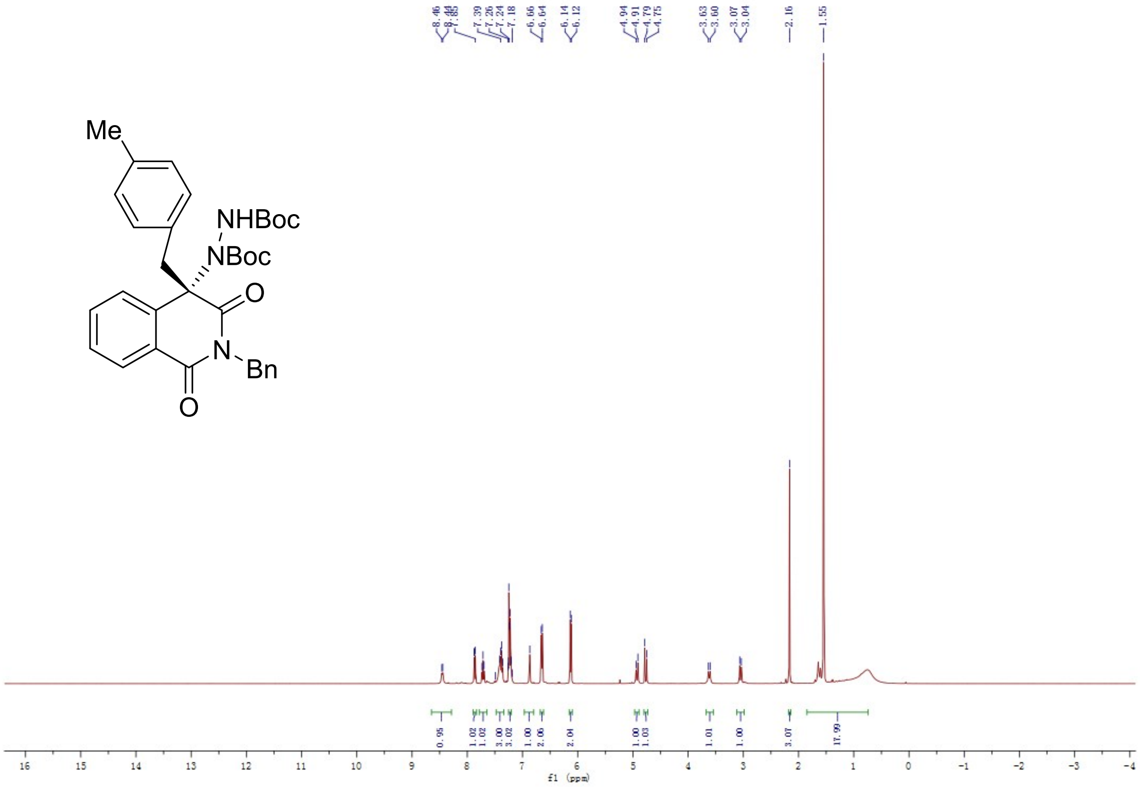

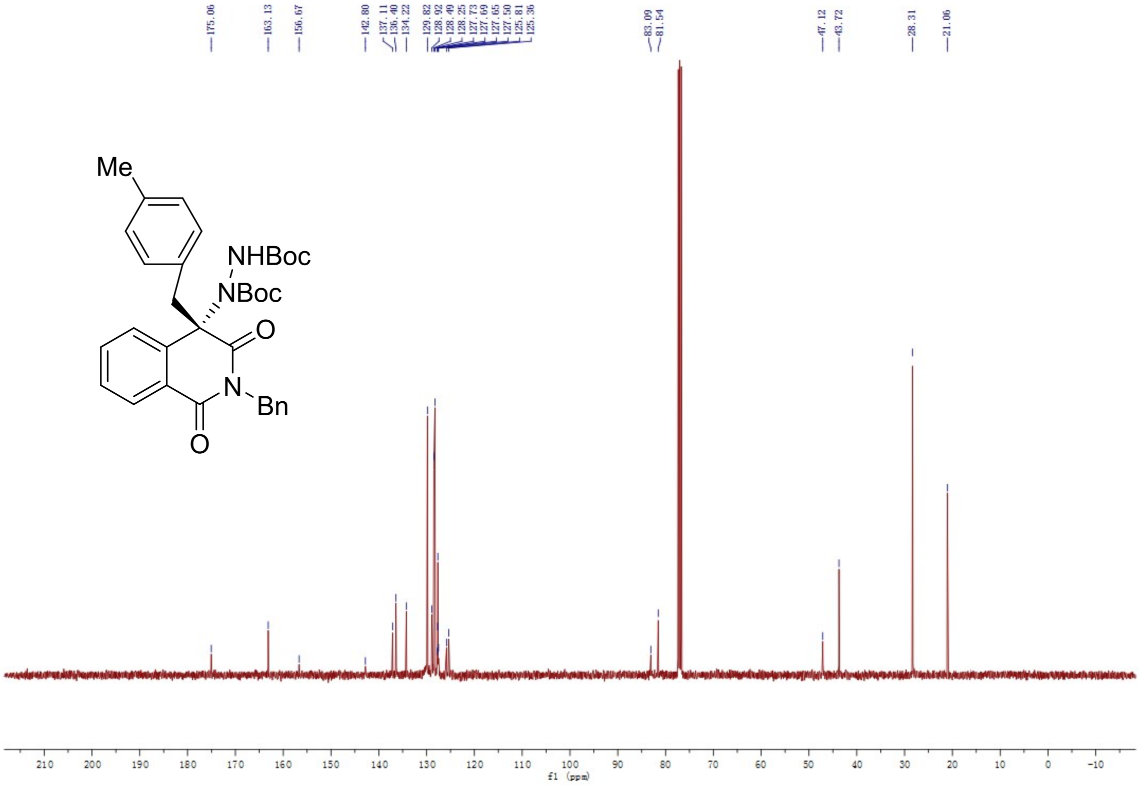

di-tert-butyl(S)-1-(2-benzyl-4-(4-methoxybenzyl)-1,3-dioxo-1,2,3,4-tetrahydroisoquinolin-4-yl)hydrazine-1,2-dicarboxylate (6g):

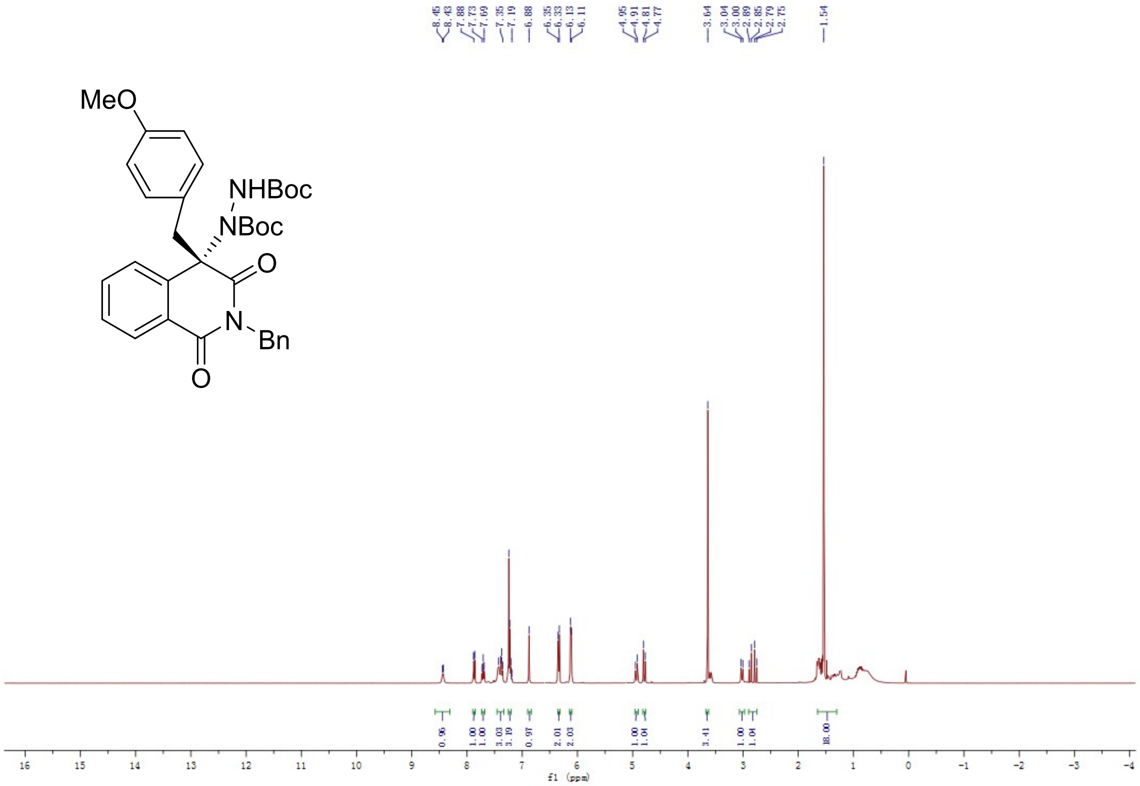

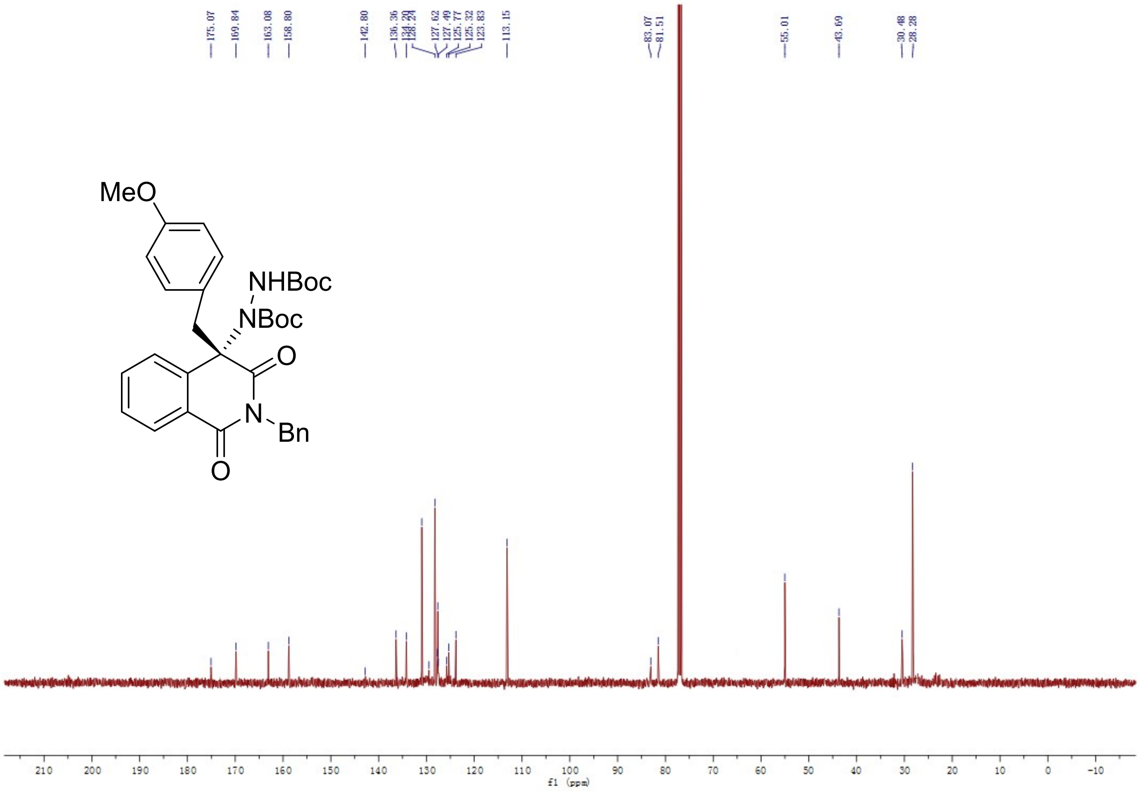

di-tert-butyl(S)-1-(2-benzyl-4-(4-fluorobenzyl)-1,3-dioxo-1,2,3,4-tetrahydroisoquinolin-4-yl)hydrazine-1,2-dicarboxylate (6h):

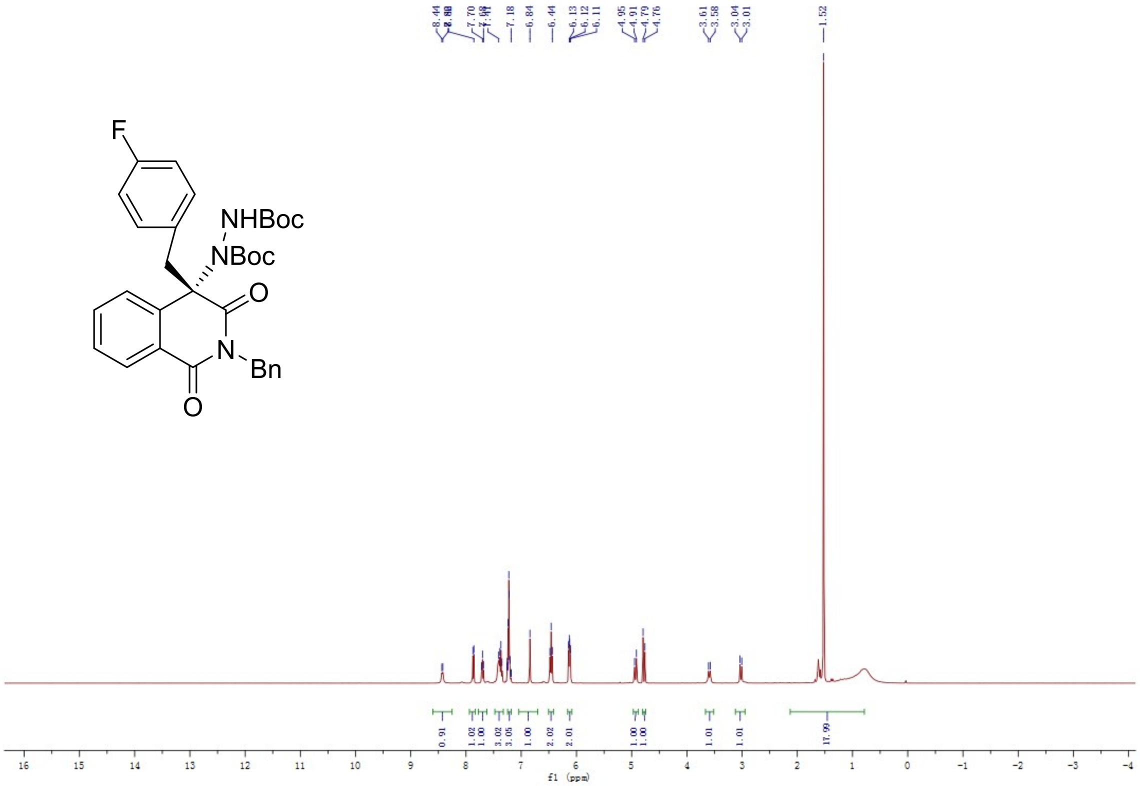

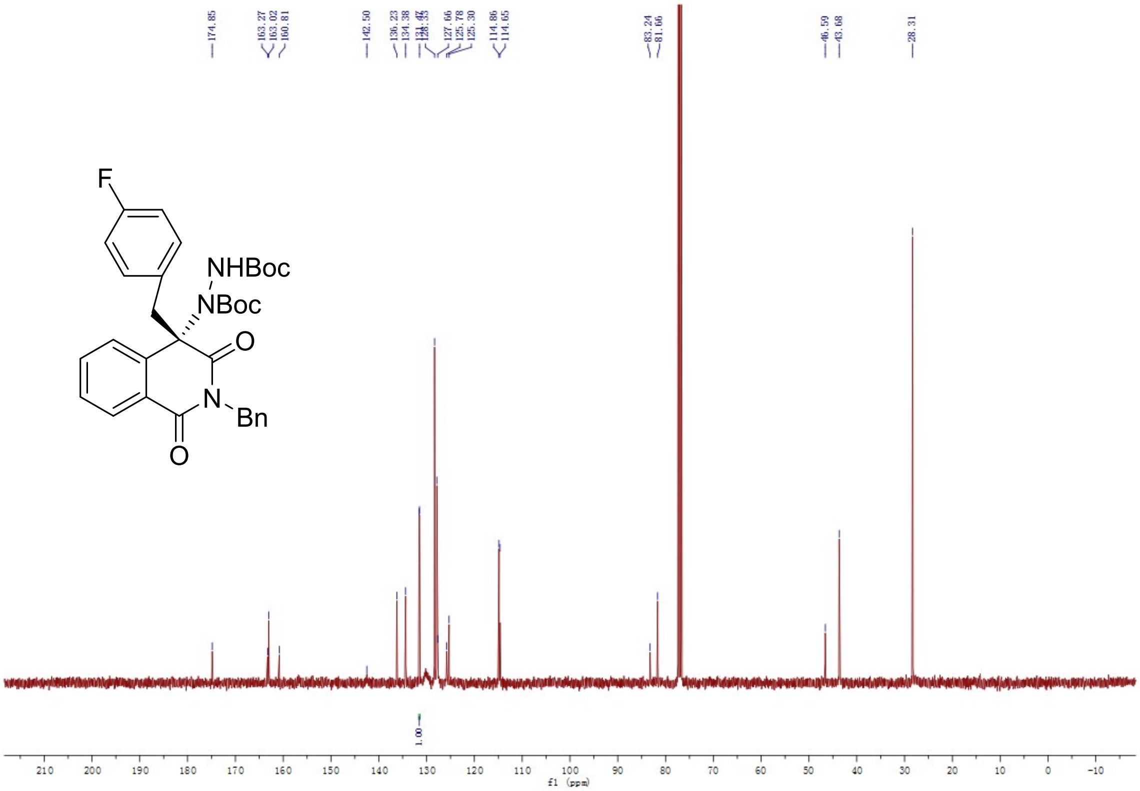

di-tert-butyl(S)-1-(2-benzyl-4-(4-chlorobenzyl)-1,3-dioxo-1,2,3,4-tetrahydroisoquinolin-4-yl)hydrazine-1,2-dicarboxylate(6i):

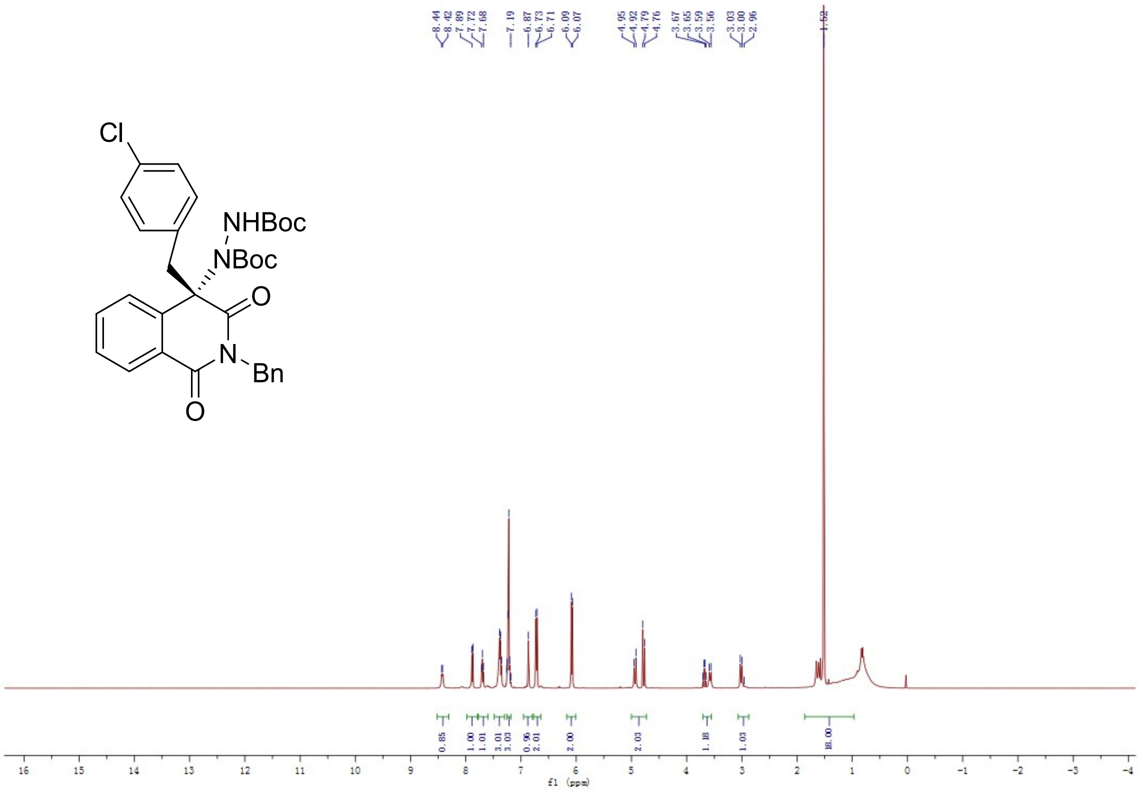

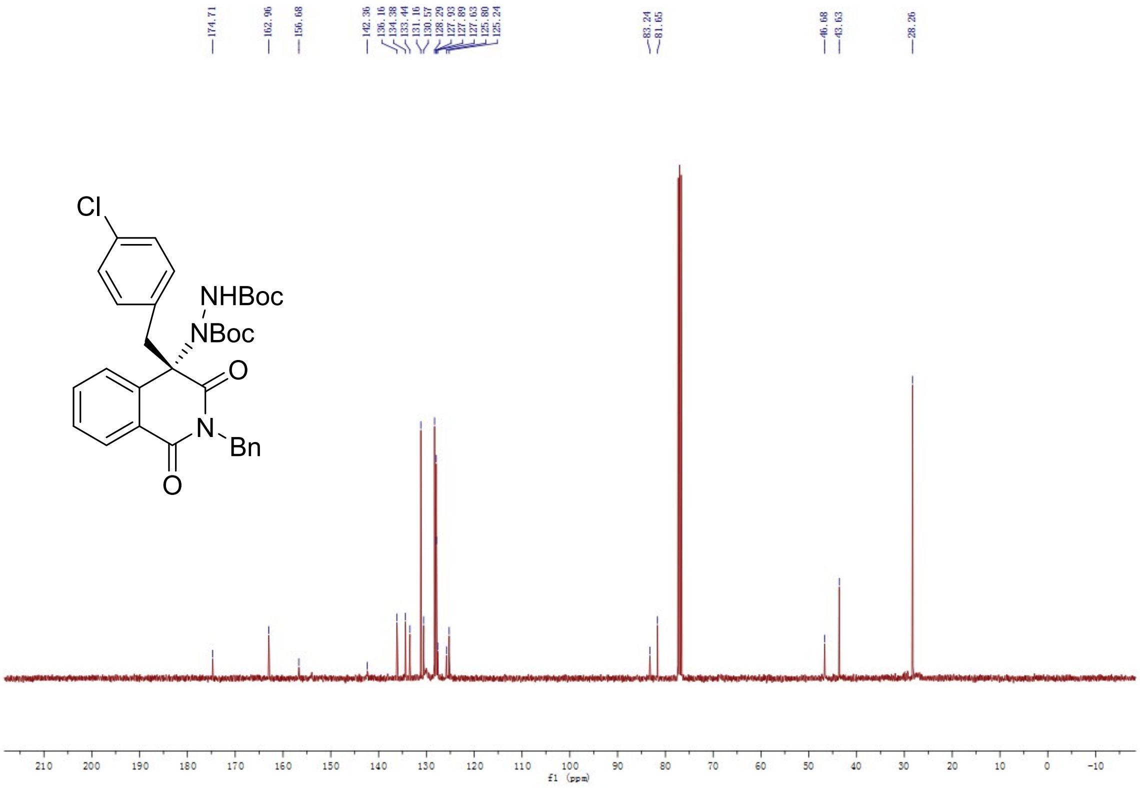

di-tert-butyl(S)-1-(2-benzyl-4-(4-bromobenzyl)-1,3-dioxo-1,2,3,4-tetrahydroisoquinolin-4-yl)hydrazine-1,2-dicarboxylate (6j):

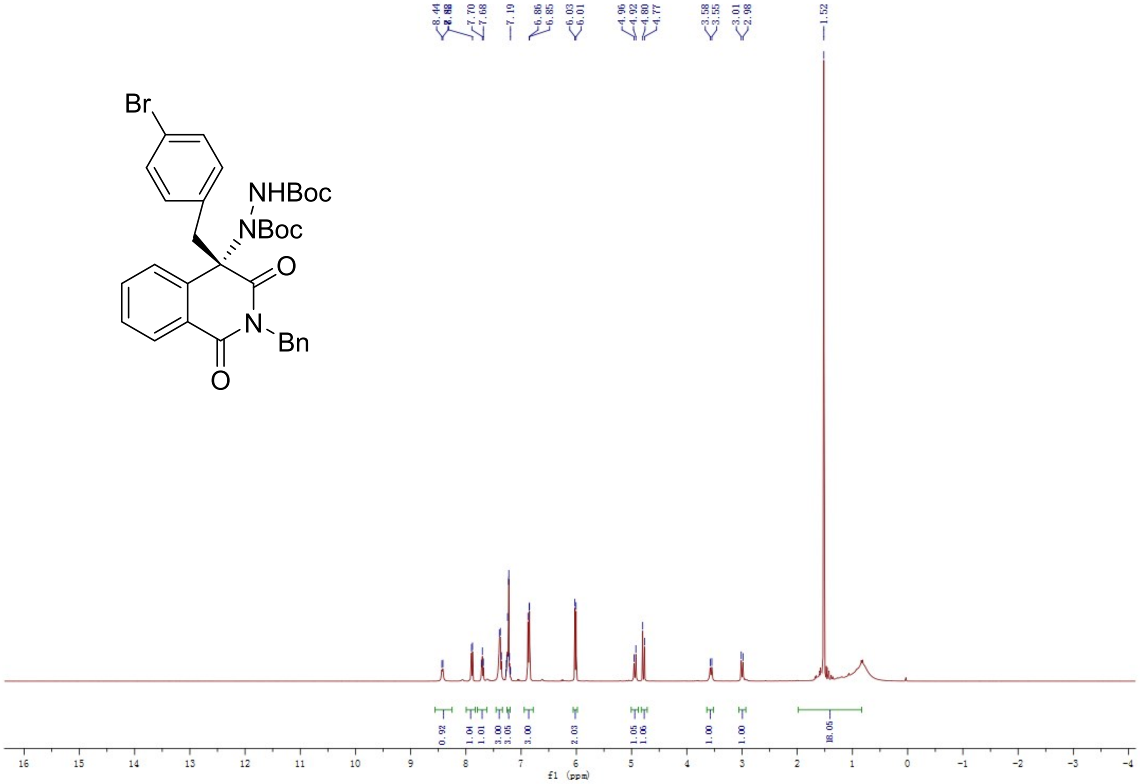

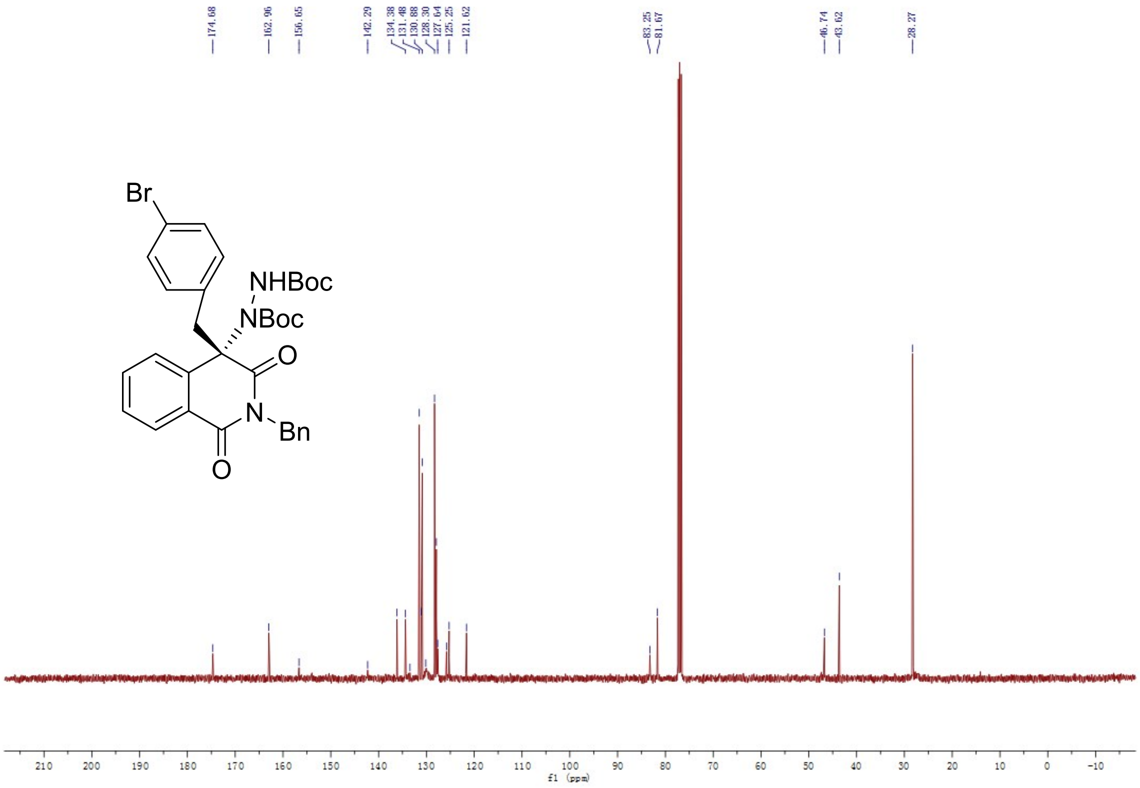

di-tert-butyl(S)-1-(2-benzyl-4-(3-methylbenzyl)-1,3-dioxo-1,2,3,4-tetrahydroisoquinolin-4-yl)hydrazine-1,2-dicarboxylate (6k):

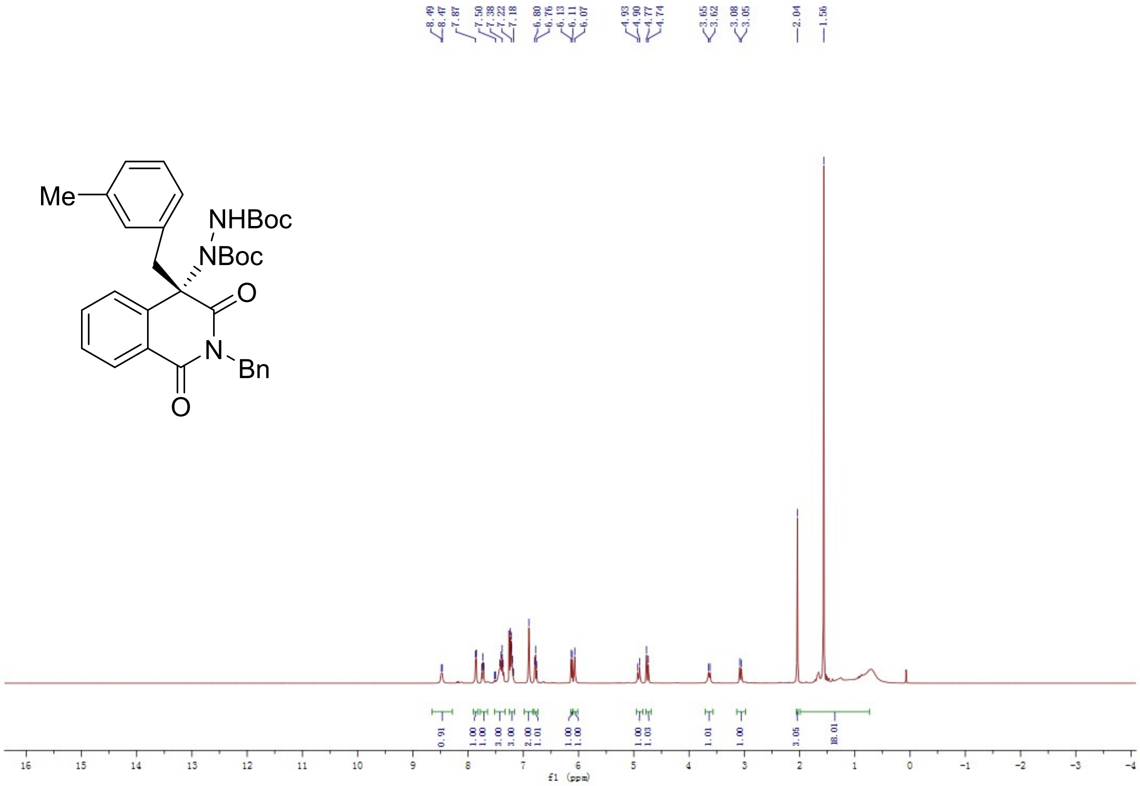

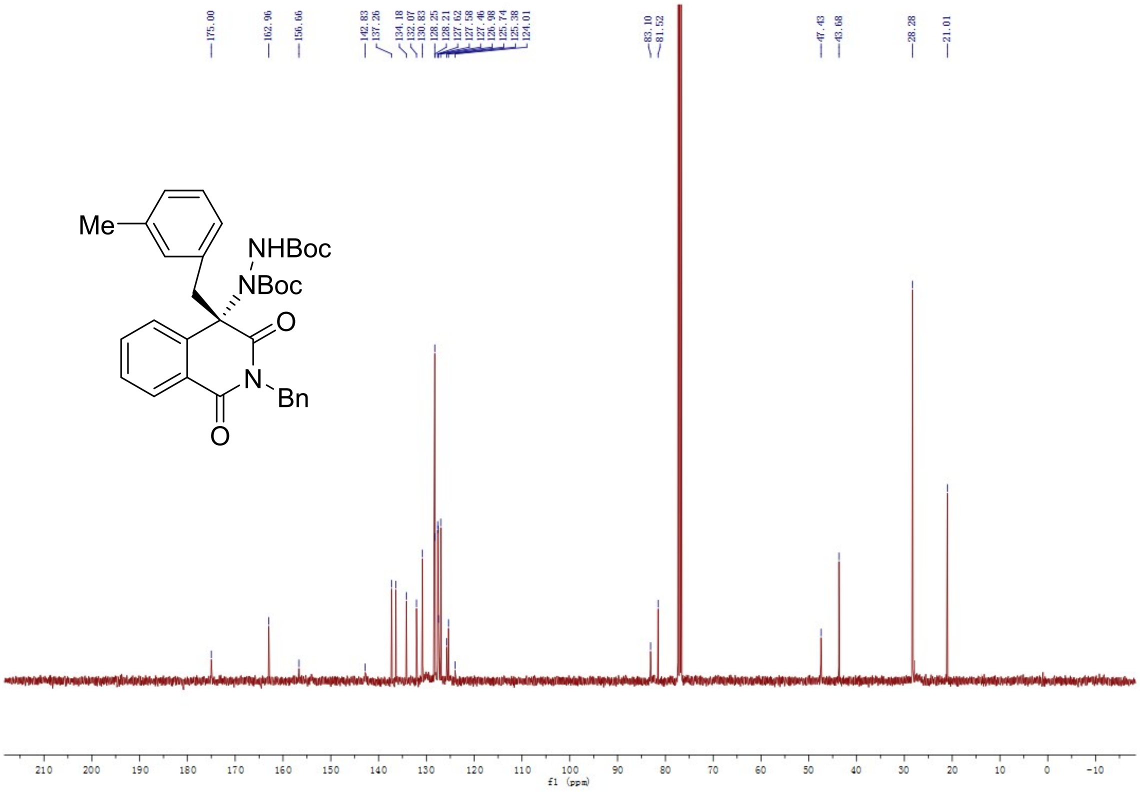

di-tert-butyl(S)-1-(2-benzyl-4-(3-bromobenzyl)-1,3-dioxo-1,2,3,4-tetrahydroisoquinolin-4-yl)hydrazine-1,2-dicarboxylate (6l):

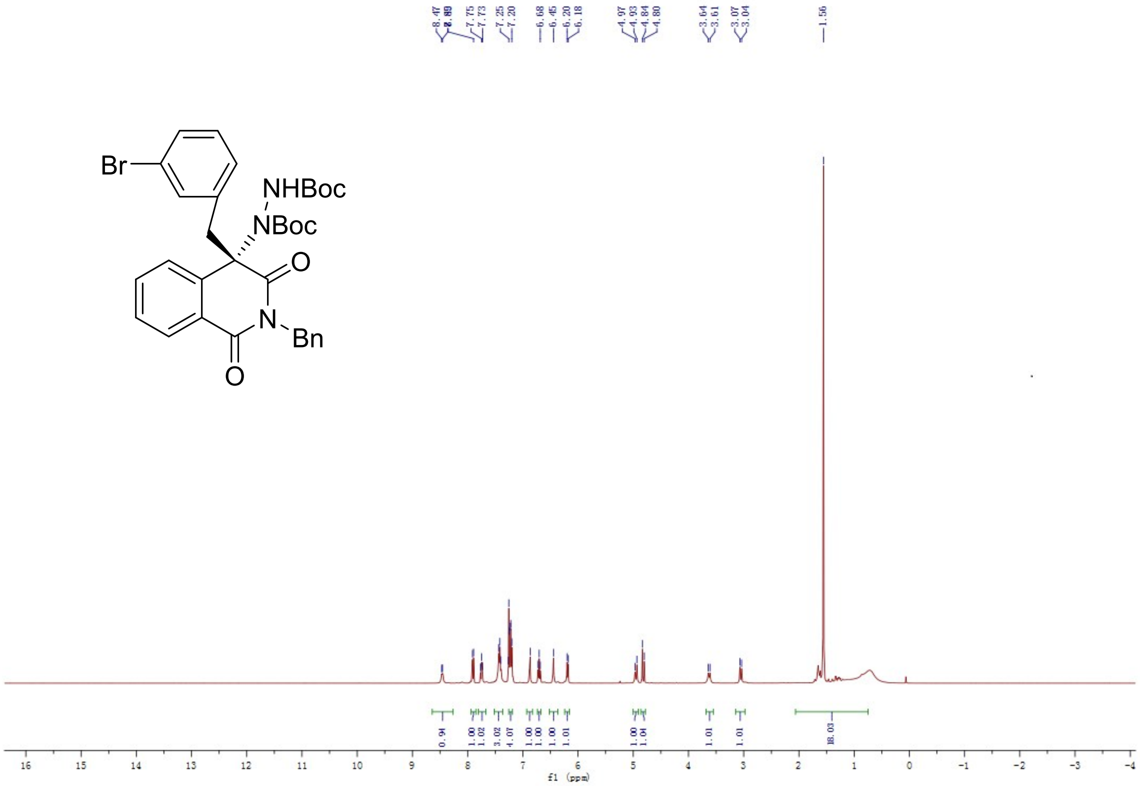

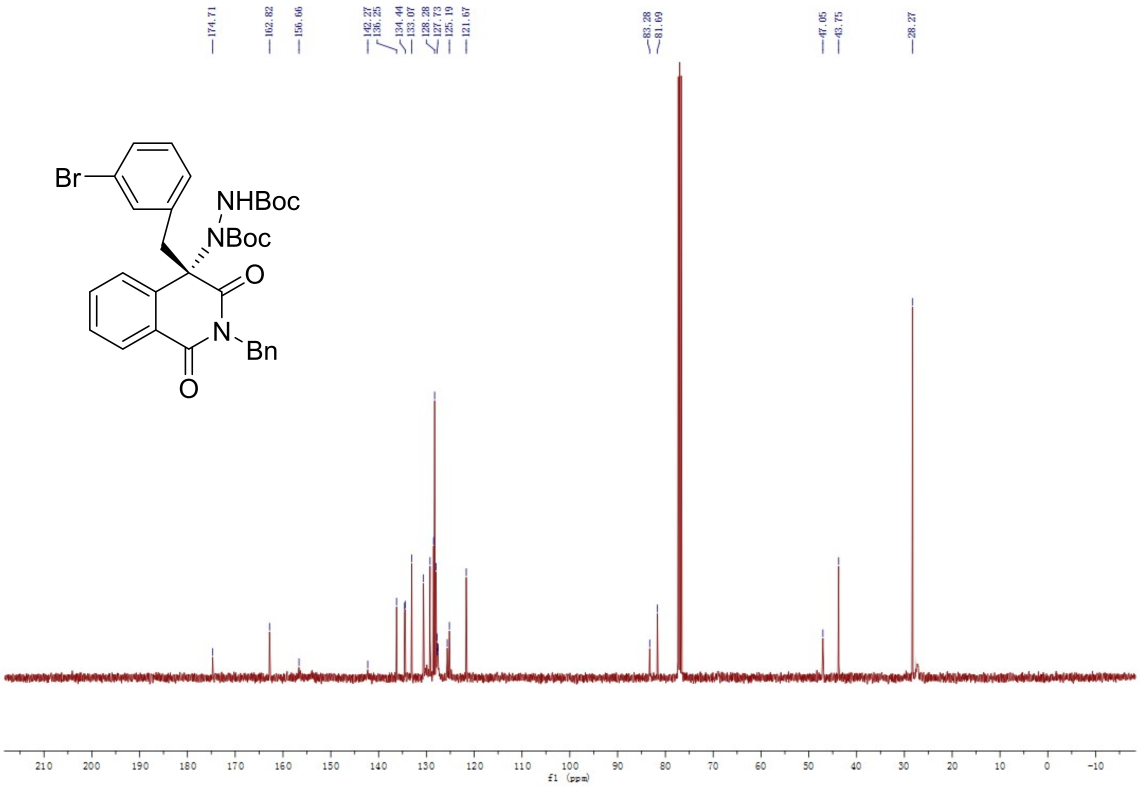

di-tert-butyl(S)-1-(2-benzyl-4-(2-methoxybenzyl)-1,3-dioxo-1,2,3,4-tetrahydroisoquinolin-4-yl)hydrazine-1,2-dicarboxylate (6m):

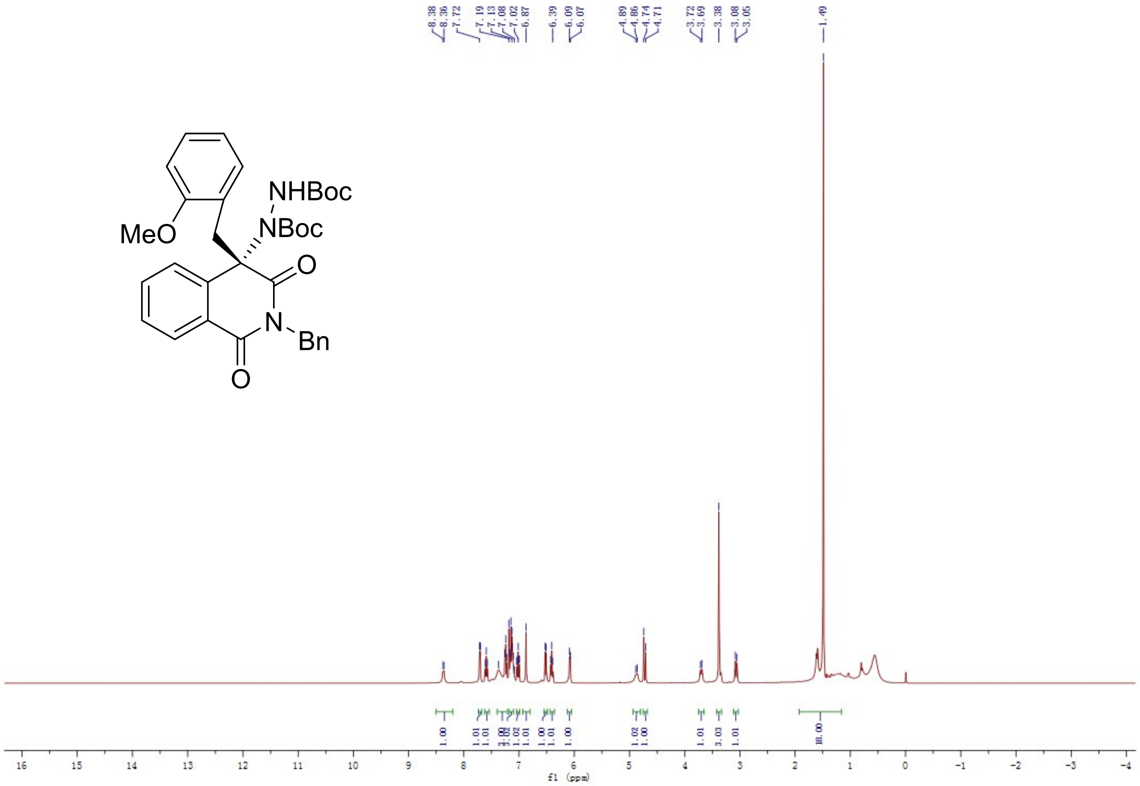

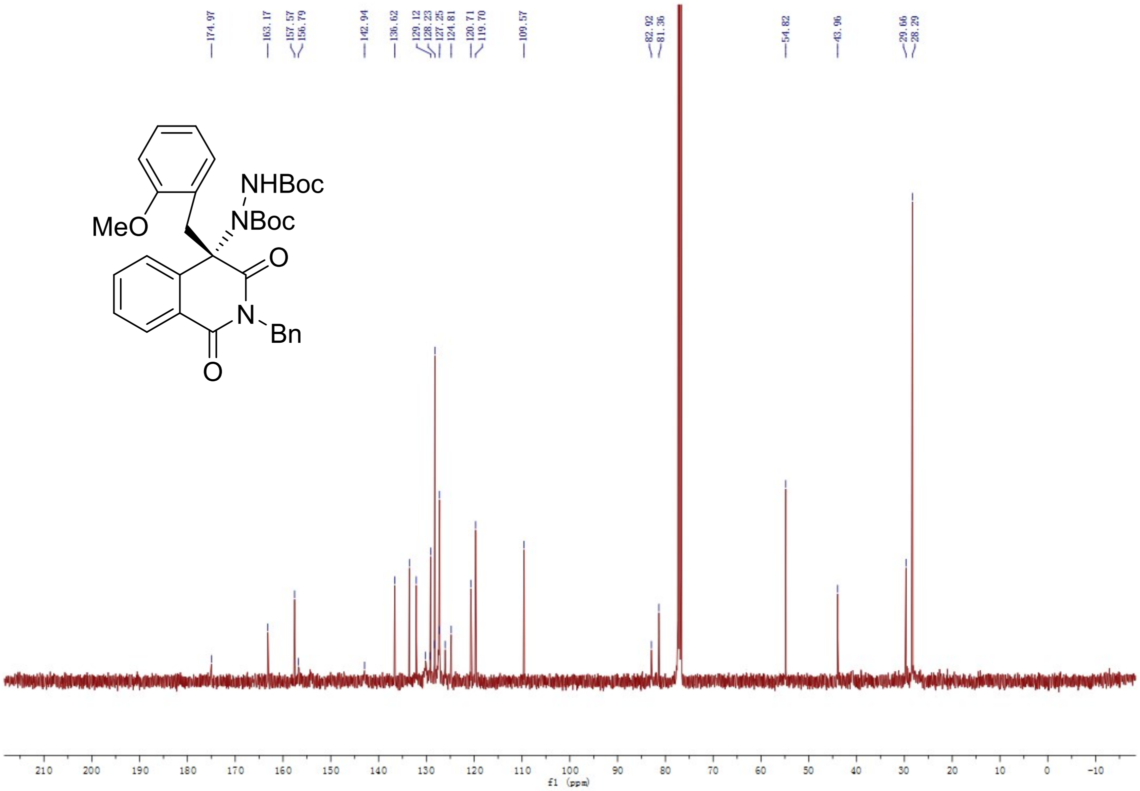

di-tert-butyl(S)-1-(2-benzyl-4-(2-methylbenzyl)-1,3-dioxo-1,2,3,4-tetrahydroisoquinolin-4-yl)hydrazine-1,2-dicarboxylate (6n):

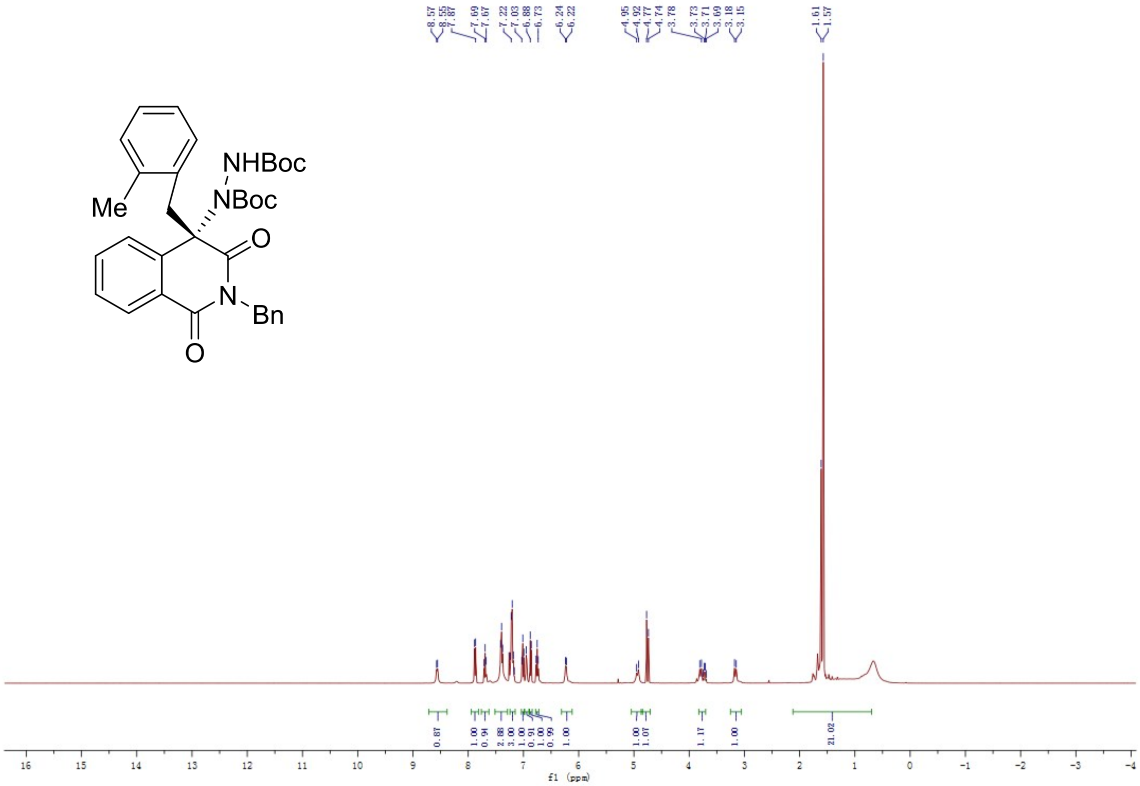

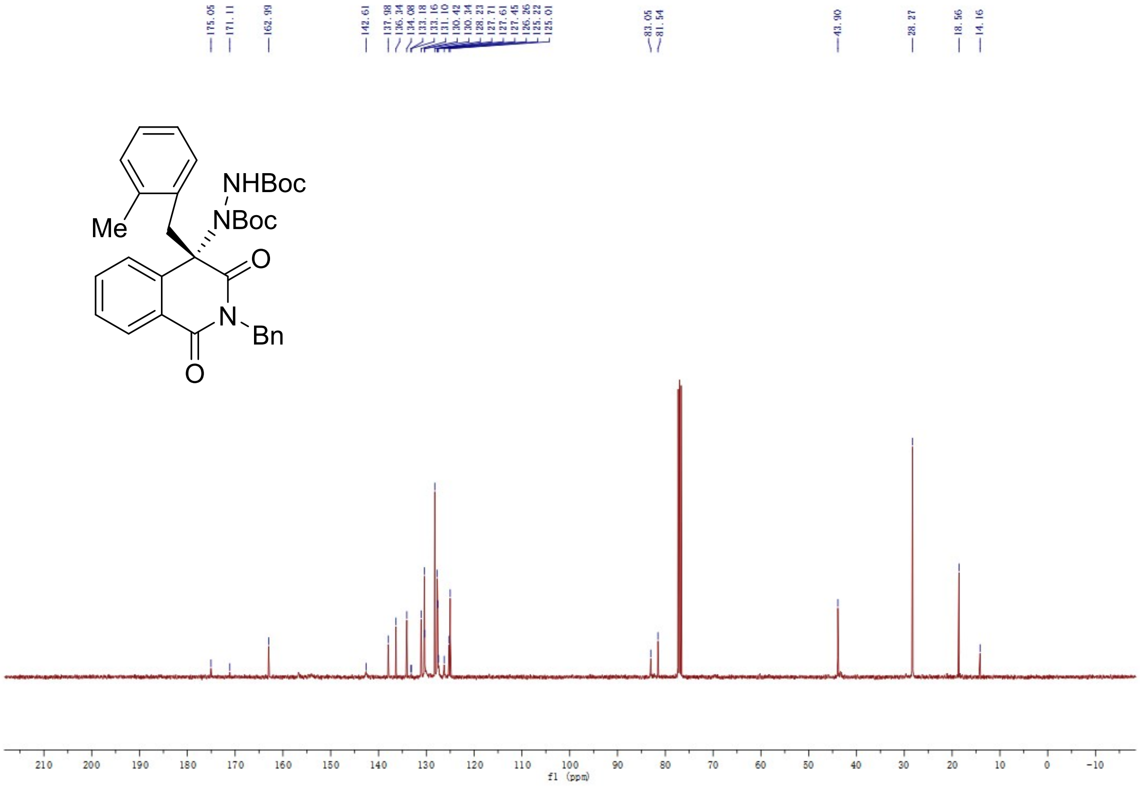

di-tert-butyl(S)-1-(2-benzyl-4-(2-chlorobenzyl)-1,3-dioxo-1,2,3,4-tetrahydroisoquinolin-4-yl)hydrazine-1,2-dicarboxylate (6o):

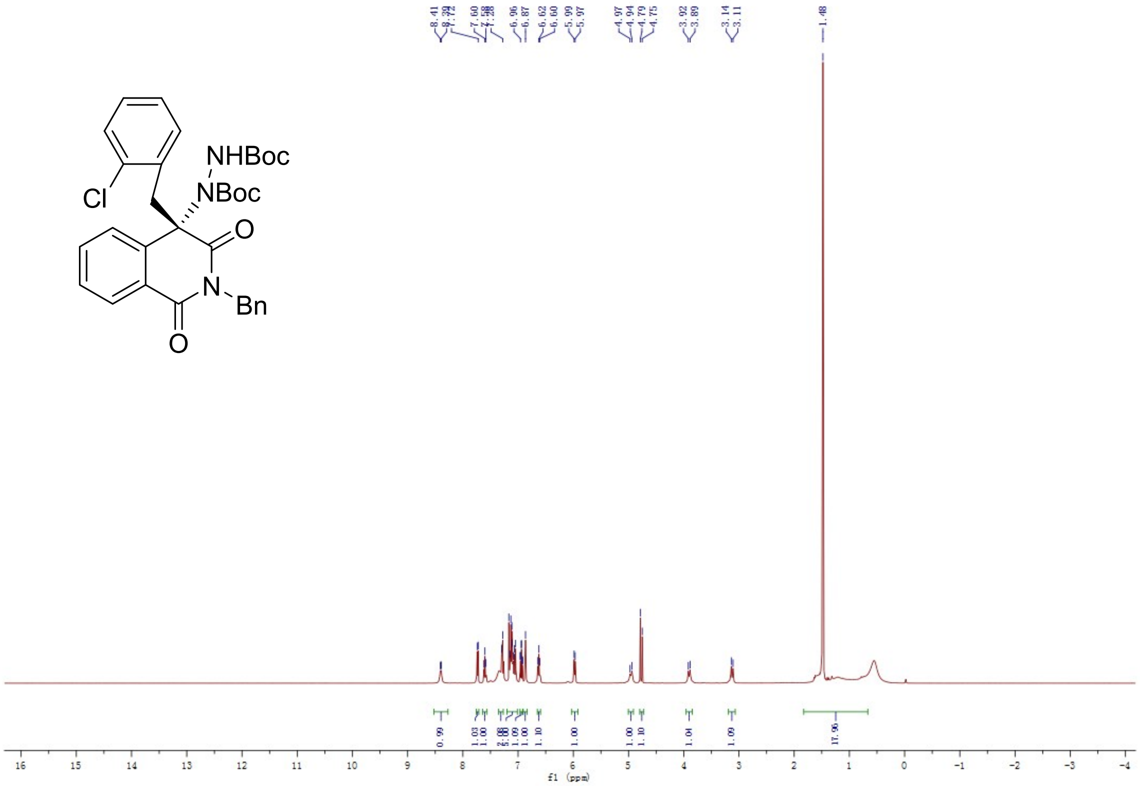

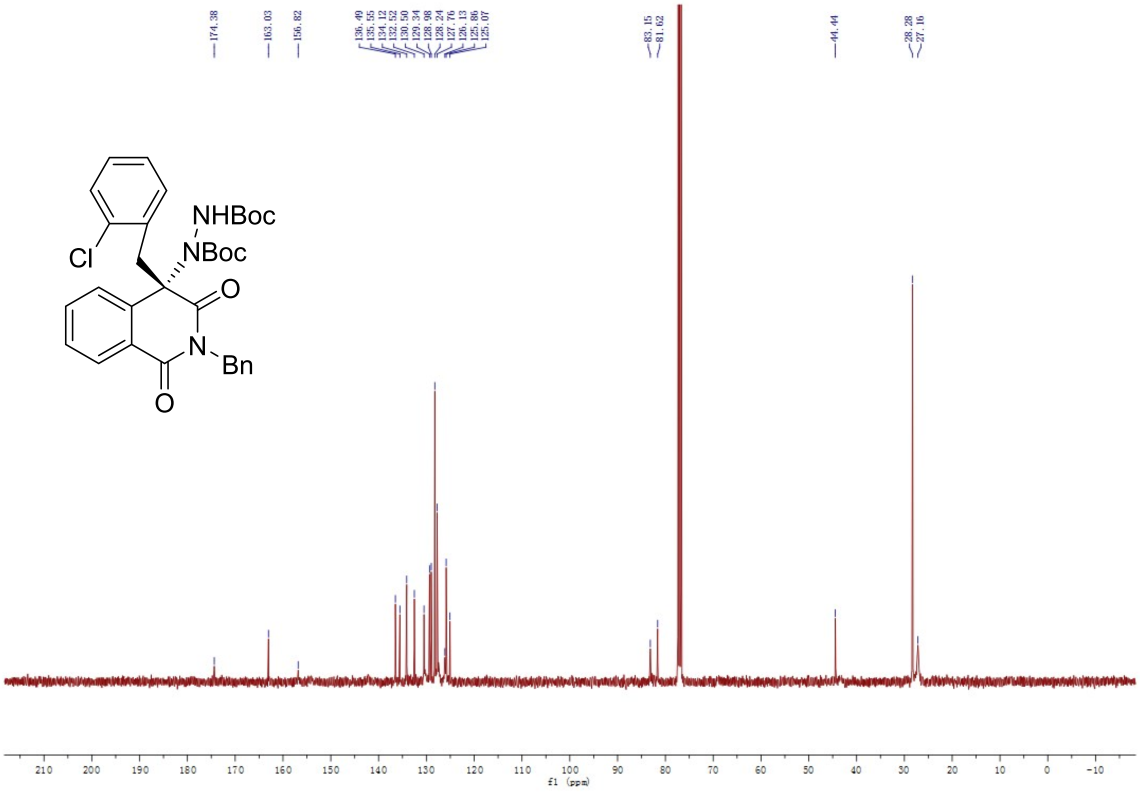

di-tert-butyl(S)-1-(2-benzyl-1,3-dioxo-4-(3,4,5-trimethoxybenzyl)-1,2,3,4-tetrahydroisoquinolin-4-yl)hydrazine-1,2-dicarboxylate (6p):

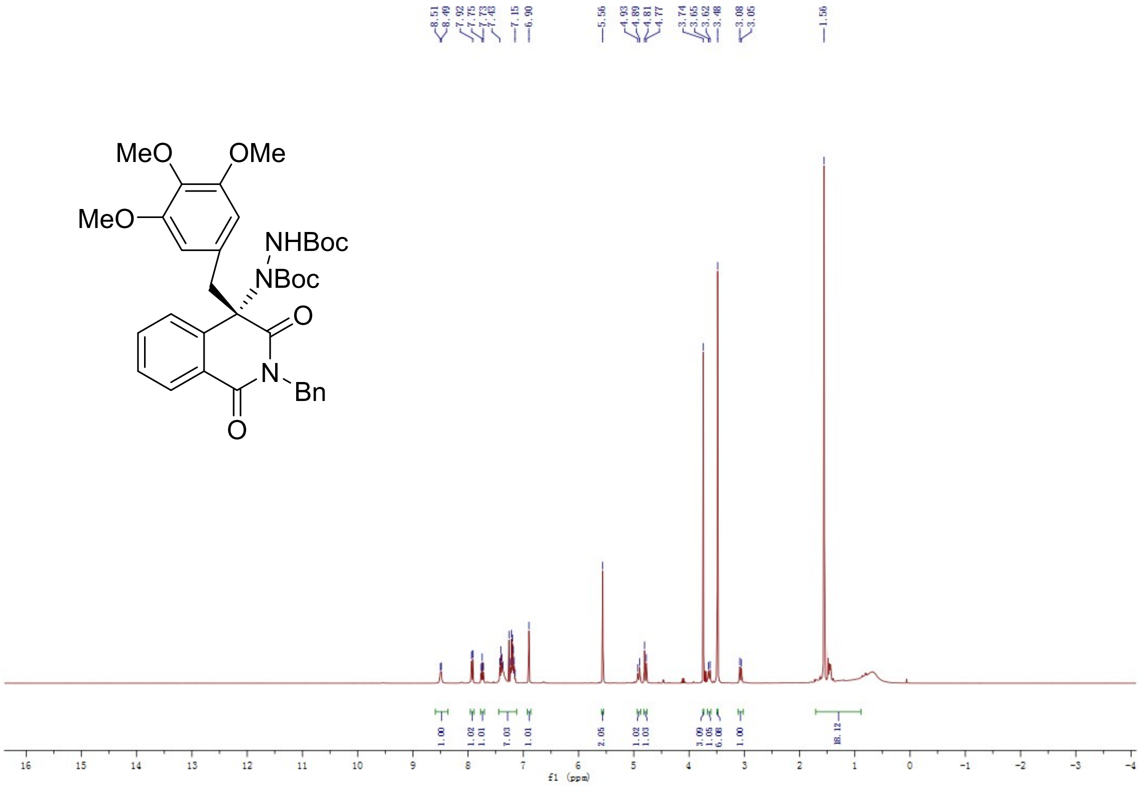

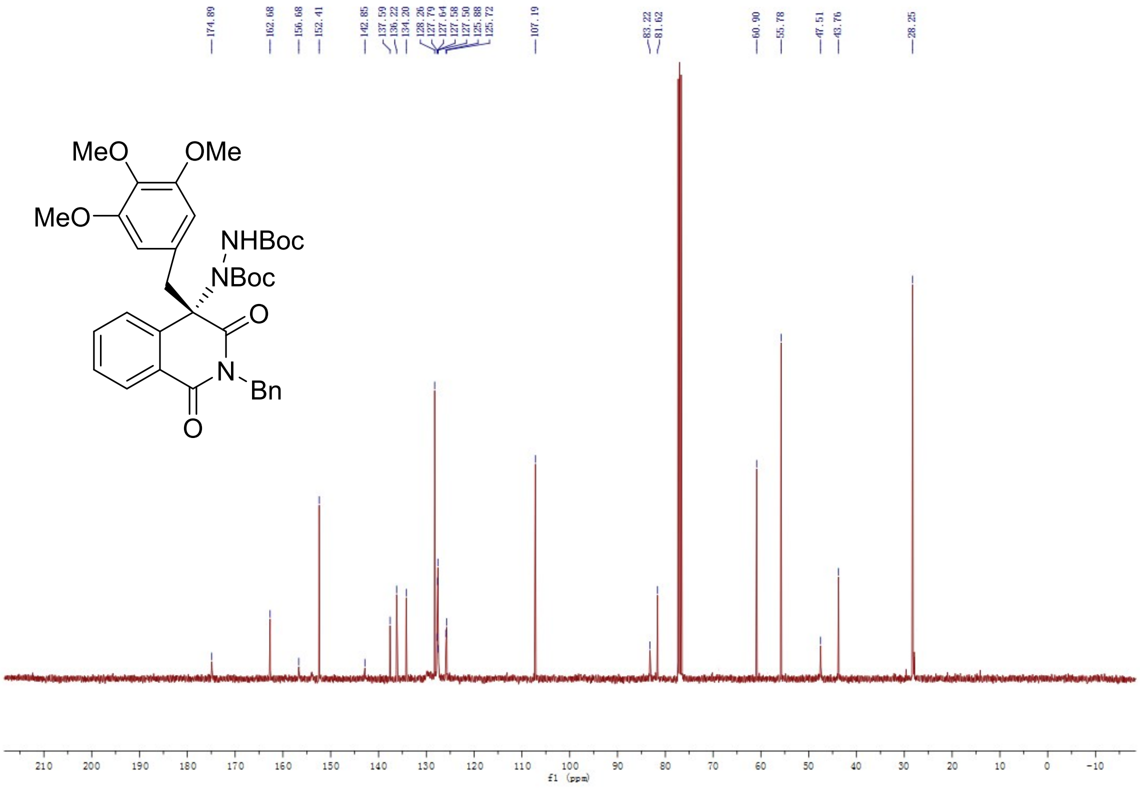

di-tert-butyl(S)-1-(2-benzyl-4-(naphthalen-2-ylmethyl)-1,3-dioxo-1,2,3,4-tetrahydroisoquinolin-4-yl)hydrazine-1,2-dicarboxylate (6q):

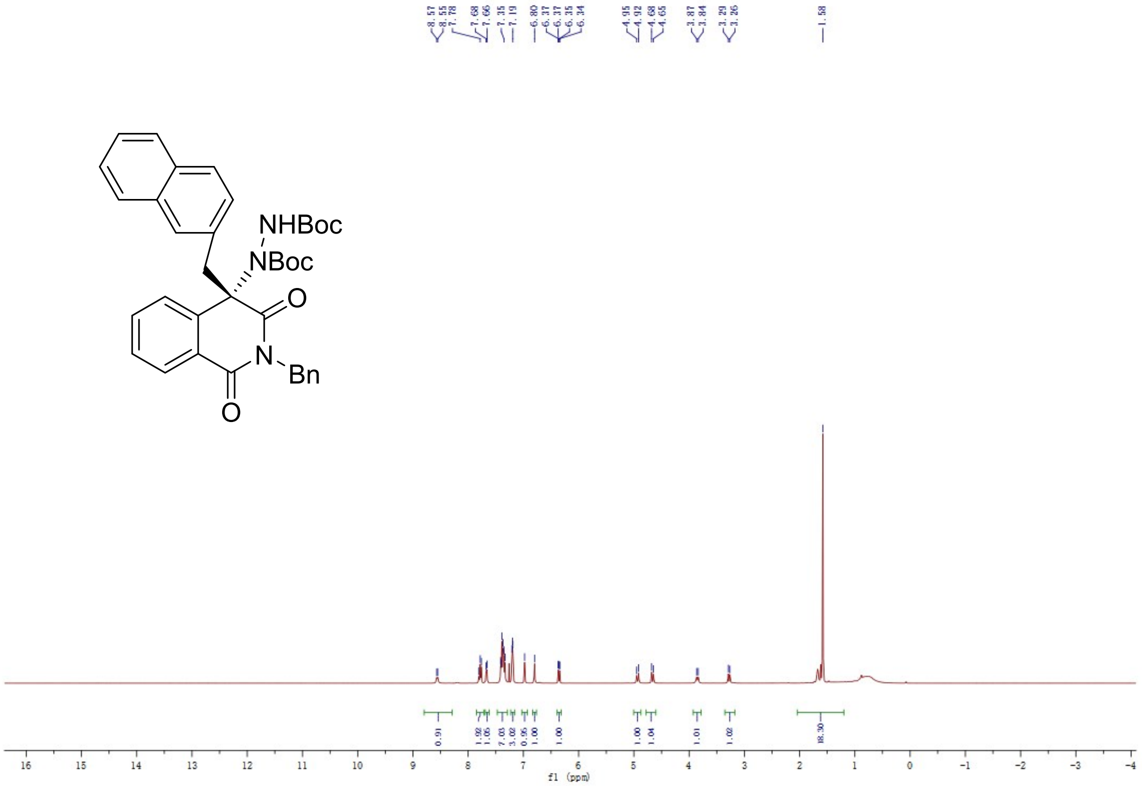

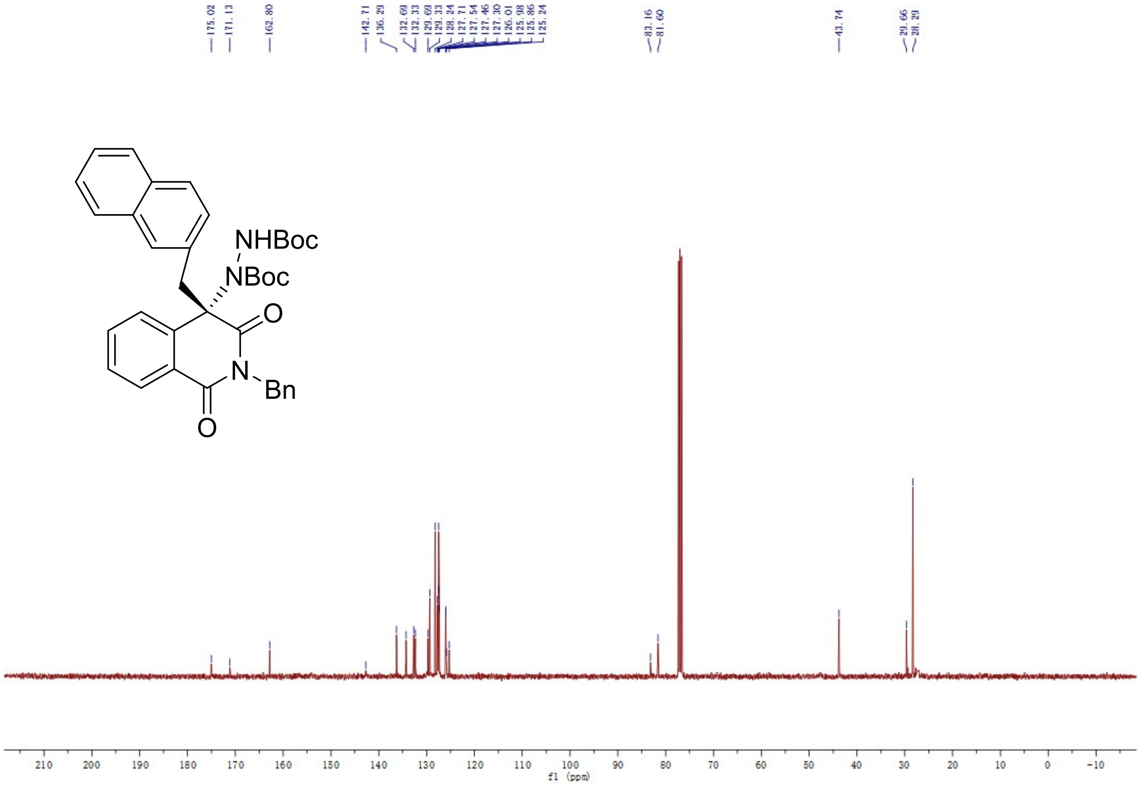

di-tert-butyl(S)-1-(4-((1H-indol-2-yl)methyl)-2-benzyl-1,3-dioxo-1,2,3,4-tetrahydroisoquinolin-4-yl)hydrazine-1,2-dicarboxylate (6r):

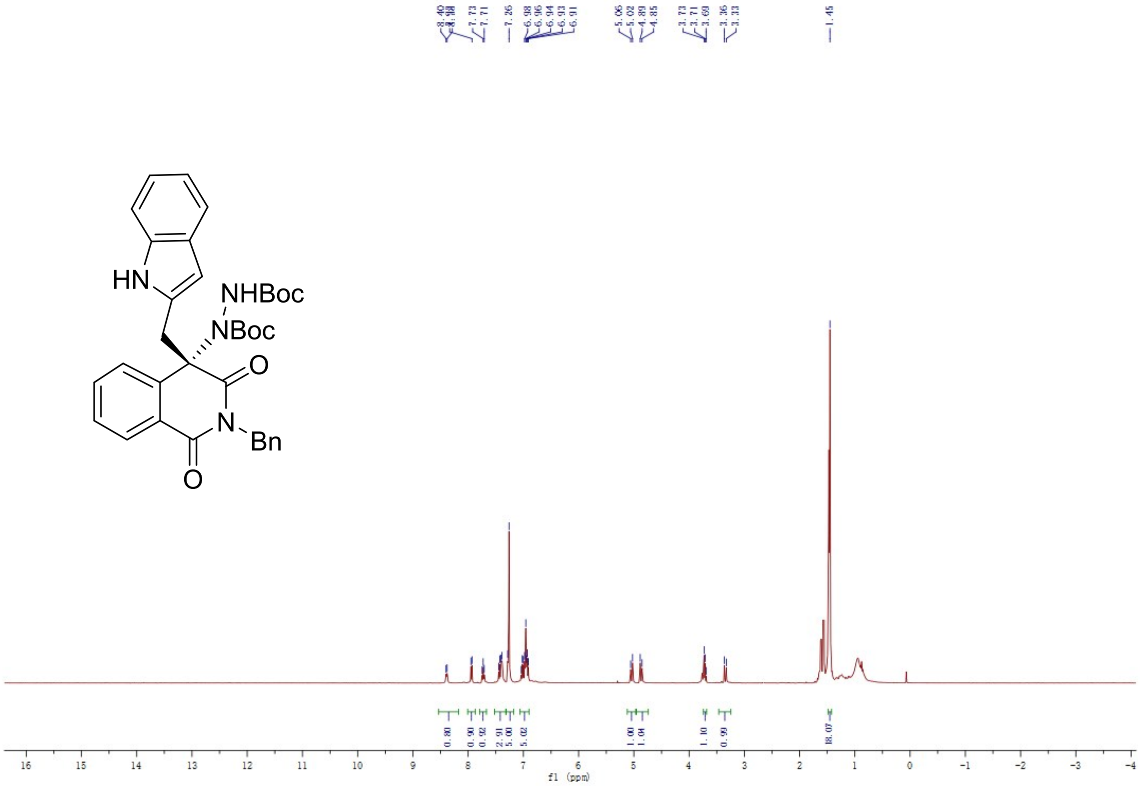

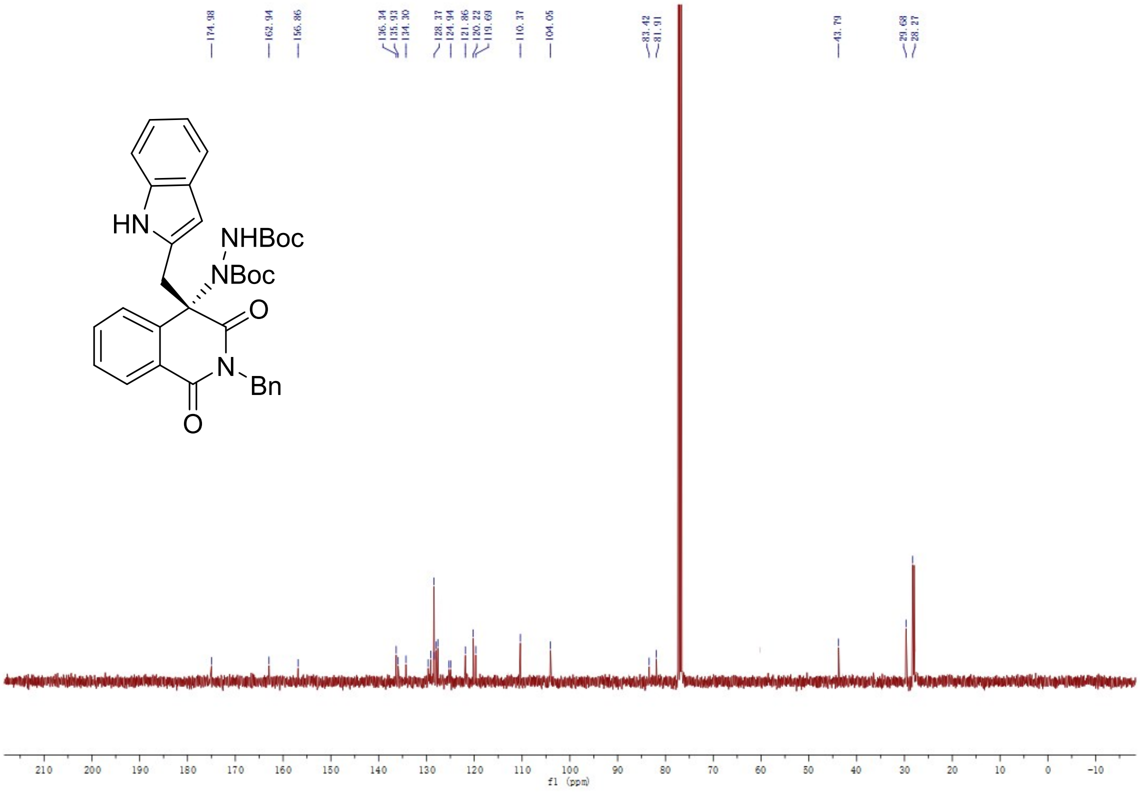

di-tert-butyl(S)-1-(4-((1H-indol-3-yl)methyl)-2-benzyl-1,3-dioxo-1,2,3,4-tetrahydroisoquinolin-4-yl)hydrazine-1,2-dicarboxylate (6s):

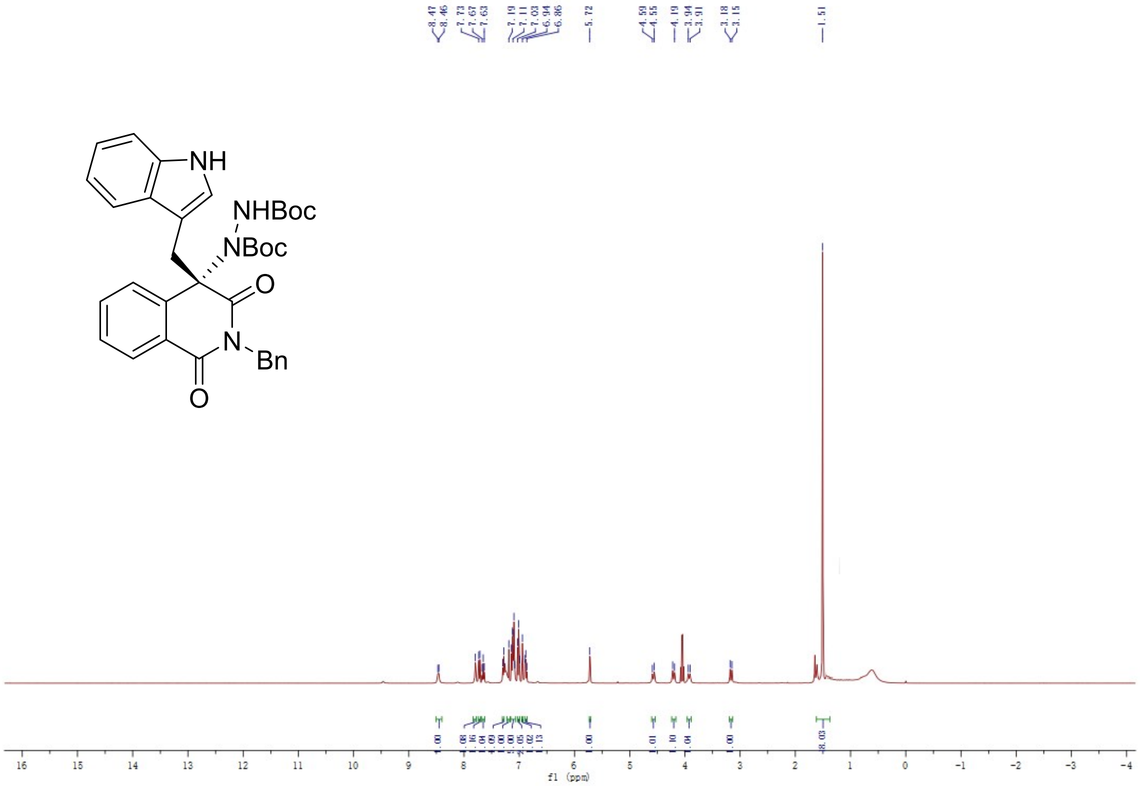

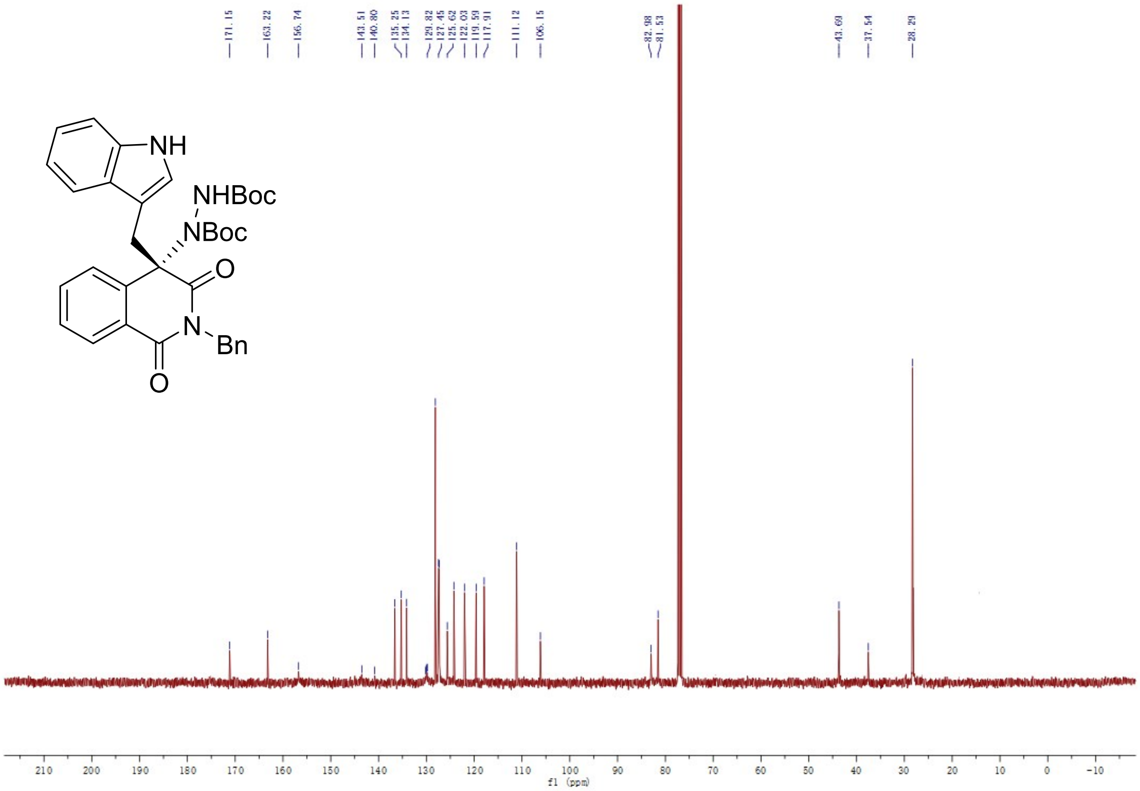

di-tert-butyl(S)-1-(2-benzyl-4-((2-methyl-1H-indol-3-yl)methyl)-1,3-dioxo-1,2,3,4-tetrahydroisoquinolin-4-yl)hydrazine-1,2-dicarboxylate (6t):

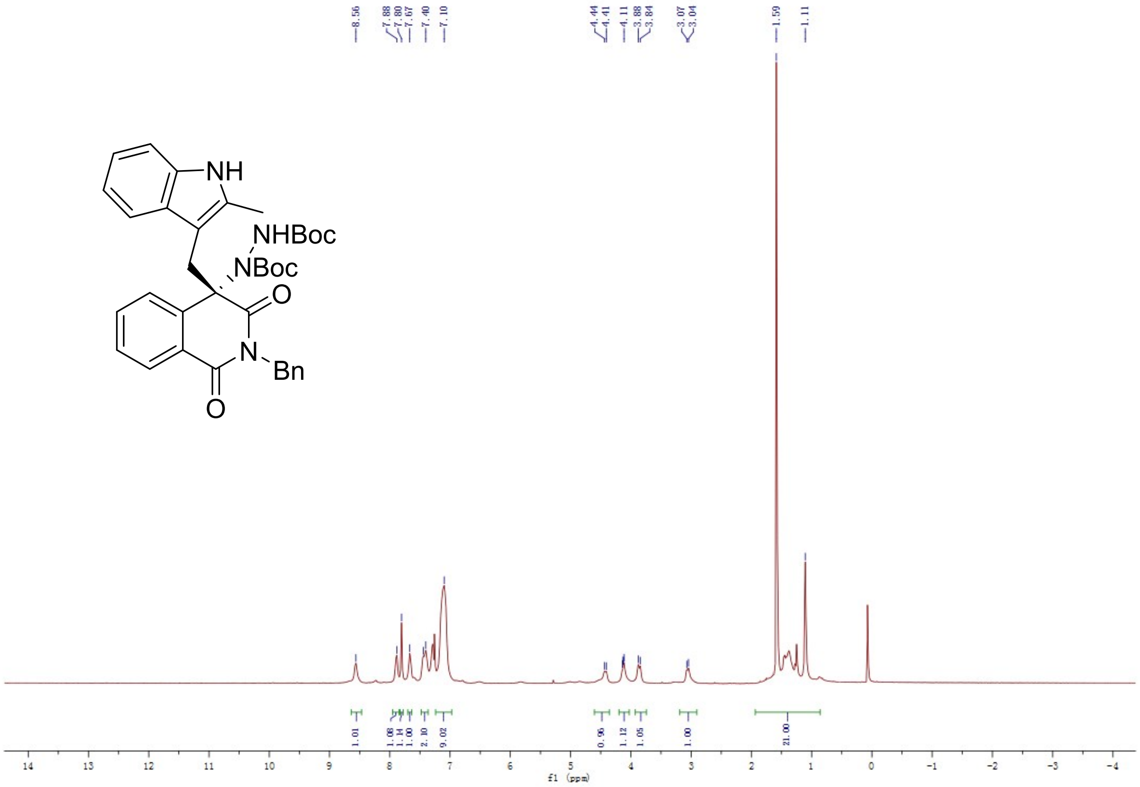

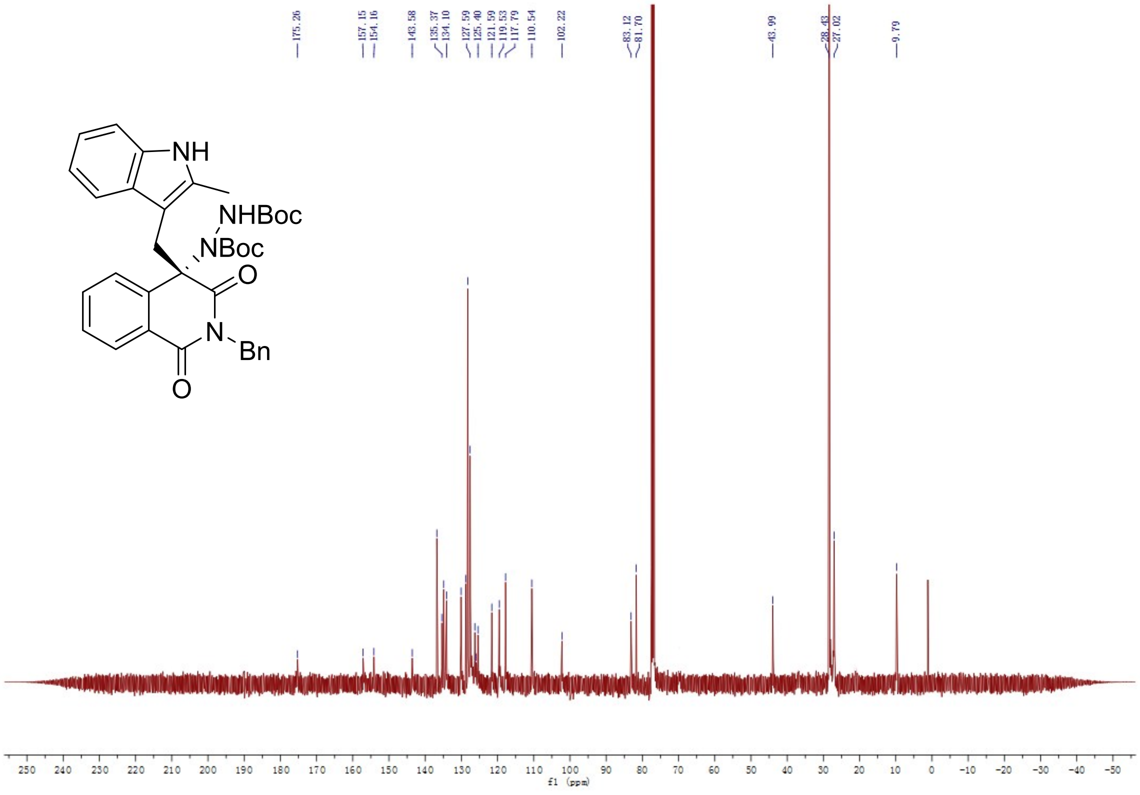

di-tert-butyl(S)-1-(2-benzyl-4-(furan-2-ylmethyl)-1,3-dioxo-1,2,3,4-tetrahydroisoquinolin-4-yl)hydrazine-1,2-dicarboxylate (6u):

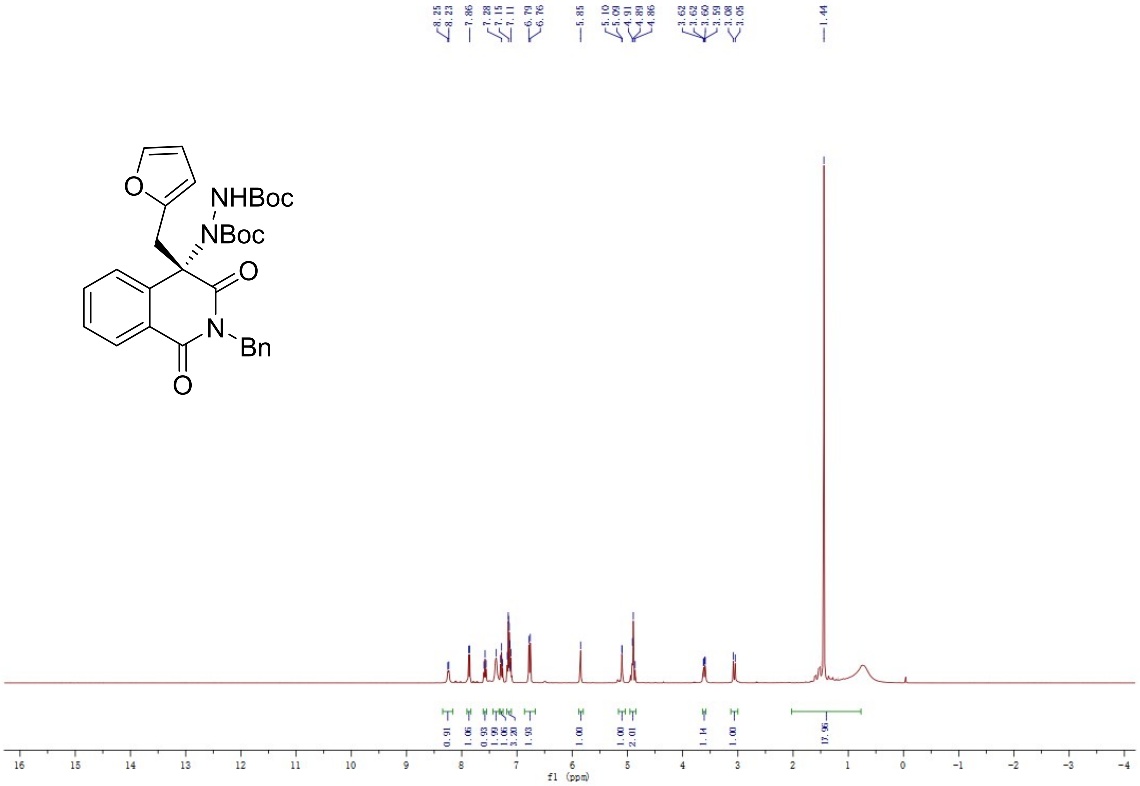

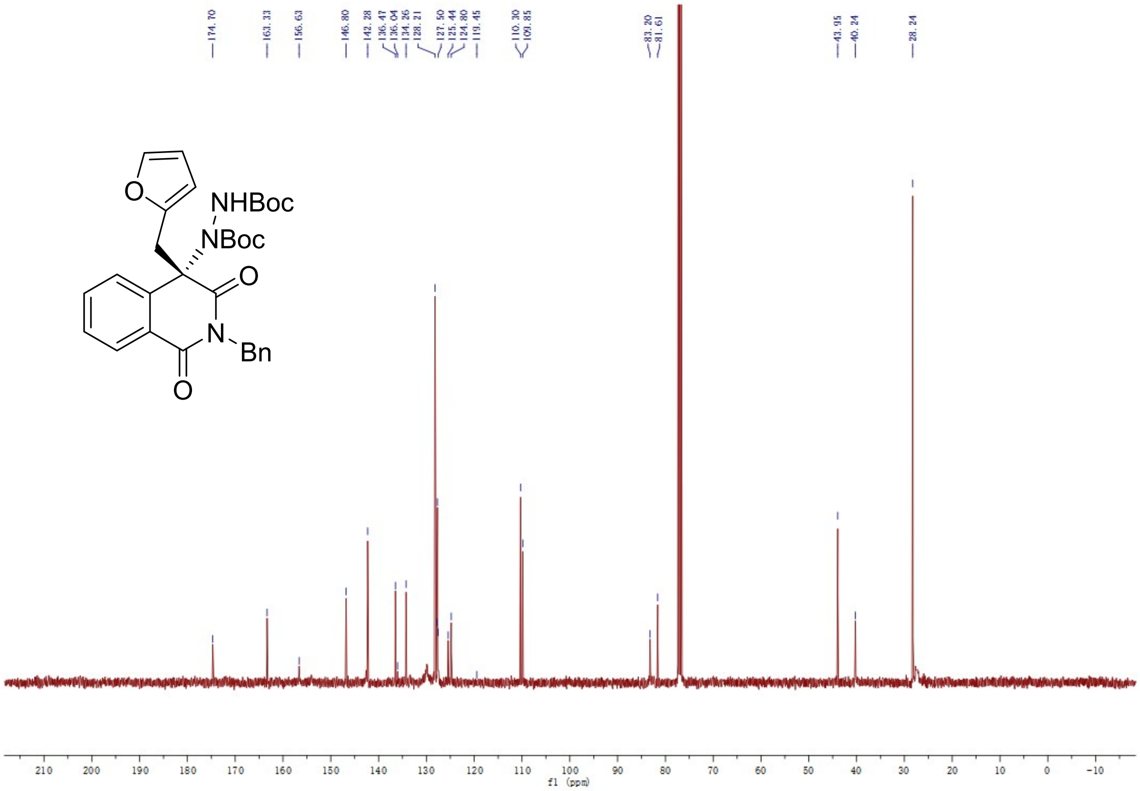

diethyl (S)-1-(2-benzyl-4-butyl-1,3-dioxo-1,2,3,4-tetrahydroisoquinolin-4-yl)hydrazine-1,2-dicarboxylate (6v):

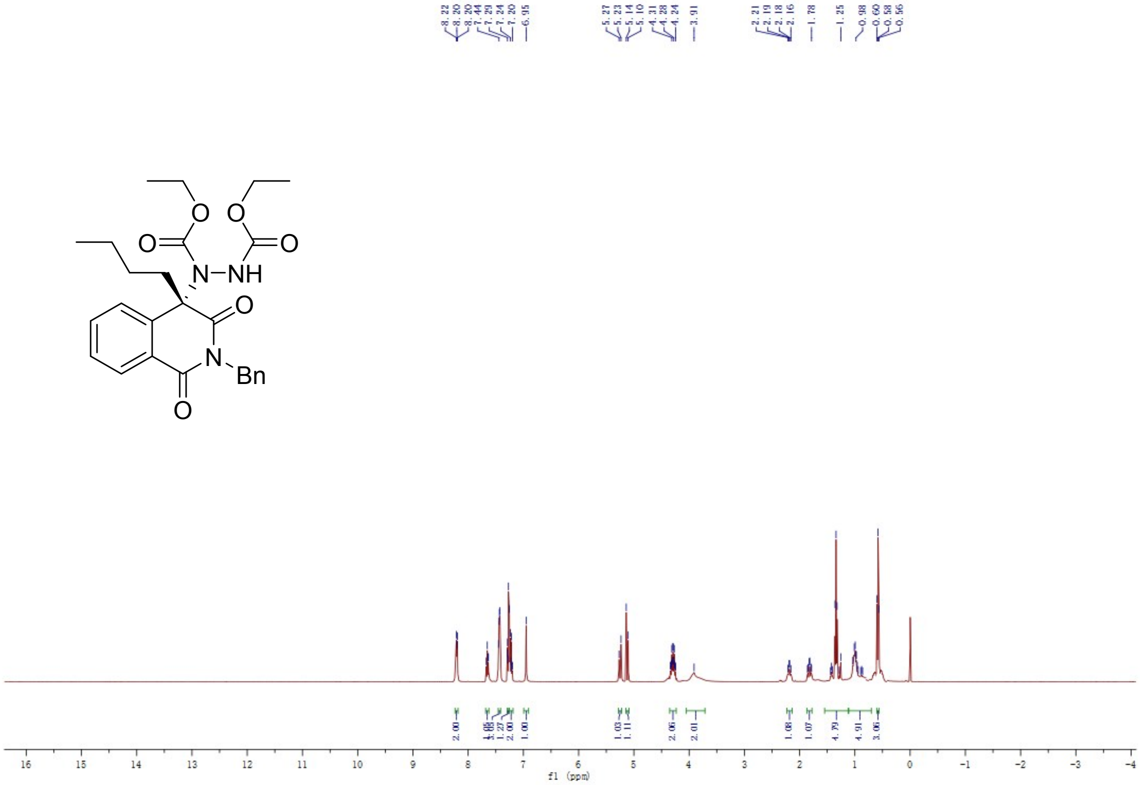

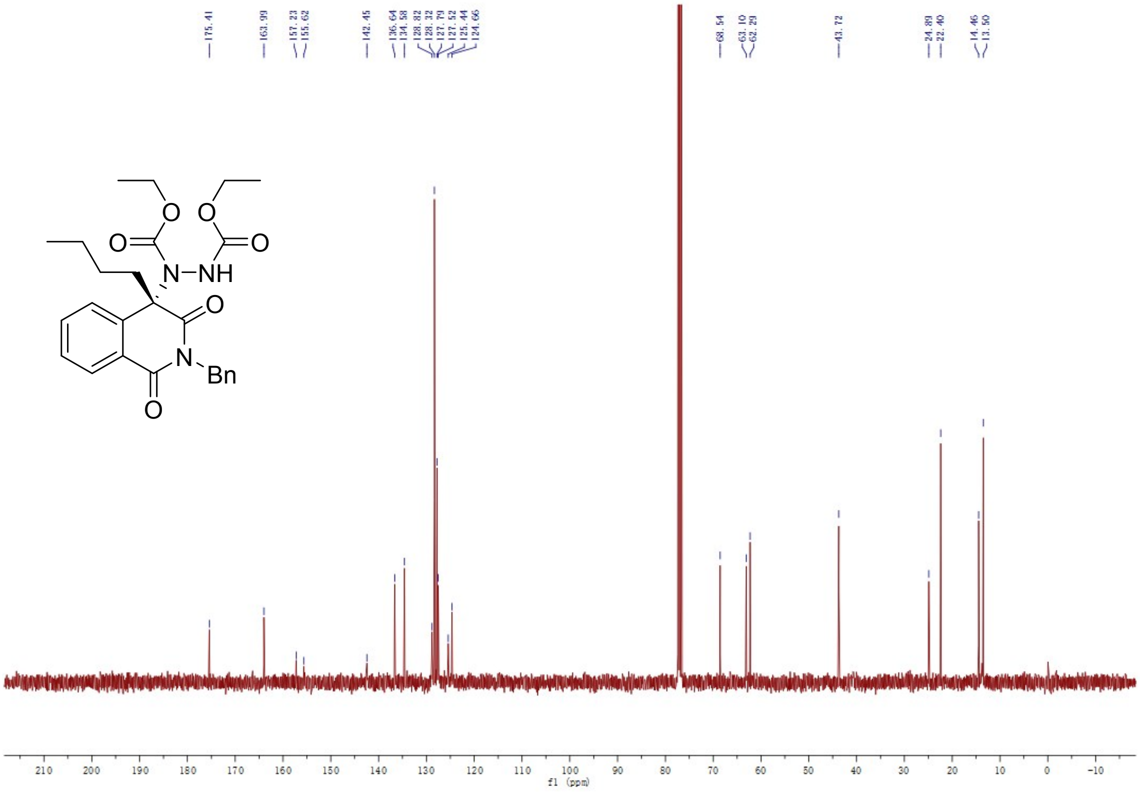

diisopropyl (S)-1-(2-benzyl-4-butyl-1,3-dioxo-1,2,3,4-tetrahydroisoquinolin-4-yl)hydrazine-1,2-dicarboxylate (6w):

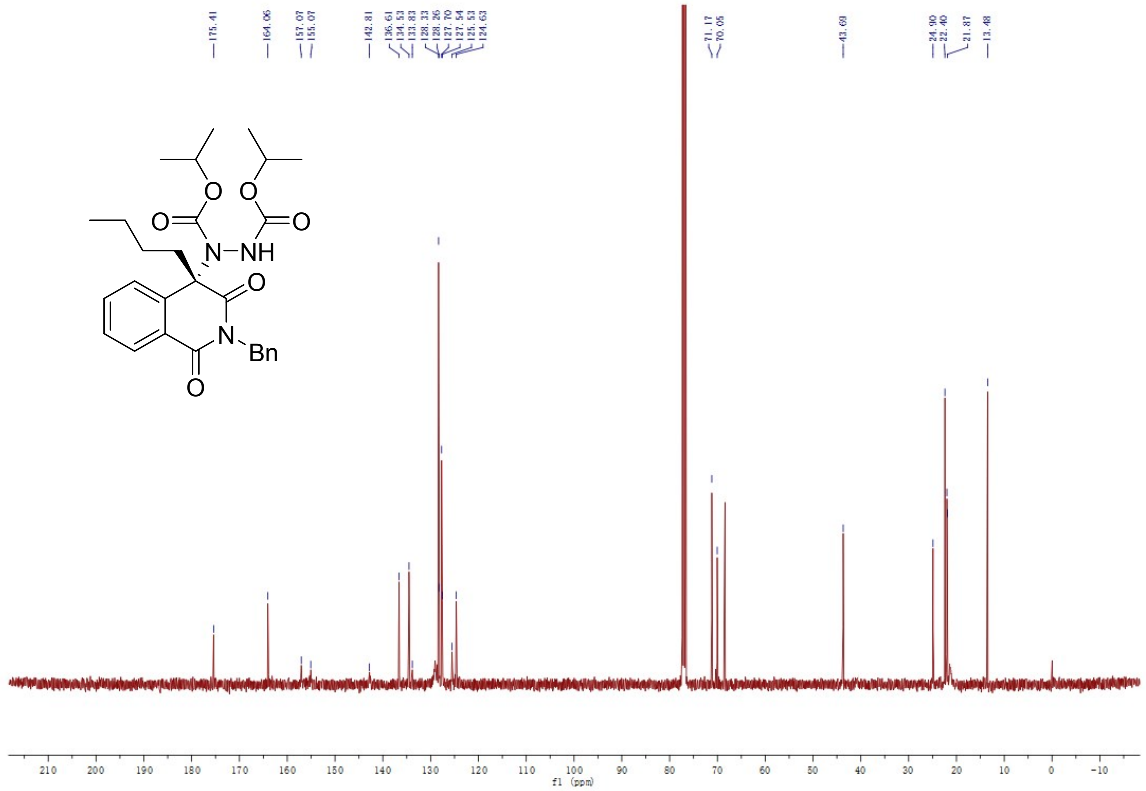

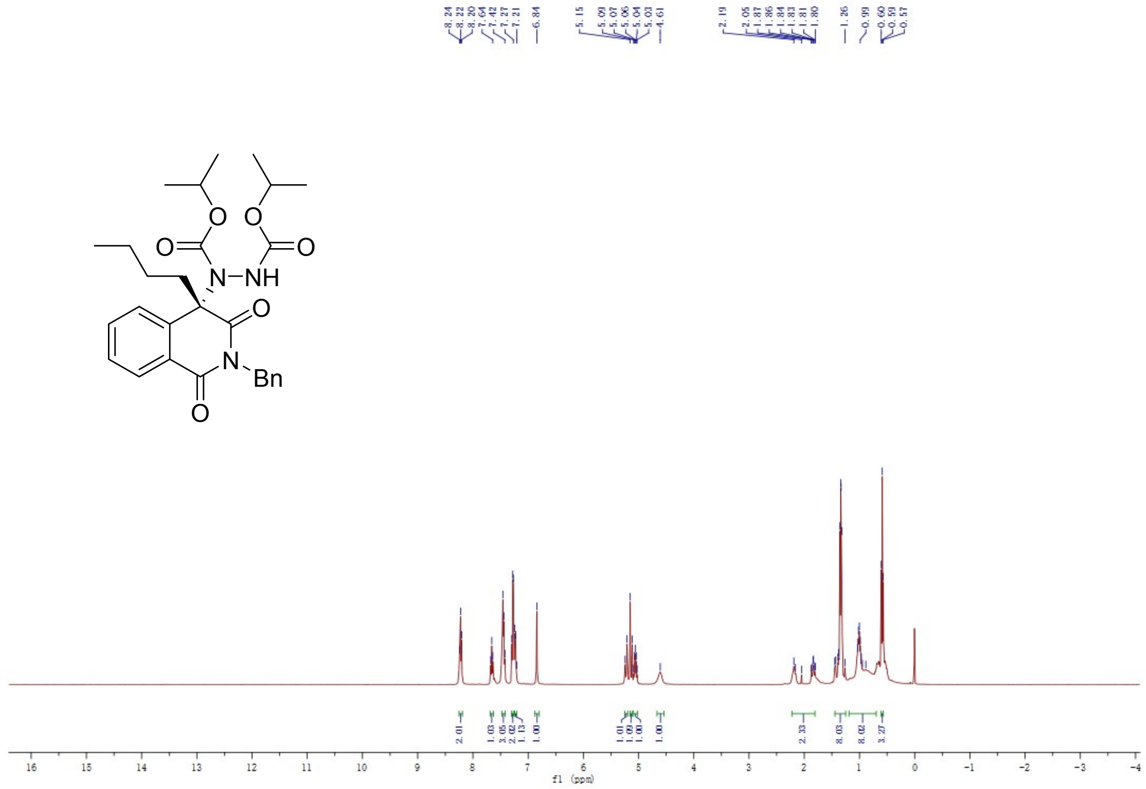

(R)-N-benzyl-1-(4-chlorobenzyl)-3-oxoisoindoline-1-carboxamide (7):

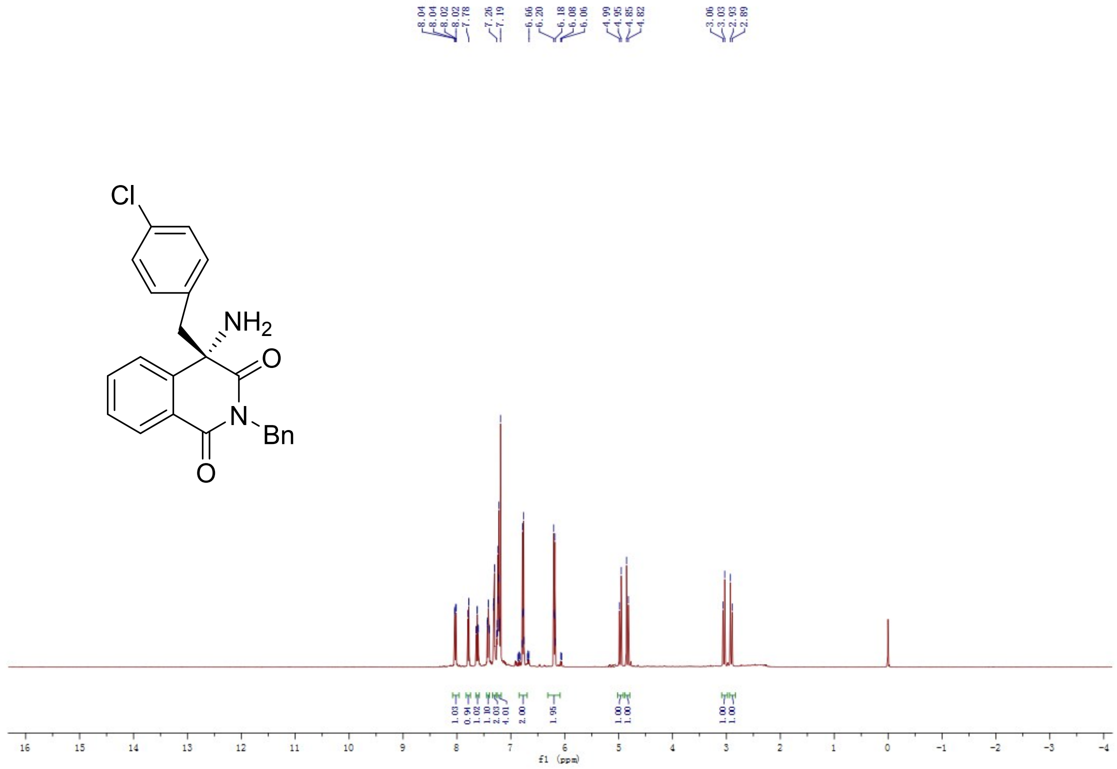

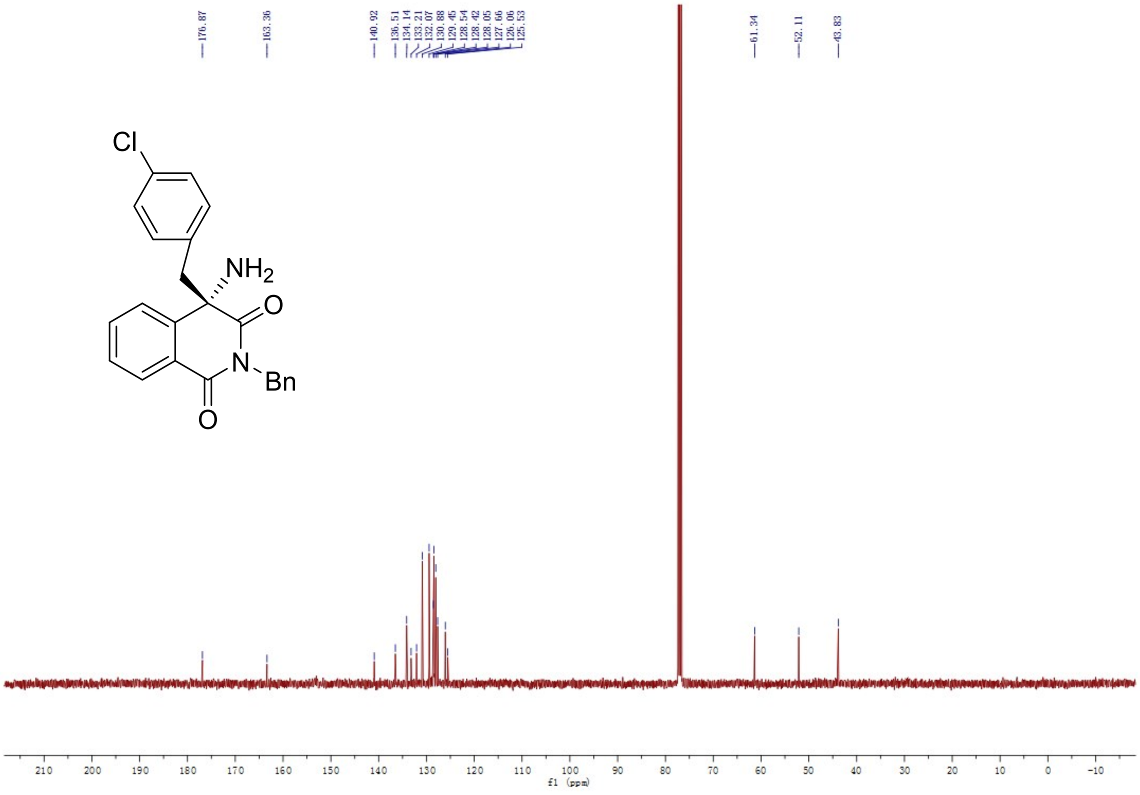

9. HPLC spectra

di-tert-butyl(S)-1-(2-benzyl-4-butyl-1,3-dioxo-1,2,3,4-tetrahydroisoquinolin-4-yl)hydrazine-1,2-dicarboxylate (6a):

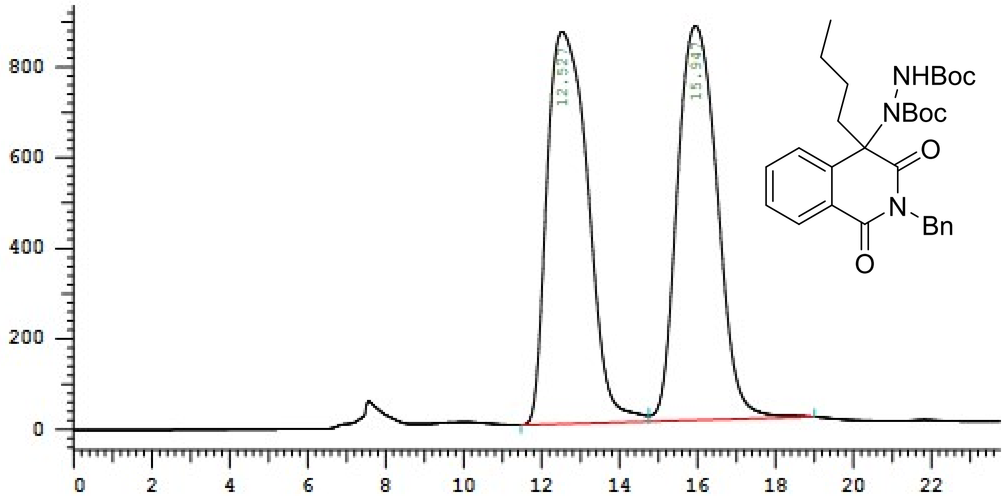

No.	RT	面积	浓度1	BC
1	12.527	63101527	50.441	BV
2	15.947	61998018	49.559	VB
		1.250E+08	100.000	

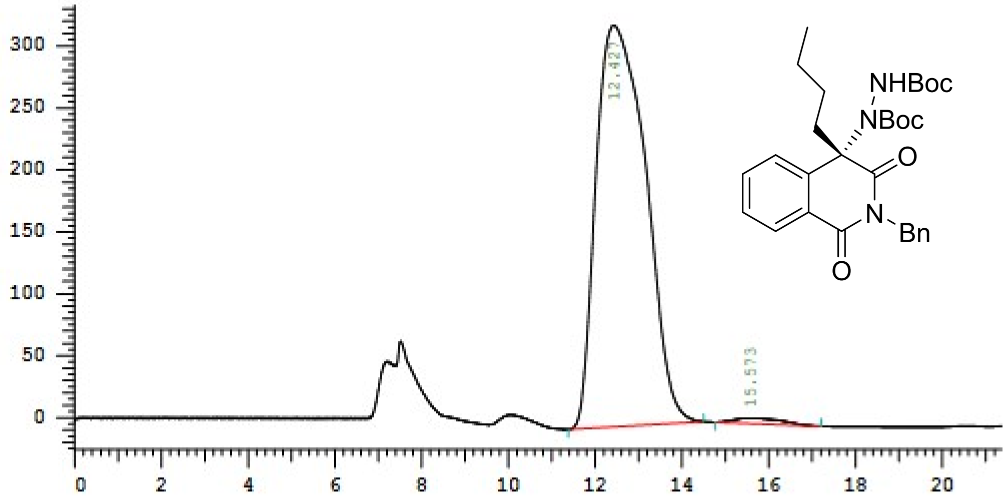

No.	RT	面积	浓度1	BC
1	12.427	25759040	98.699	BB
2	15.573	339638	1.301	BB
		26098678	100.000	

di-tert-butyl(S)-1-(2-benzyl-4-isopentyl-1,3-dioxo-1,2,3,4-tetrahydroisoquinolin-4-yl)hydrazine-1,2-dicarboxylate (6b):

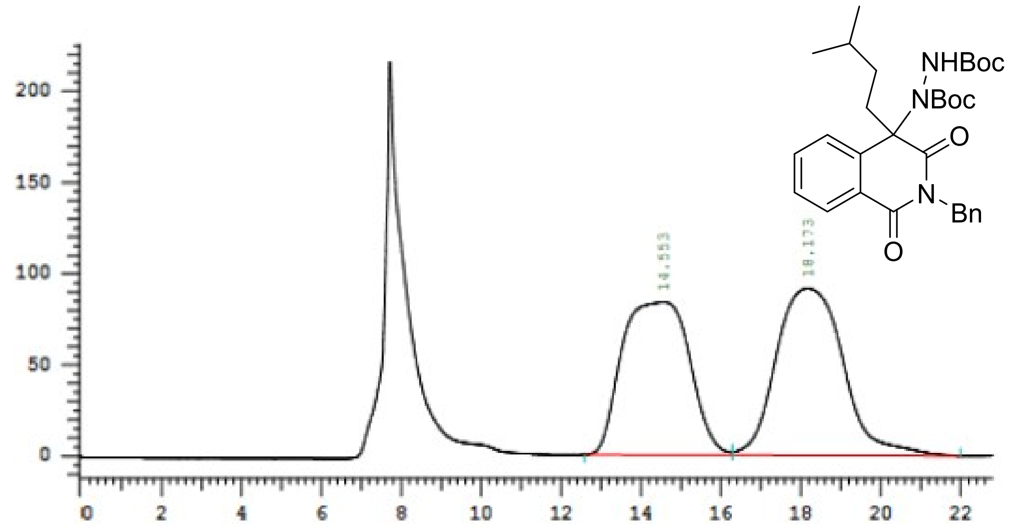

No.	RT	面积	浓度1	BC
1	14.553	2146759	48.034	BV
2	18.173	2322505	51.966	VB
		4469264	100.000	

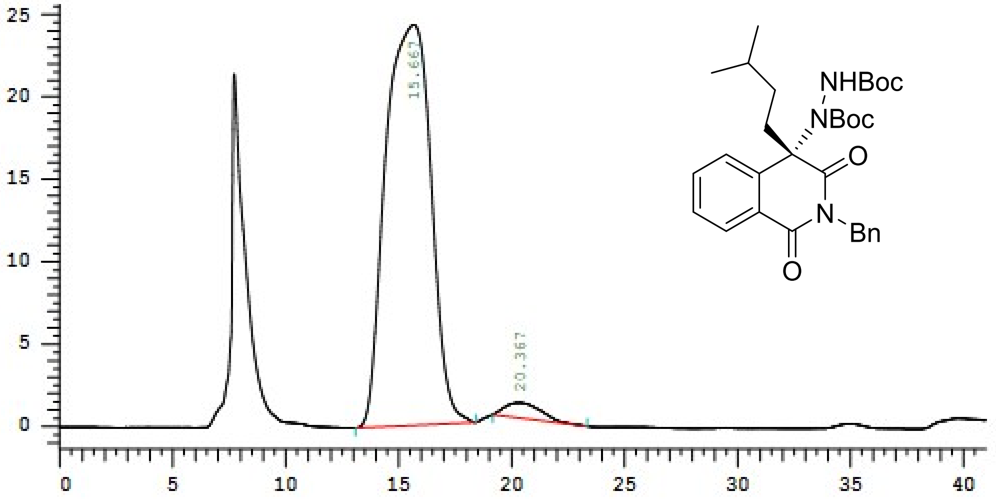

No.	RT	面积	浓度1	BC
1	15.667	3390546	97.034	BB
2	20.367	103623	2.966	BB
		3494169	100.000	

di-tert-butyl(S)-1-(2-benzyl-4-isobutyl-1,3-dioxo-1,2,3,4-tetrahydroisoquinolin-4-yl)hydrazine-1,2-dicarboxylate (6c):

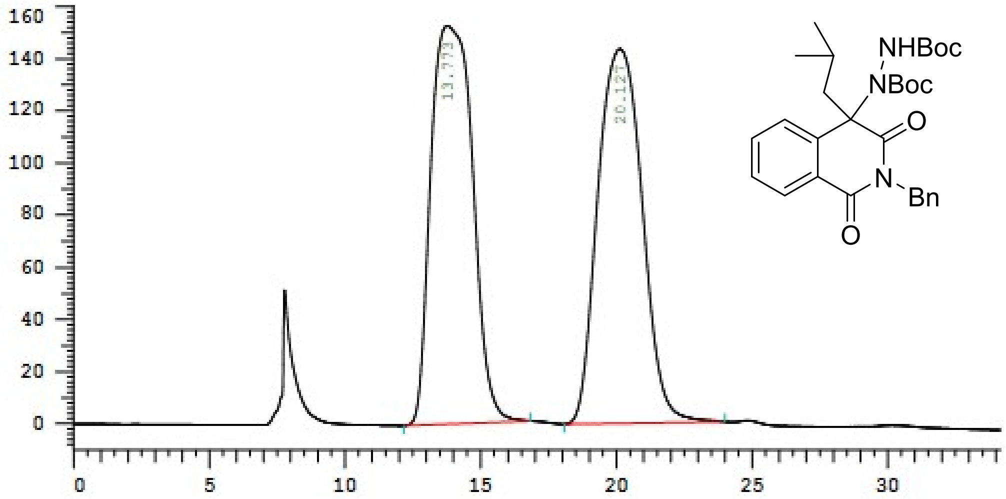

No.	RT	面积	浓度1	BC
1	13.773	16311998	50.003	BB
2	20.127	16310174	49.997	BB
		32622172	100.000	

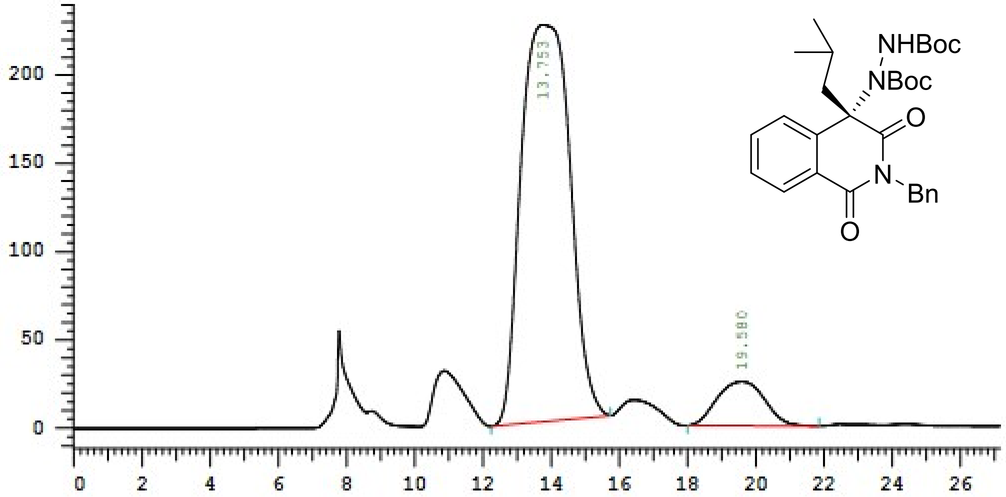

No.	RT	面积	浓度1	BC
1	13.753	22438699	90.281	BB
2	19.580	2415697	9.719	BB
		24854396	100.000	

di-tert-butyl(S)-1-(2-benzyl-1,3-dioxo-4-(3-phenylpropyl)-1,2,3,4-tetrahydroisoquinolin-4-yl)hydrazine-1,2-dicarboxylate(6d):

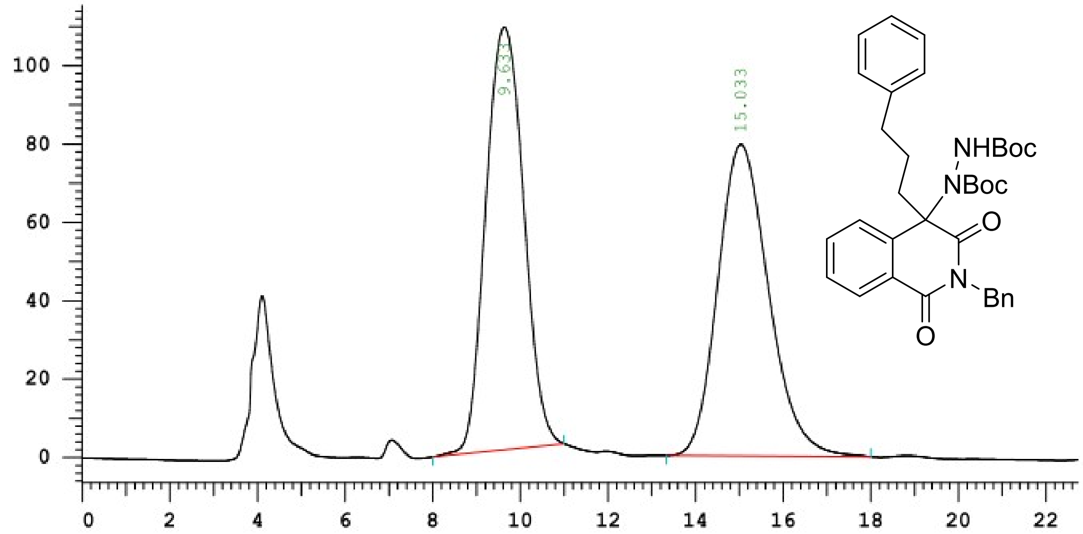

No.	RT	面积	浓度1	BC
1	9.633	6489504	50.343	BB
2	15.033	6401132	49.657	BB
		12890636	100.000	

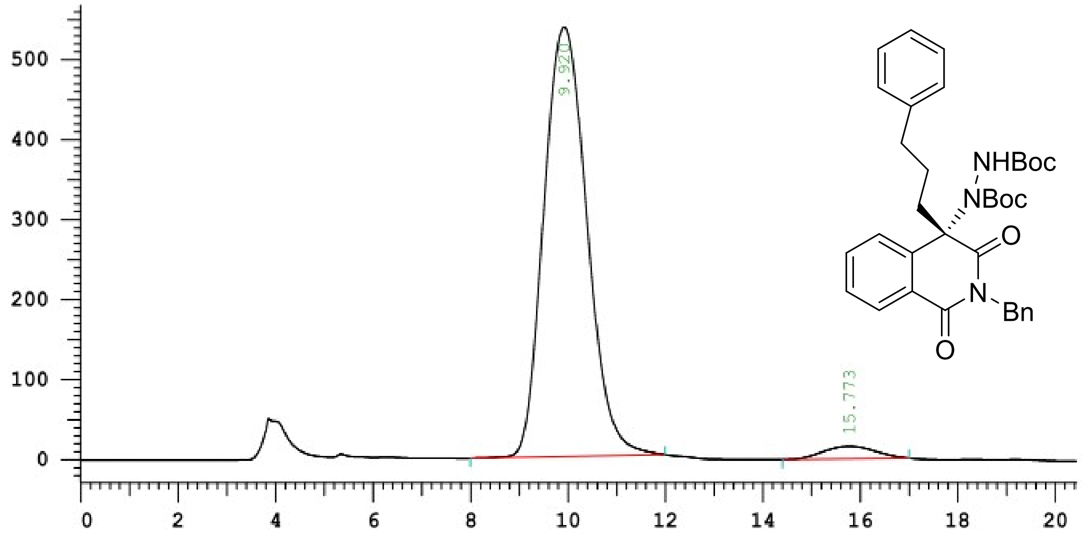

No.	RT	面积	浓度1	BC
1	9.920	33045916	96.613	BB
2	15.773	1158591	3.387	BB
		34204507	100.000	

(di-tert-butyl(S)-1-(2,4-dibenzyl-1,3-dioxo-1,2,3,4-tetrahydroisoquinolin-4-yl)hydrazine-1,2-dicarboxylate (6e):

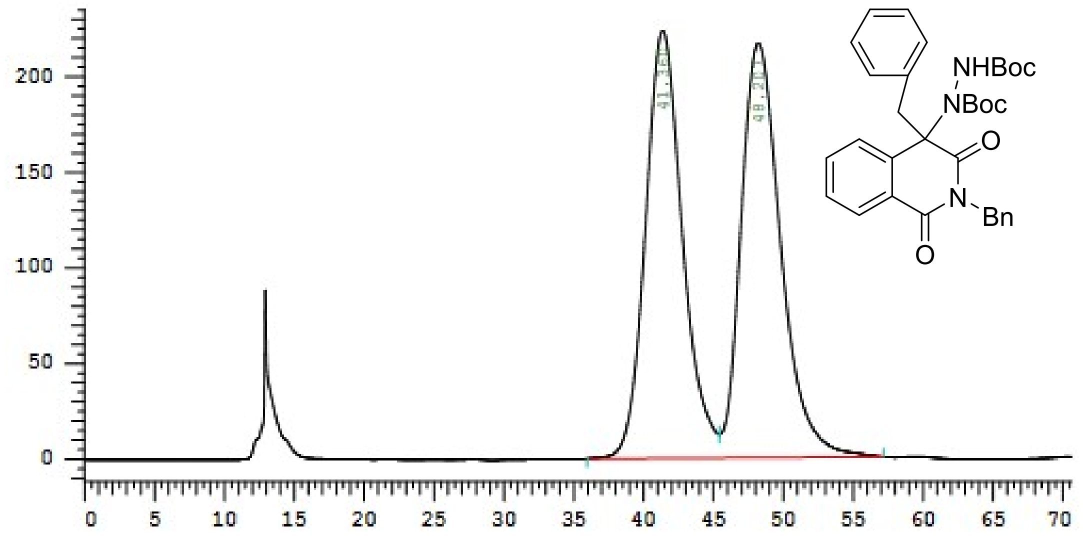

No.	RT	面积	浓度1	BC
1	41.360	41019415	48.974	BV
2	48.207	42738408	51.026	VB
		83757823	100.000	

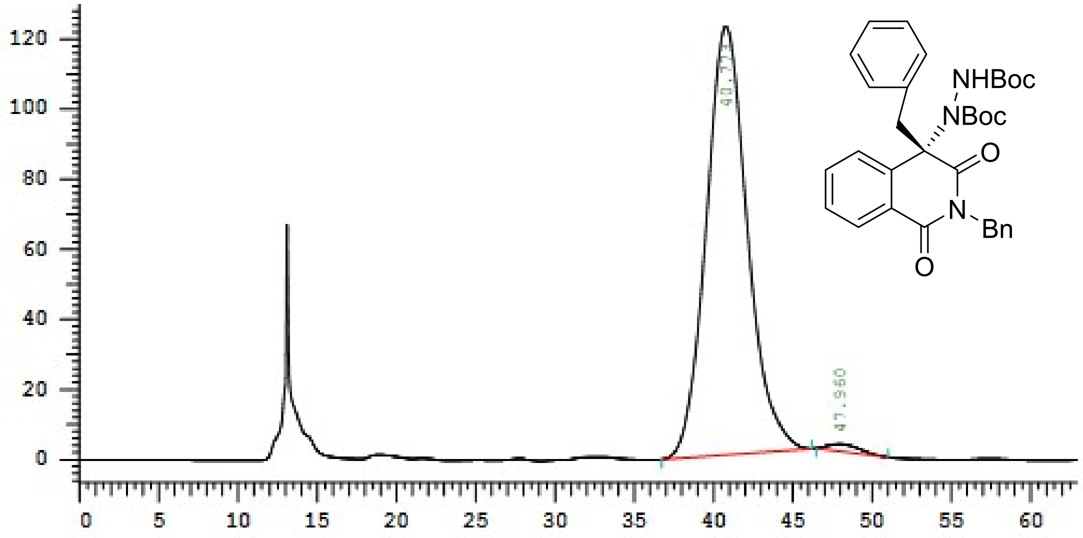

No.	RT	面积	浓度1	BC
1	40.773	22008407	98.724	BB
2	47.960	284378	1.276	BB
		22292785	100.000	

di-tert-butyl(S)-1-(2-benzyl-4-(4-methylbenzyl)-1,3-dioxo-1,2,3,4-tetrahydroisoquinolin-4-yl)hydrazine-1,2-dicarboxylate (6f):

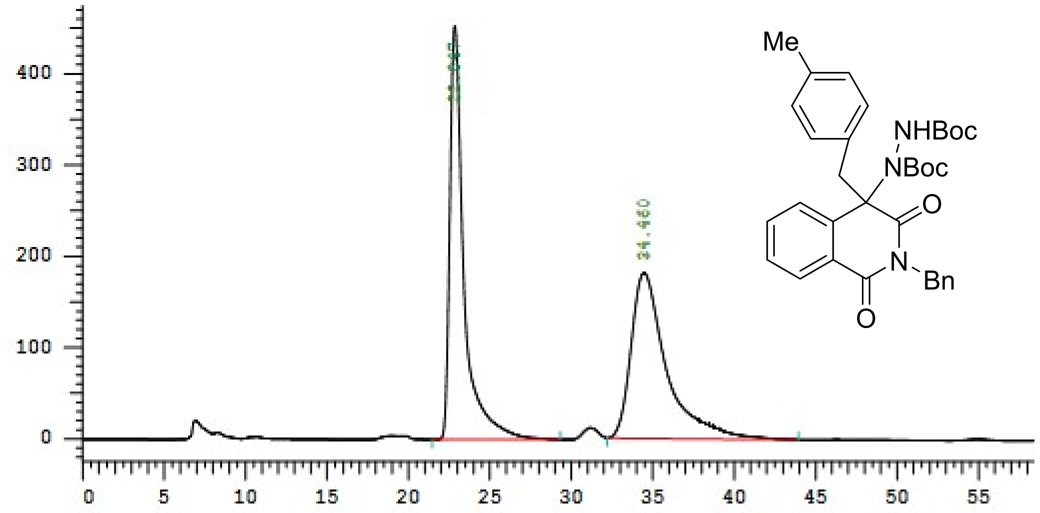

No.	RT	面积	浓度1	BC
1	22.847	27073781	49.454	BB
2	34.460	27671887	50.546	BB
		54745668	100.000	

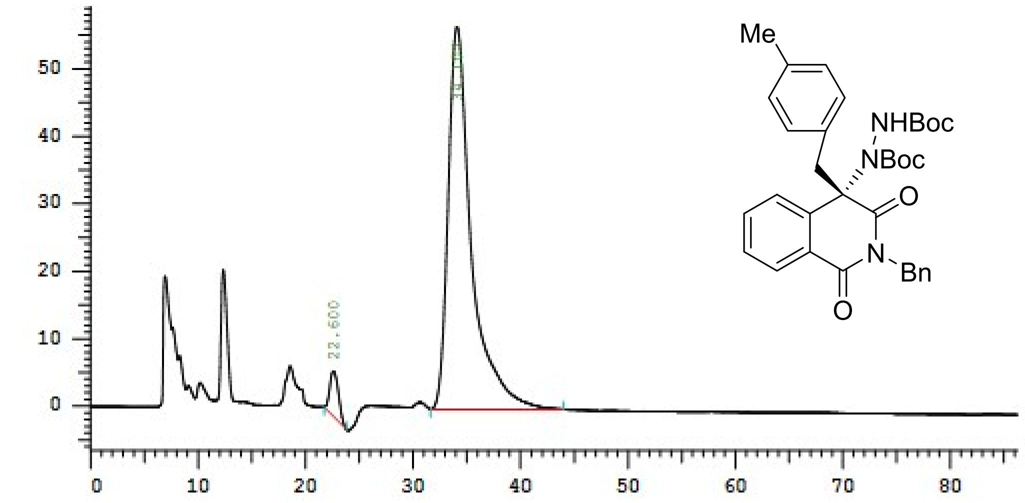

No.	RT	面积	浓度1	BC
1	22.600	377117	4.251	BB
2	34.107	8493604	95.749	BB
		8870721	100.000	

di-tert-butyl(S)-1-(2-benzyl-4-(4-methoxybenzyl)-1,3-dioxo-1,2,3,4-tetrahydroisoquinolin-4-yl)hydrazine-1,2-dicarboxylate (6g):

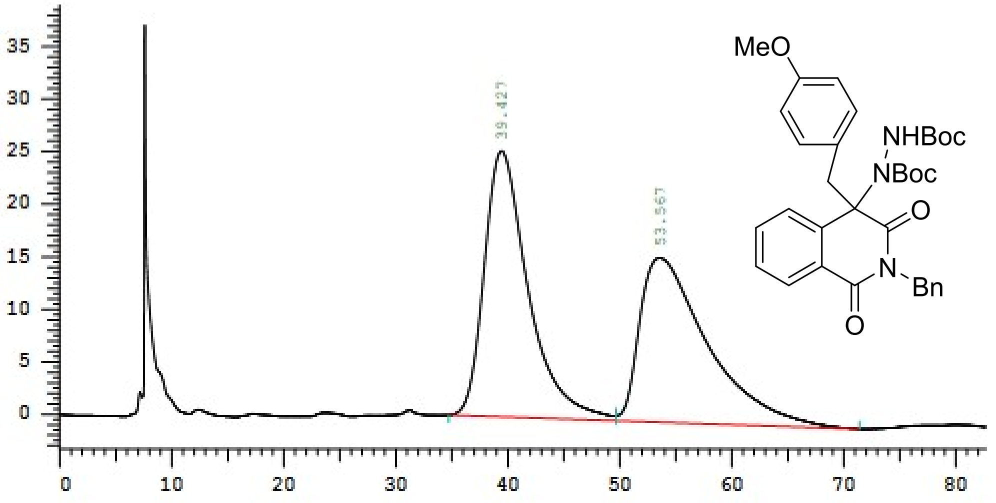

No.	RT	面积	浓度1	BC
1	39.427	6887735	51.105	BV
2	53.567	6589842	48.895	VB
		13477577	100.000	

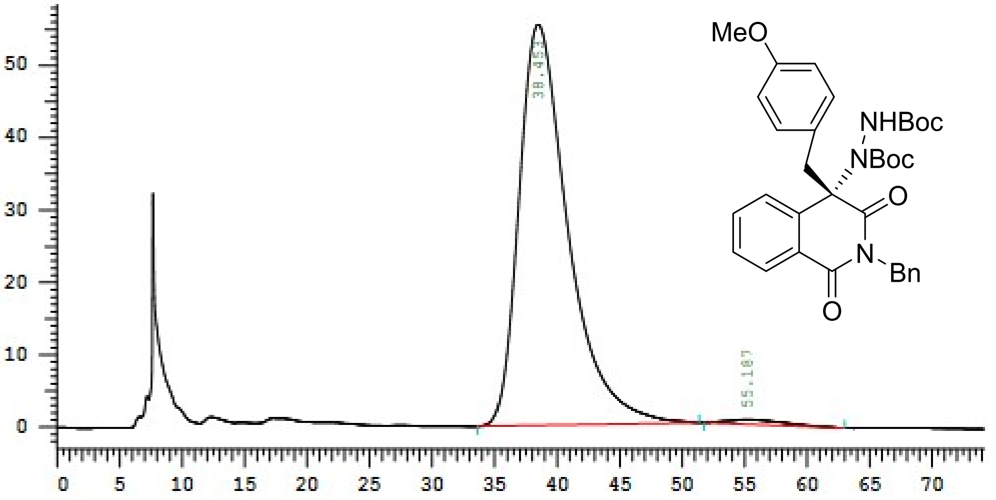

No.	RT	面积	浓度1	BC
1	38.453	15076330	98.578	BB
2	55.187	217472	1.422	BB
		15293802	100.000	

di-tert-butyl(S)-1-(2-benzyl-4-(4-fluorobenzyl)-1,3-dioxo-1,2,3,4-tetrahydroisoquinolin-4-yl)hydrazine-1,2-dicarboxylate (6h):

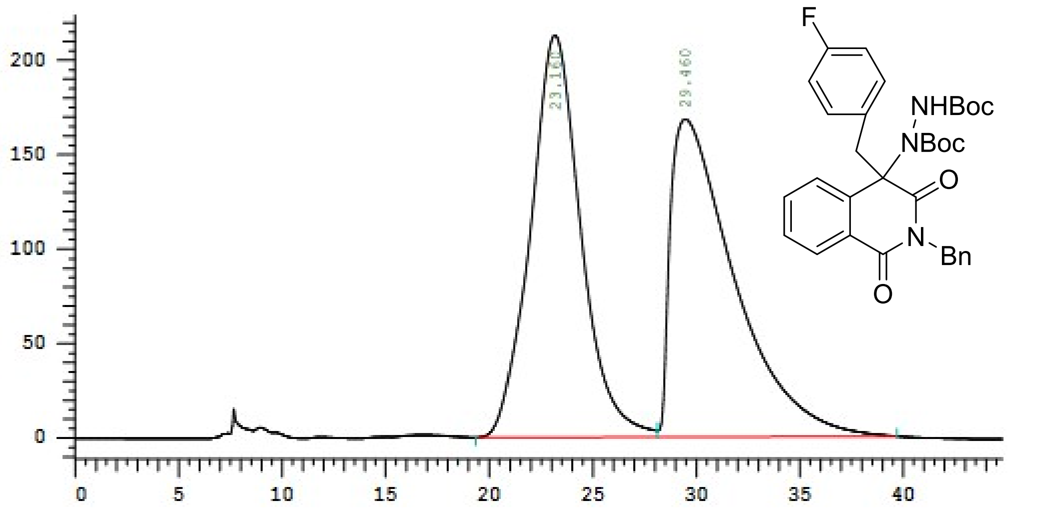

No.	RT	面积	浓度1	BC
1	23.160	35978631	49.853	BV
2	29.460	36190530	50.147	VB
		72169161	100.000	

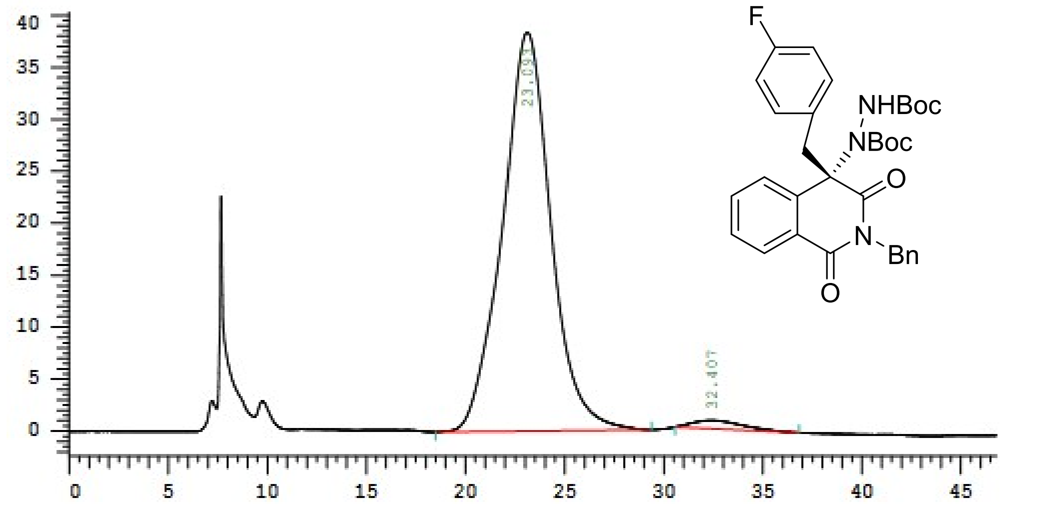

No.	RT	面积	浓度1	BC
1	23.093	6481744	97.837	BB
2	32.407	143279	2.163	BB
		6625023	100.000	

di-tert-butyl(S)-1-(2-benzyl-4-(4-chlorobenzyl)-1,3-dioxo-1,2,3,4-tetrahydroisoquinolin-4-yl)hydrazine-1,2-dicarboxylate(6i):

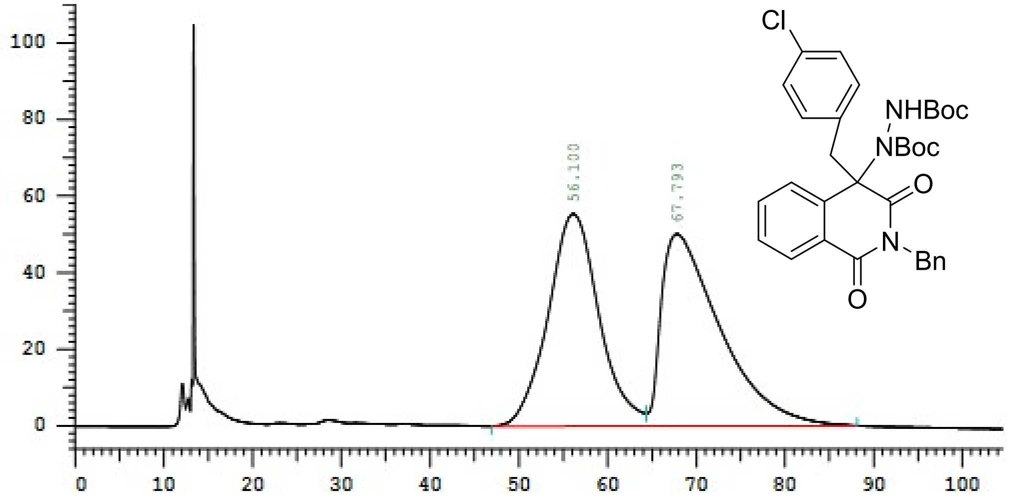

No.	RT	面积	浓度1	BC
1	56.100	22062149	48.698	BV
2	67.793	23242078	51.302	VB
		45304227	100.000	

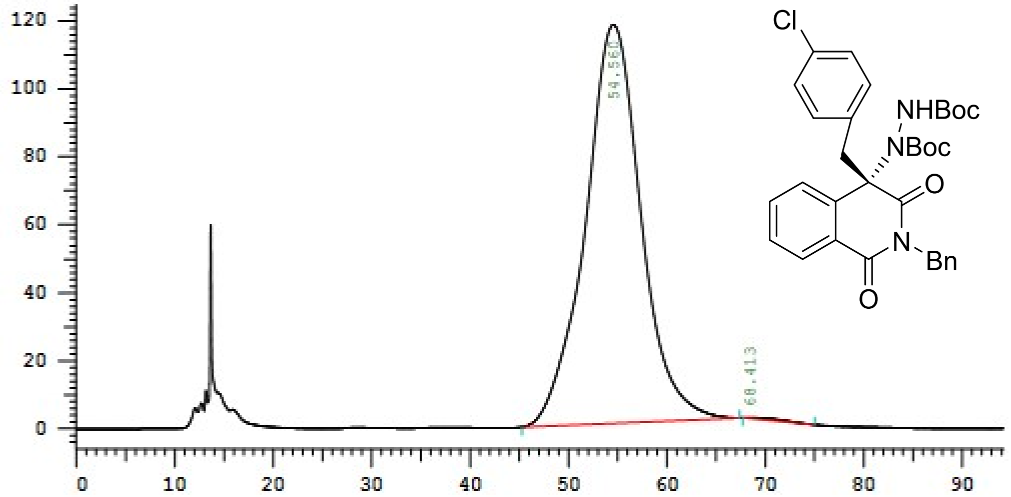

No.	RT	面积	浓度1	BC
1	54.560	45944417	99.731	BB
2	68.413	123743	0.269	BB
		46068160	100.000	

di-tert-butyl(S)-1-(2-benzyl-4-(4-bromobenzyl)-1,3-dioxo-1,2,3,4-tetrahydroisoquinolin-4-yl)hydrazine-1,2-dicarboxylate (6j):

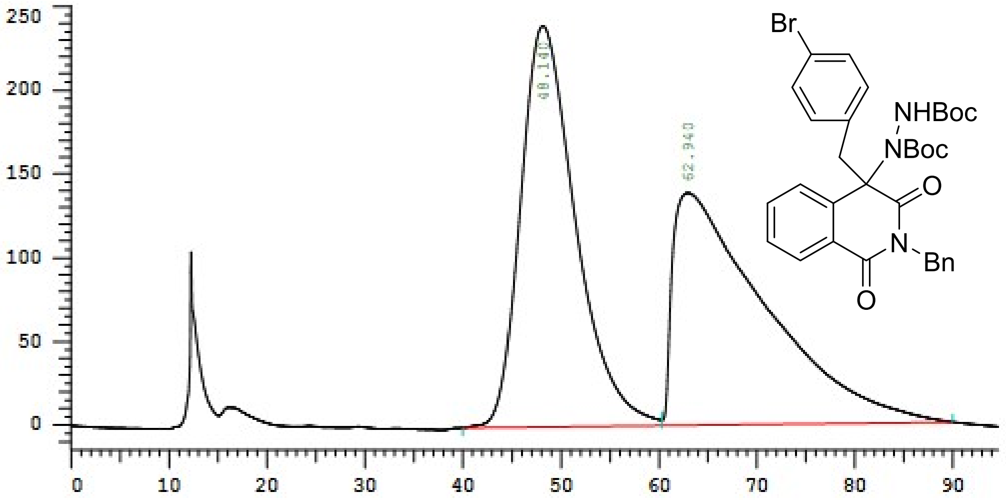

No.	RT	面积	浓度1	BC
1	48.140	93083320	50.444	BV
2	62.940	91443445	49.556	VB
		1.845E+08	100.000	

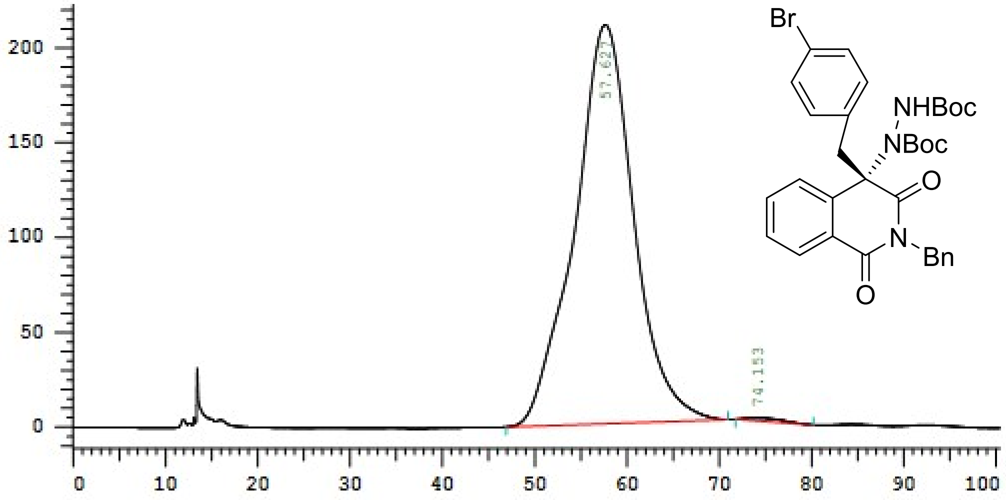

No.	RT	面积	浓度1	BC
1	57.627	93407508	99.478	BB
2	74.153	490329	0.522	BB
		93897837	100.000	

di-tert-butyl(S)-1-(2-benzyl-4-(3-methylbenzyl)-1,3-dioxo-1,2,3,4-tetrahydroisoquinolin-4-yl)hydrazine-1,2-dicarboxylate (6k):

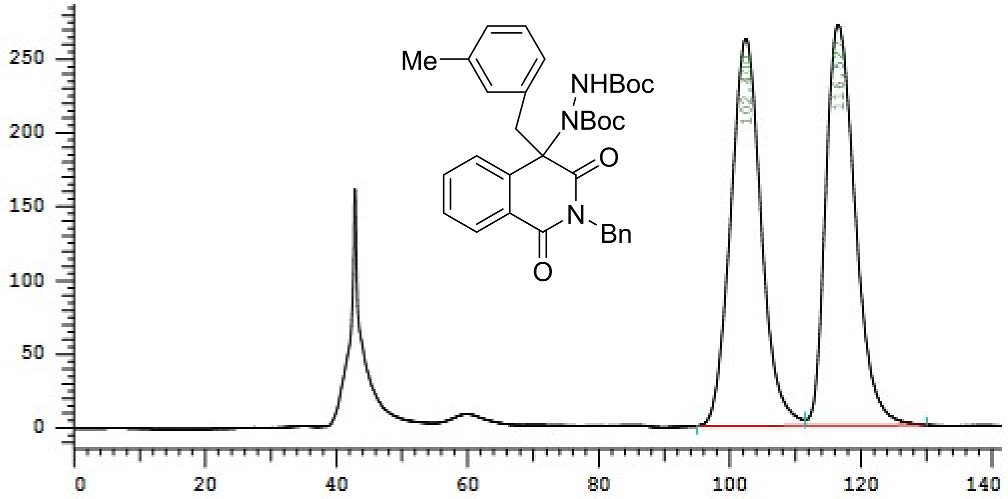

No.	RT	面积	浓度1	BC
1	102.400	84720783	49.957	BV
2	116.527	84865223	50.043	VB
		1.695E+08	100.000	

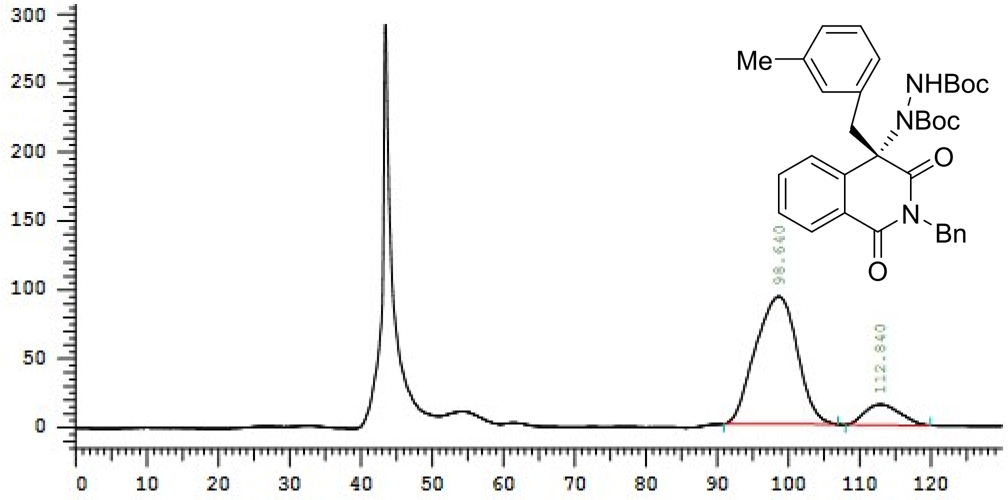

No.	RT	面积	浓度1	BC
1	98.640	37733877	87.988	BB
2	112.840	5151496	12.012	BB
		42885373	100.000	

di-tert-butyl(S)-1-(2-benzyl-4-(3-bromobenzyl)-1,3-dioxo-1,2,3,4-tetrahydroisoquinolin-4-yl)hydrazine-1,2-dicarboxylate (6l):

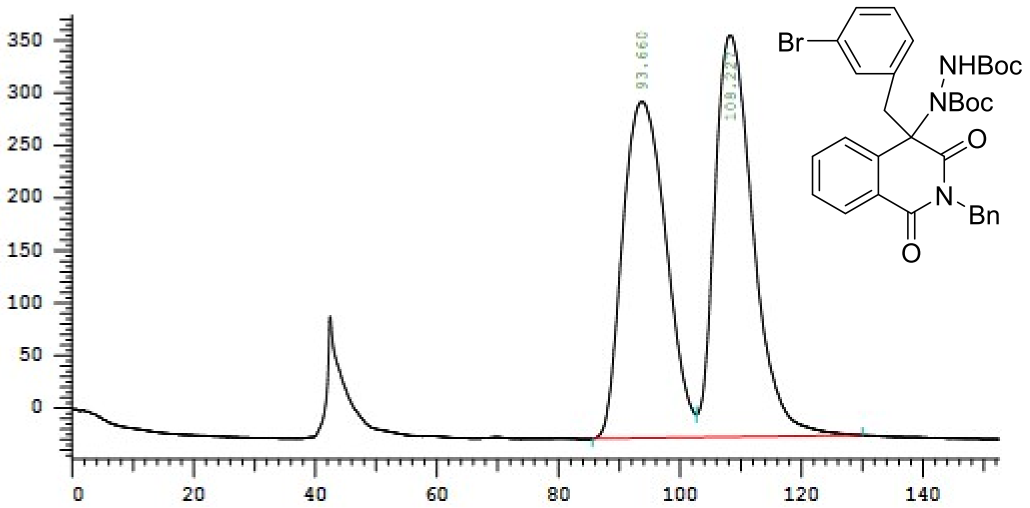

No.	RT	面积	浓度1	BC
1	93.660	1.581E+08	48.538	BV
2	108.227	1.676E+08	51.462	VB
		3.257E+08	100.000	

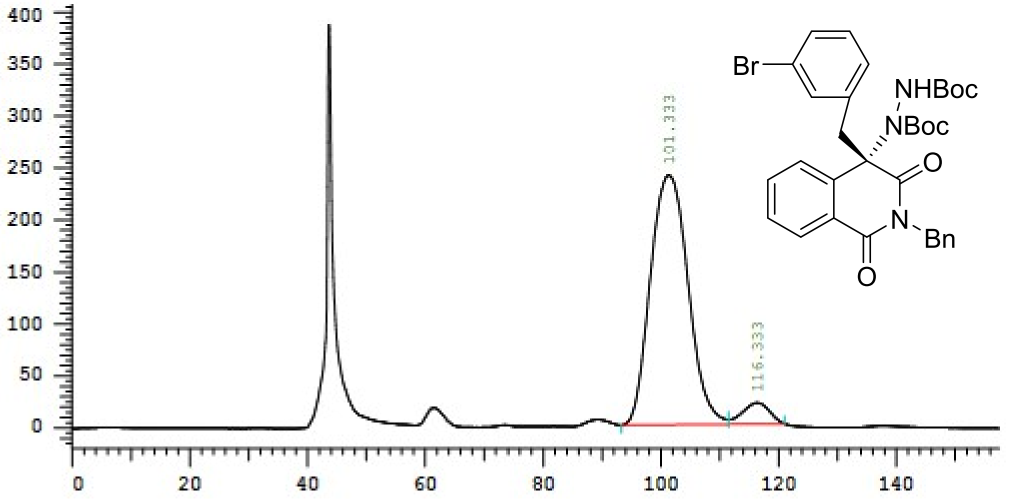

No.	RT	面积	浓度1	BC
1	101.333	1.066E+08	94.276	BV
2	116.333	6475685	5.724	VB
		1.131E+08	100.000	

di-tert-butyl(S)-1-(2-benzyl-4-(2-methoxybenzyl)-1,3-dioxo-1,2,3,4-tetrahydroisoquinolin-4-yl)hydrazine-1,2-dicarboxylate (6m):

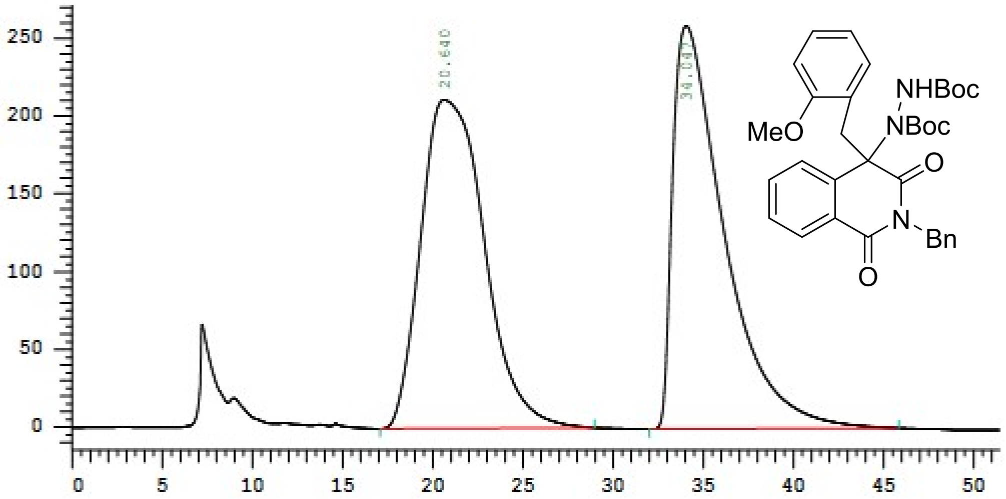

No.	RT	面积	浓度1	BC
1	20.640	51884554	50.220	BB
2	34.047	51429457	49.780	BB
		1.033E+08	100.000	

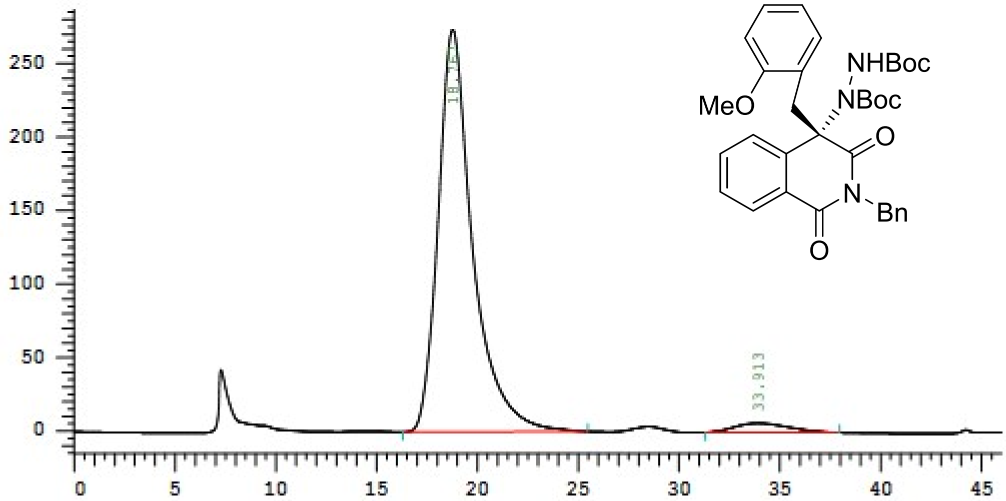

No.	RT	面积	浓度1	BC
1	18.767	32505809	96.442	BB
2	33.913	1199130	3.558	BB
		33704939	100.000	

di-tert-butyl(S)-1-(2-benzyl-4-(2-methylbenzyl)-1,3-dioxo-1,2,3,4-tetrahydroisoquinolin-4-yl)hydrazine-1,2-dicarboxylate (6n):

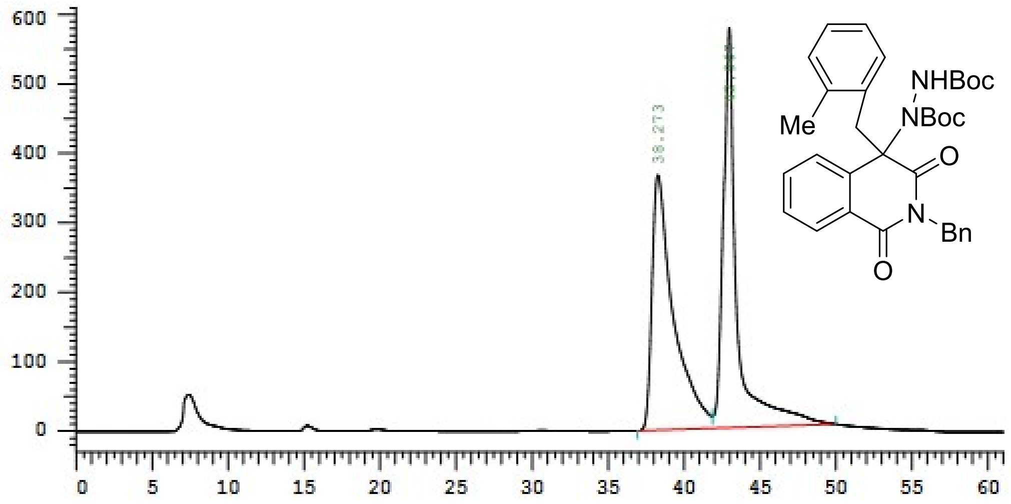

No.	RT	面积	浓度1	BC
1	38.273	36072230	50.113	BV
2	42.967	35909475	49.887	VB
		71981705	100.000	

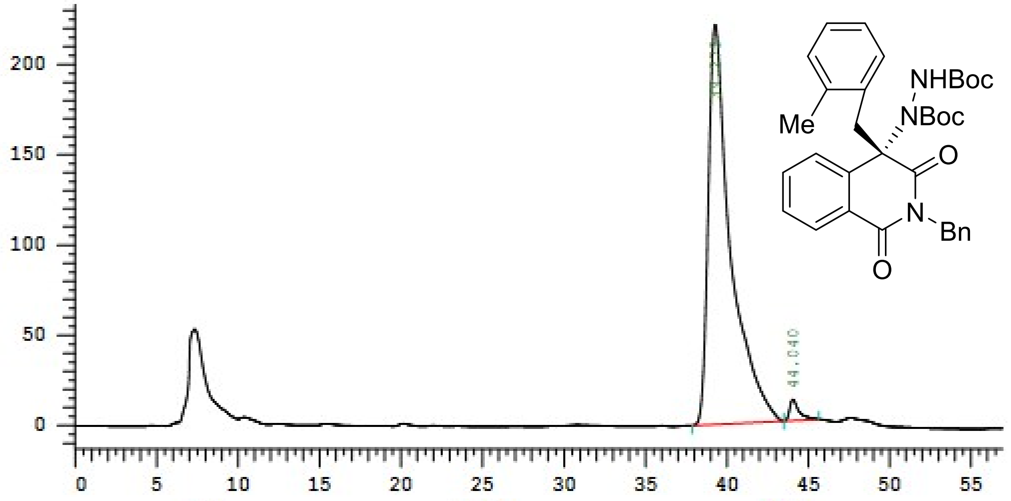

No.	RT	面积	浓度1	BC
1	39.273	21032094	97.834	BB
2	44.040	465561	2.166	BB
		21497655	100.000	

di-tert-butyl(S)-1-(2-benzyl-4-(2-chlorobenzyl)-1,3-dioxo-1,2,3,4-tetrahydroisoquinolin-4-yl)hydrazine-1,2-dicarboxylate (6o):

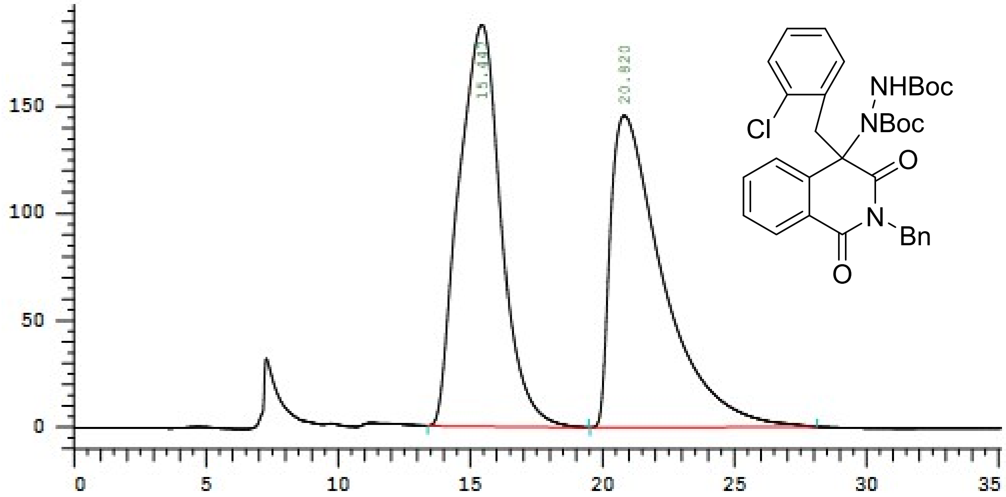

No.	RT	面积	浓度1	BC
1	15.447	20860706	50.747	BB
2	20.820	20246876	49.253	BB
		41107582	100.000	

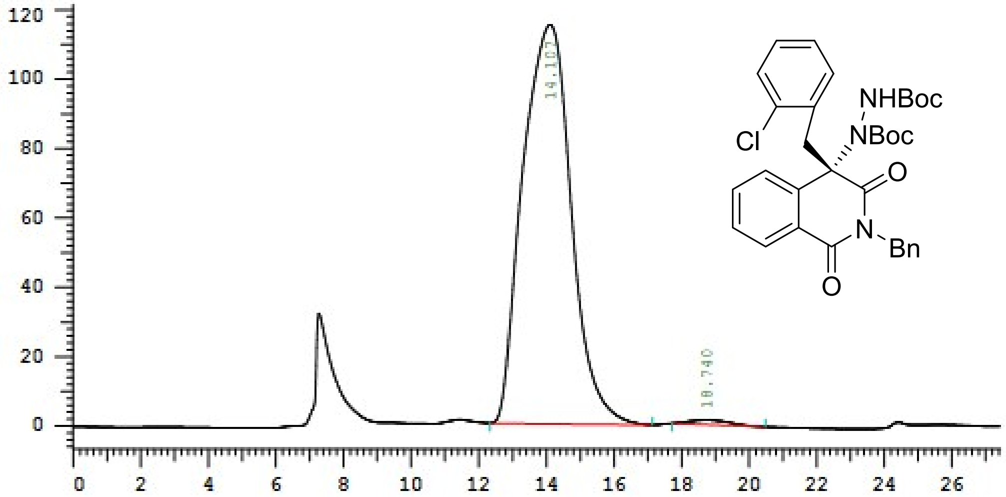

No.	RT	面积	浓度1	BC
1	14.107	11457751	99.020	BB
2	18.740	113382	0.980	BB
		11571133	100.000	

di-tert-butyl(S)-1-(2-benzyl-1,3-dioxo-4-(3,4,5-trimethoxybenzyl)-1,2,3,4-tetrahydroisoquinolin-4-yl)hydrazine-1,2-dicarboxylate (6p):

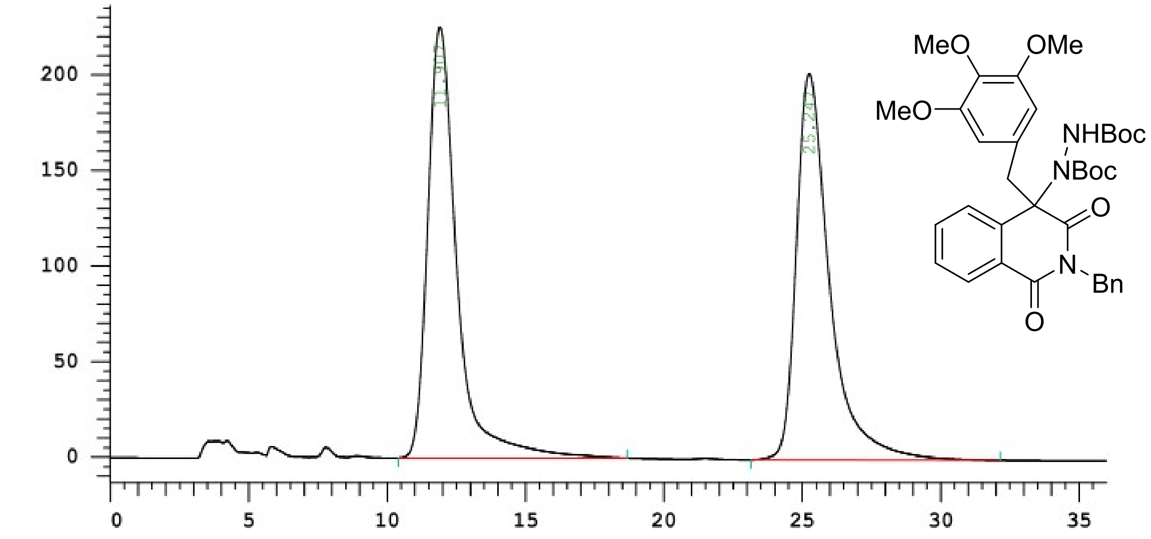

No.	RT	面积	浓度1	BC
1	11.907	16423219	49.240	BB
2	25.247	16930403	50.760	BB
		33353622	100.000	

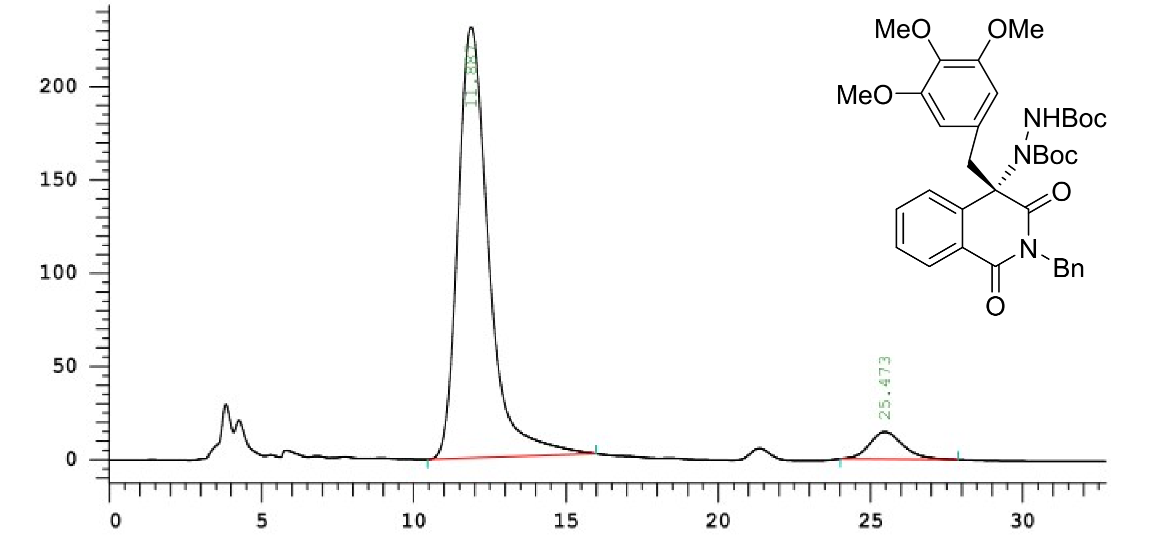

No.	RT	面积	浓度1	BC
1	11.887	16150886	93.483	BB
2	25.473	1125846	6.517	BB
		17276732	100.000	

di-tert-butyl(S)-1-(2-benzyl-4-(naphthalen-2-ylmethyl)-1,3-dioxo-1,2,3,4-tetrahydroisoquinolin-4-yl)hydrazine-1,2-dicarboxylate (6q):

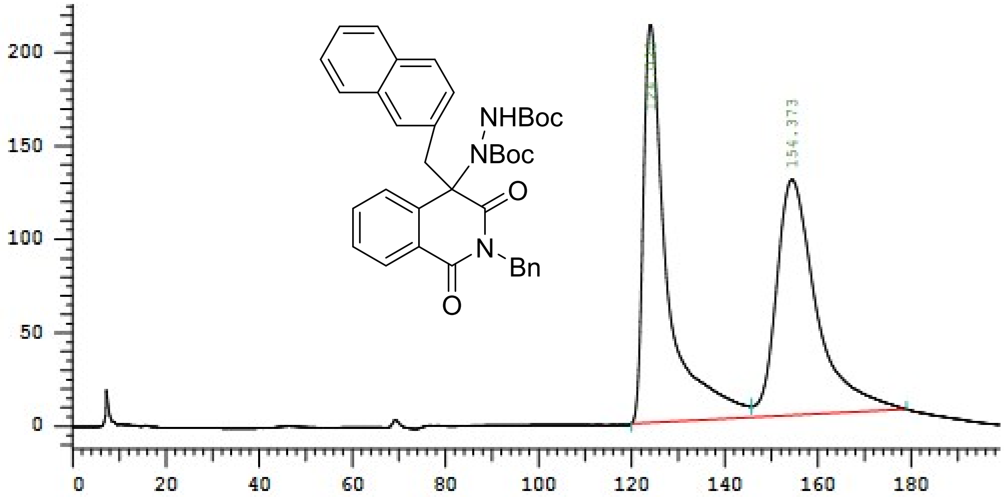

No.	RT	面积	浓度1	BC
1	124.020	75453209	50.013	BV
2	154.373	75415393	49.987	VB
		1.508E+08	100.000	

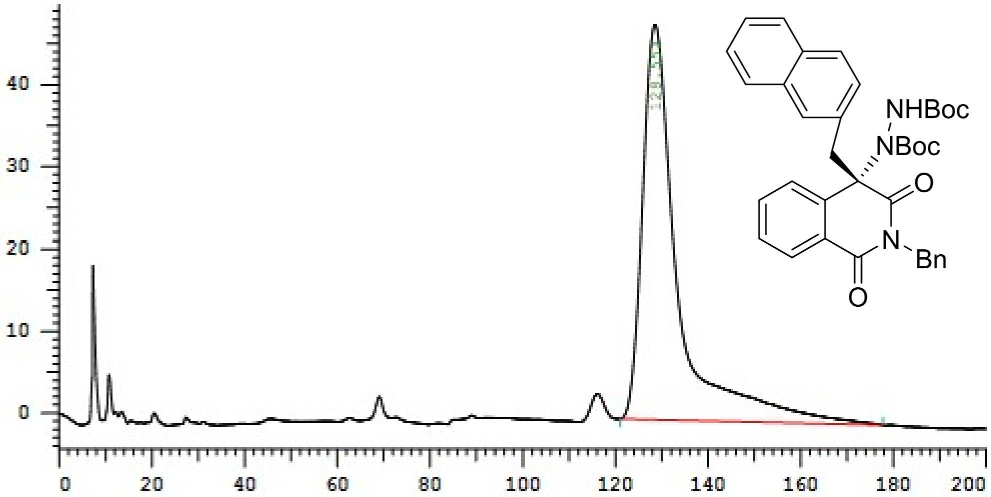

No.	RT	面积	浓度1	BC
1	128.553	25348165	100.000	BB
		25348165	100.000	

di-tert-butyl(S)-1-(4-((1H-indol-2-yl)methyl)-2-benzyl-1,3-dioxo-1,2,3,4-tetrahydroisoquinolin-4-yl)hydrazine-1,2-dicarboxylate (6r):

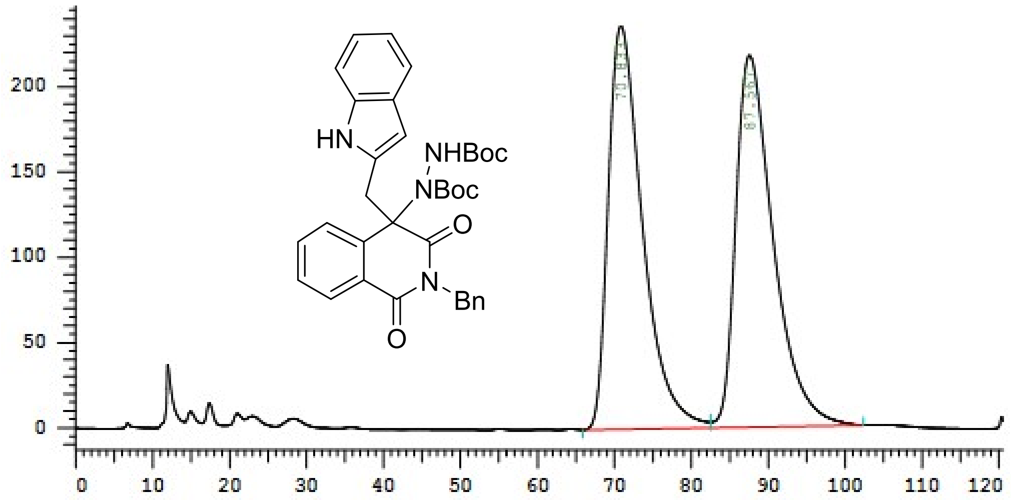

No.	RT	面积	浓度1	BC
1	70.833	73110420	49.886	BV
2	87.567	73445601	50.114	VB
		1.465E+08	100.000	

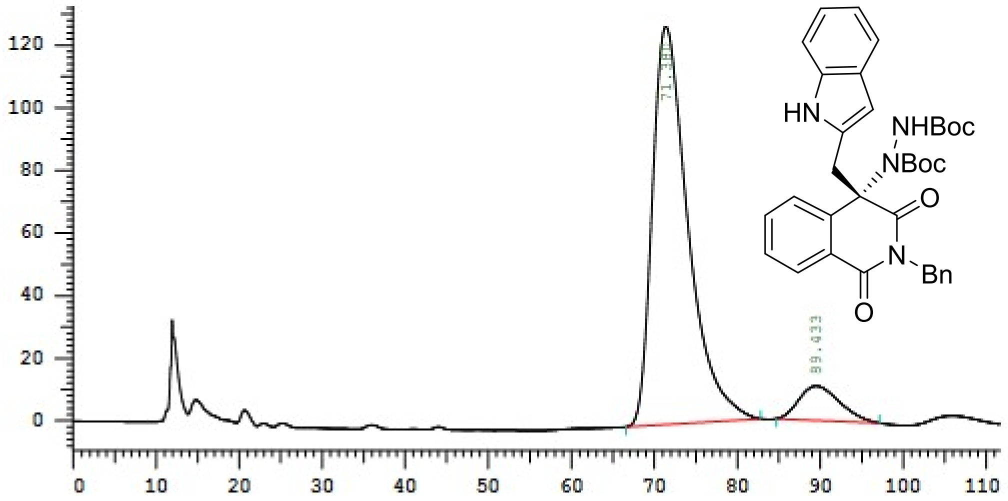

No.	RT	面积	浓度1	BC
1	71.380	37910254	90.984	BB
2	89.433	3756856	9.016	BB
		41667110	100.000	

di-tert-butyl(S)-1-(4-((1H-indol-3-yl)methyl)-2-benzyl-1,3-dioxo-1,2,3,4-tetrahydroisoquinolin-4-yl)hydrazine-1,2-dicarboxylate (6s):

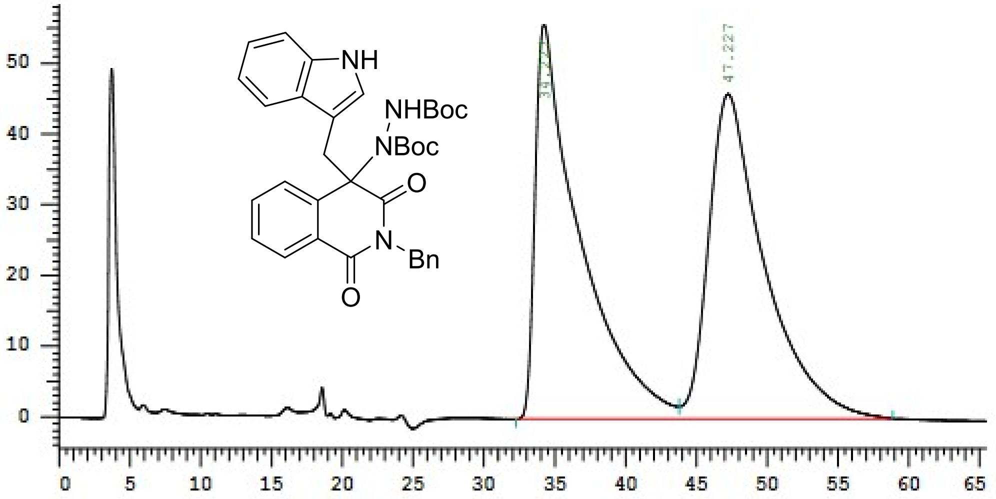

No.	RT	面积	浓度1	BC
1	34.227	12250519	49.312	BV
2	47.227	12592529	50.688	VB
		24843048	100.000	

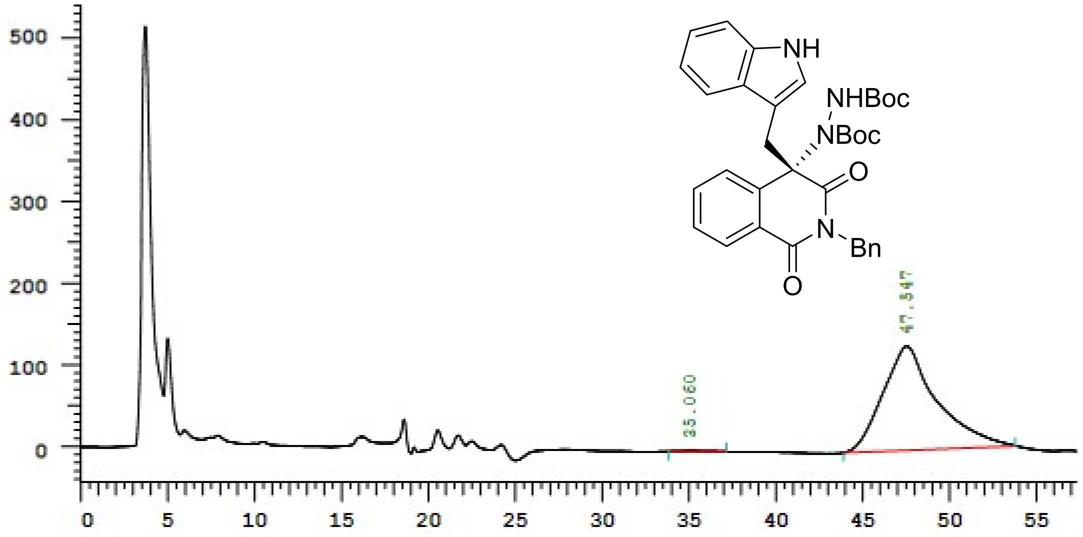

No.	RT	面积	浓度1	BC
1	35.060	9032	0.329	BB
2	47.547	2738711	99.671	BB
		2747743	100.000	

di-tert-butyl(S)-1-(2-benzyl-4-((2-methyl-1H-indol-3-yl)methyl)-1,3-dioxo-1,2,3,4-tetrahydroisoquinolin-4-yl)hydrazine-1,2-dicarboxylate (6t):

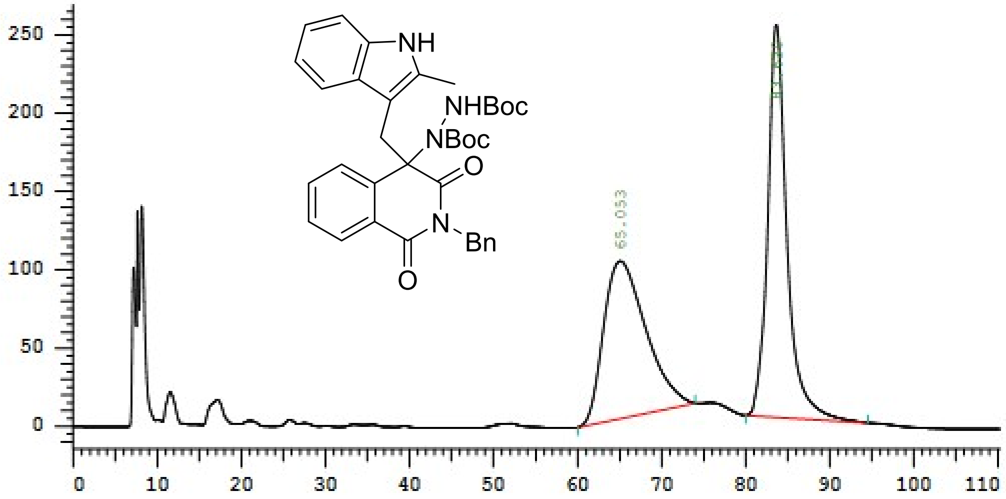

No.	RT	面积	浓度1	BC
1	65.053	35118993	47.030	BB
2	83.620	39554465	52.970	BB
		74673458	100.000	

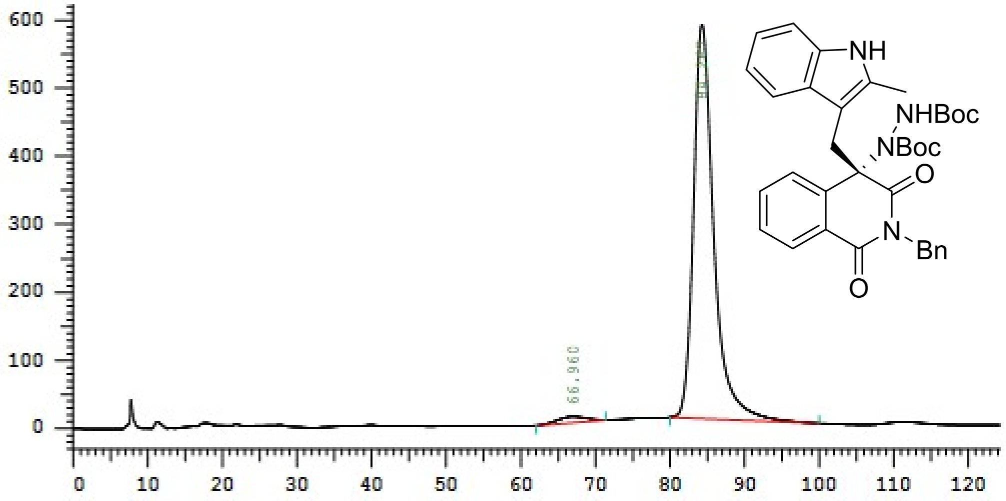

No.	RT	面积	浓度1	BC
1	66.960	2468924	2.223	BB
2	84.267	1.085E+08	97.777	BB
		1.110E+08	100.000	

di-tert-butyl(S)-1-(2-benzyl-4-(furan-2-ylmethyl)-1,3-dioxo-1,2,3,4-tetrahydroisoquinolin-4-yl)hydrazine-1,2-dicarboxylate (6u):

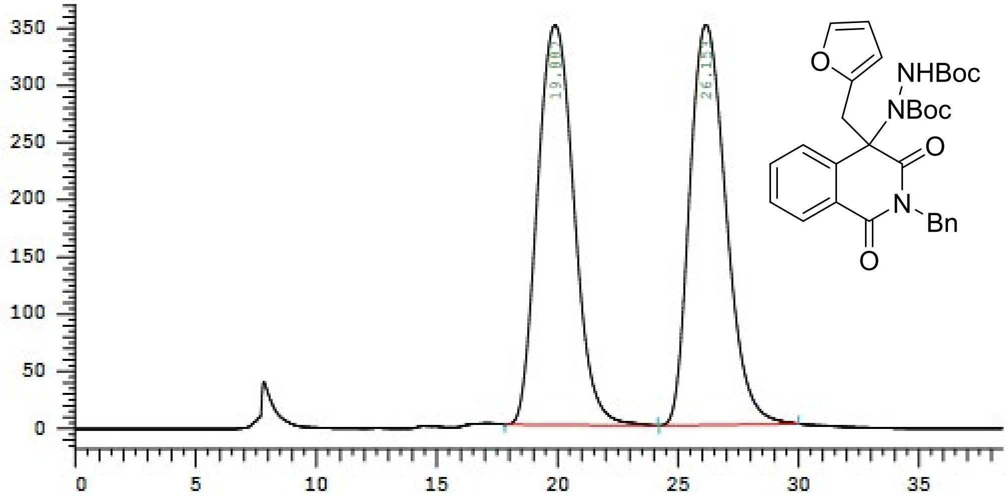

No.	RT	面积	浓度1	BC
1	19.887	36813404	50.118	BB
2	26.153	36639884	49.882	BB
		73453288	100.000	

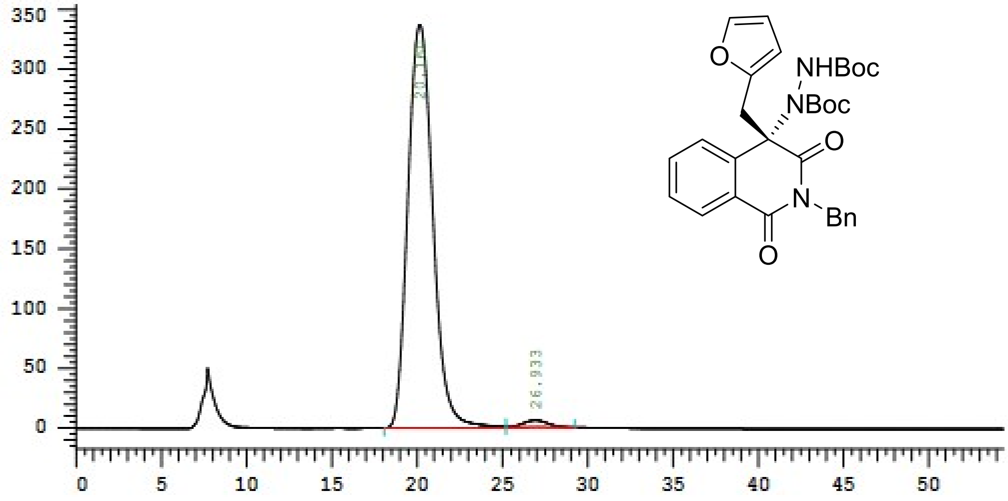

No.	RT	面积	浓度1	BC
1	20.160	34255408	98.473	BV
2	26.933	531333	1.527	TBB
		34786741	100.000	

diethyl (S)-1-(2-benzyl-4-butyl-1,3-dioxo-1,2,3,4-tetrahydroisoquinolin-4-yl)hydrazine-1,2-dicarboxylate(6v):

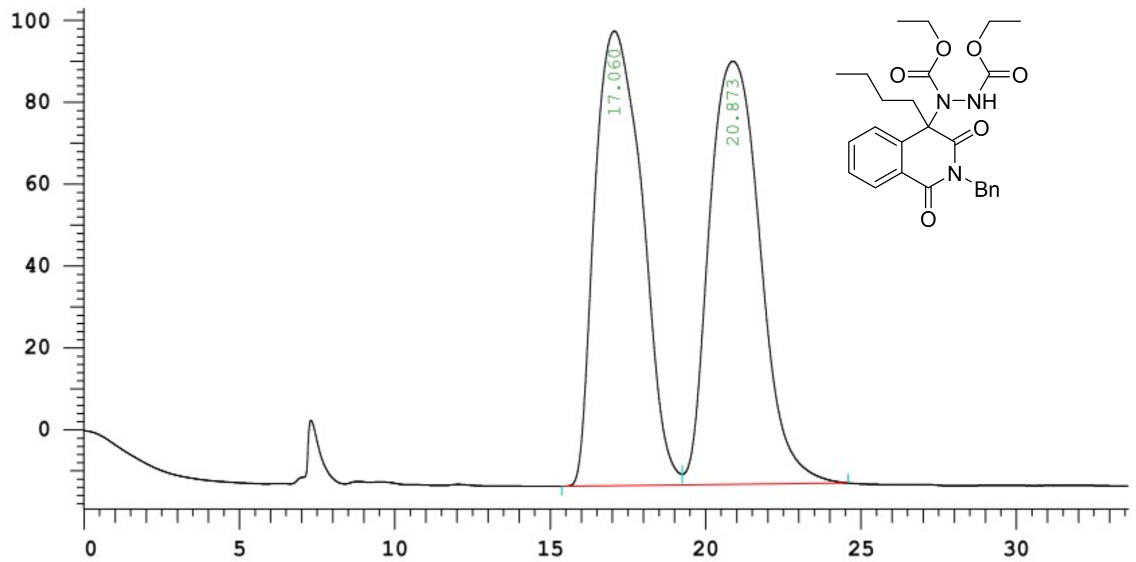

No.	RT	面积	浓度1	BC
1	17.060	11719642	49.925	BV
2	20.873	11755048	50.075	VB
		23474690	100.000	

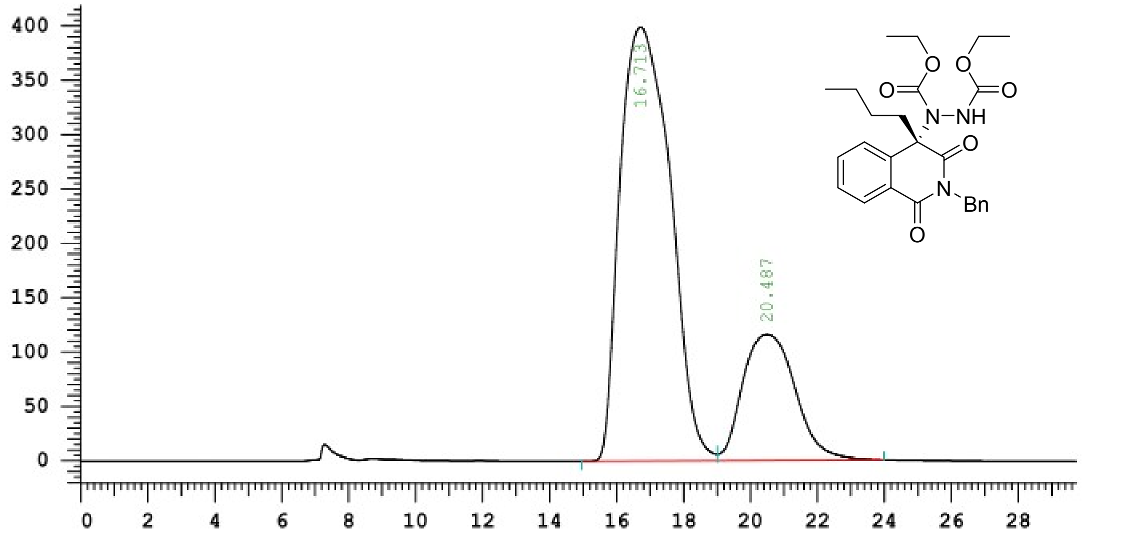

No.	RT	面积	浓度1	BC
1	16.713	41676756	76.677	BV
2	20.487	12677093	23.323	VB
		54353849	100.000	

diisopropyl (S)-1-(2-benzyl-4-butyl-1,3-dioxo-1,2,3,4-tetrahydroisoquinolin-4-yl)hydrazine-1,2-dicarboxylate(6w):

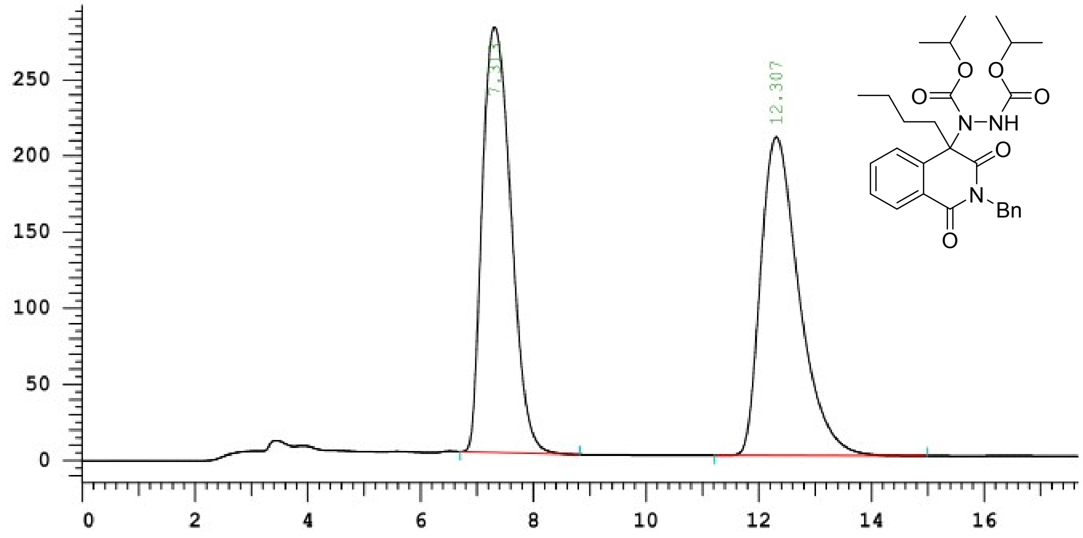

No.	RT	面积	浓度1	BC
1	7.313	9882017	49.977	BB
2	12.307	9891009	50.023	BB
		19773026	100.000	

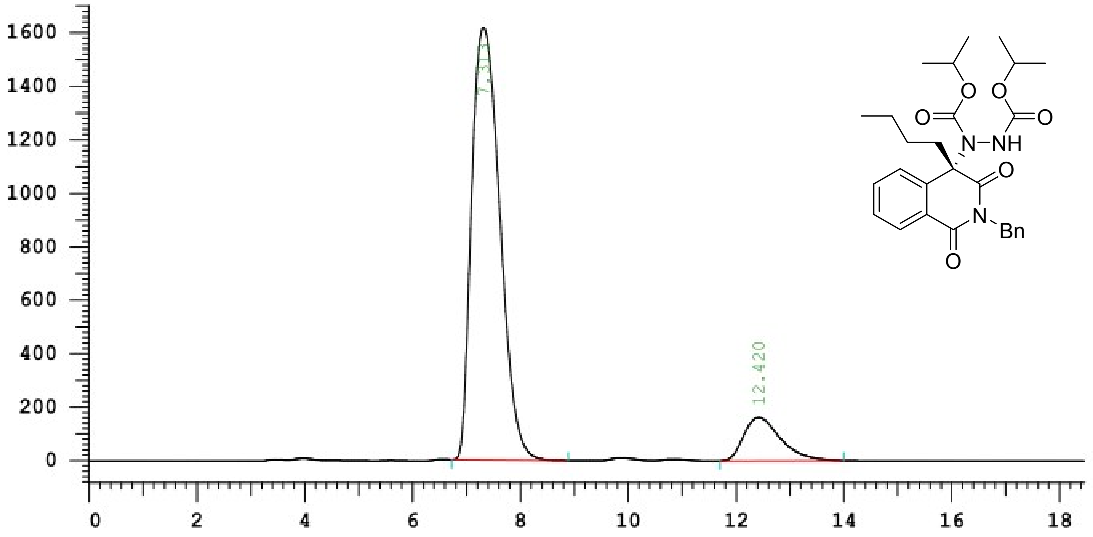

No.	RT	面积	浓度1	BC
1	7.313	58876119	88.420	BB
2	12.420	7710608	11.580	BB
		66586727	100.000	

(R)-N-benzyl-1-(4-chlorobenzyl)-3-oxoisoindoline-1-carboxamide (7):

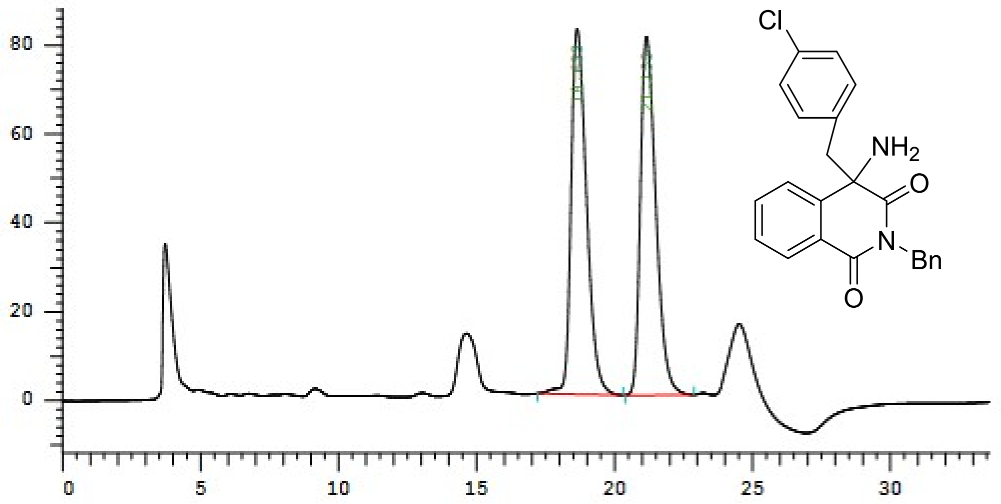

No.	RT	面积	浓度1	BC
1	18.633	3208161	50.566	BB
2	21.153	3136288	49.434	BB
		6344449	100.000	

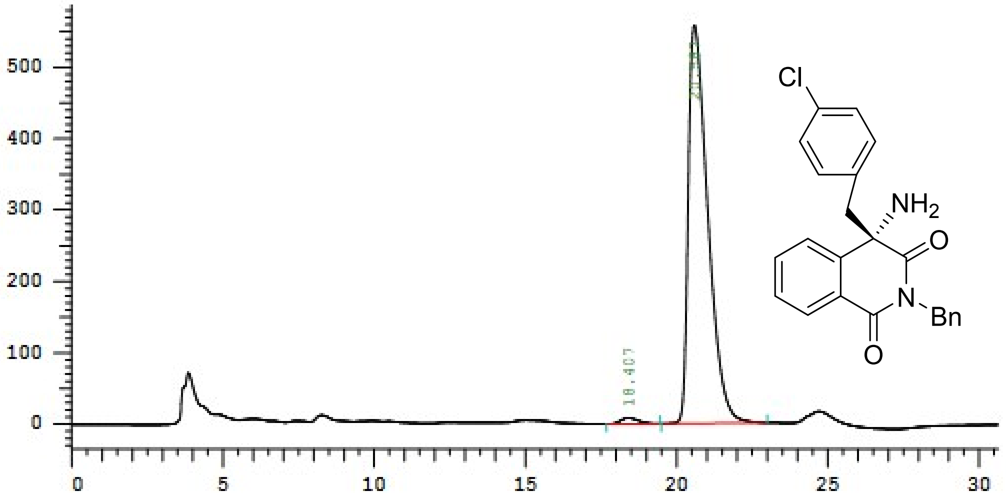

No.	RT	面积	浓度1	BC
1	18.407	333545	1.321	BB
2	20.587	24909027	98.679	BB
		25242572	100.000	

10. References:

1. Guo Yang, Yingxian Li, Cheng Cheng, Shun Zhou, Dandan Zheng, Yuanyong Yang, Lei Tang, Synthesis of monosubstituted Isoquinoline-1,3(2H,4H)-diones. *Science Technology and Engineering*, **2019**, 9, 10−14.

2. Weihong Liang, Wenhao Yin, Tingzhong Wang, Fayang G. Qiu, Junling Zhao. Organocatalytic Stereoselective Conjugate Addition of 3-Substituted Oxindoles with In Situ Generated ortho-Quinone Methides. *Tetrahedron Letters*, **2018**, 59, 1742-1747.
